# Asymmetrically Substituted *m*‐Terphenyl Phosphates Inhibit the Transcription Factor STAT5a

**DOI:** 10.1002/cbic.202100603

**Published:** 2021-12-29

**Authors:** Daniel Müller‐Klieser, Thorsten Berg

**Affiliations:** ^1^ Institute of Organic Chemistry Leipzig University Johannisallee 29 04103 Leipzig Germany

**Keywords:** cross coupling, inhibitors, protein-protein interactions, SH2 domains, transcription factors

## Abstract

We recently presented Stafia‐1 as the first chemical entity that inhibits the transcription factor STAT5a with selectivity over the highly homologous STAT5b. Stafia‐1, which was identified from a series of symmetrically substituted *m*‐terphenyl phosphates, binds to the interface between the SH2 domain and the linker domain of STAT5a. Here, we outline a synthetic strategy for the synthesis of asymmetrically substituted *m*‐terphenyl phosphates, which can be tailored to address their asymmetric STAT5a binding site in a more specific manner. The asymmetrically substituted *m*‐terphenyl phosphate with the highest activity against STAT5a was converted to a phosphatase‐stable monofluoromethylene phosphonate. The synthetic methodology and activity analysis described here provide first insights into the structure‐activity relationships of *m*‐terphenyl phosphates for use as selective STAT5a inhibitors.

## Graphical abstract

**Symmetry is overrated**: Stafia‐1, the first selective inhibitor of the transcription factor STAT5a, is a symmetrically substituted *m*‐terphenyl phosphate that binds to an unsymmetrical protein binding pocket. We present synthetic methodology for generating asymmetrically substituted *m*‐terphenyl phosphate‐based STAT5a inhibitors, which give insights into structure‐activity relationships.

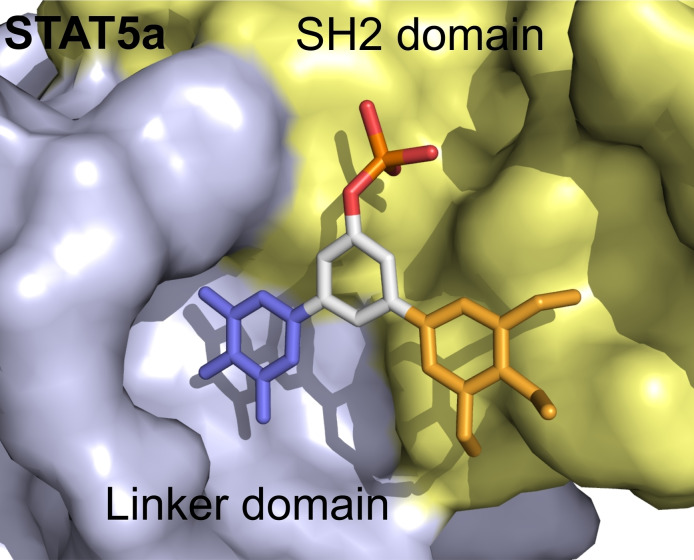

STATs are a family of latent cytoplasmic transcription factors that convey extracellular signals to the nucleus.^[1]^ All of the seven STAT family members are involved in human diseases.^[2]^ The two closely‐related family members STAT5a and STAT5b are constitutively activated in many human tumors. Despite the very high degree of amino acid sequence homology of 96 %,^[3]^ STAT5a and STAT5b have some non‐redundant functions.^[4]^ Selective inhibitors of either STAT5a or STAT5b can serve as valuable tools for analyzing the molecular origin of the non‐redundant functions of the two STAT5 proteins.

We recently presented catechol bisphosphates as the first chemical entities that inhibit STAT5b with selectivity over STAT5a.^[5]^ Subsequently, we presented symmetrically substituted *m*‐terphenyl phosphates as the first inhibitors of STAT5a which display selectivity over STAT5b.^[6]^ The most potent and selective *m*‐terphenyl phosphate‐based STAT5a inhibitor was dubbed Stafia‐1 (**1**, Figure 1A), which inhibited STAT5a (K_i_=10.9±1.8 μM) with at least 9‐fold selectivity over STAT5b.^[6]^ Selective inhibition of STAT5a has been proposed as a therapeutic approach against age‐related osteoporosis.^[7]^

**Figure 1:**
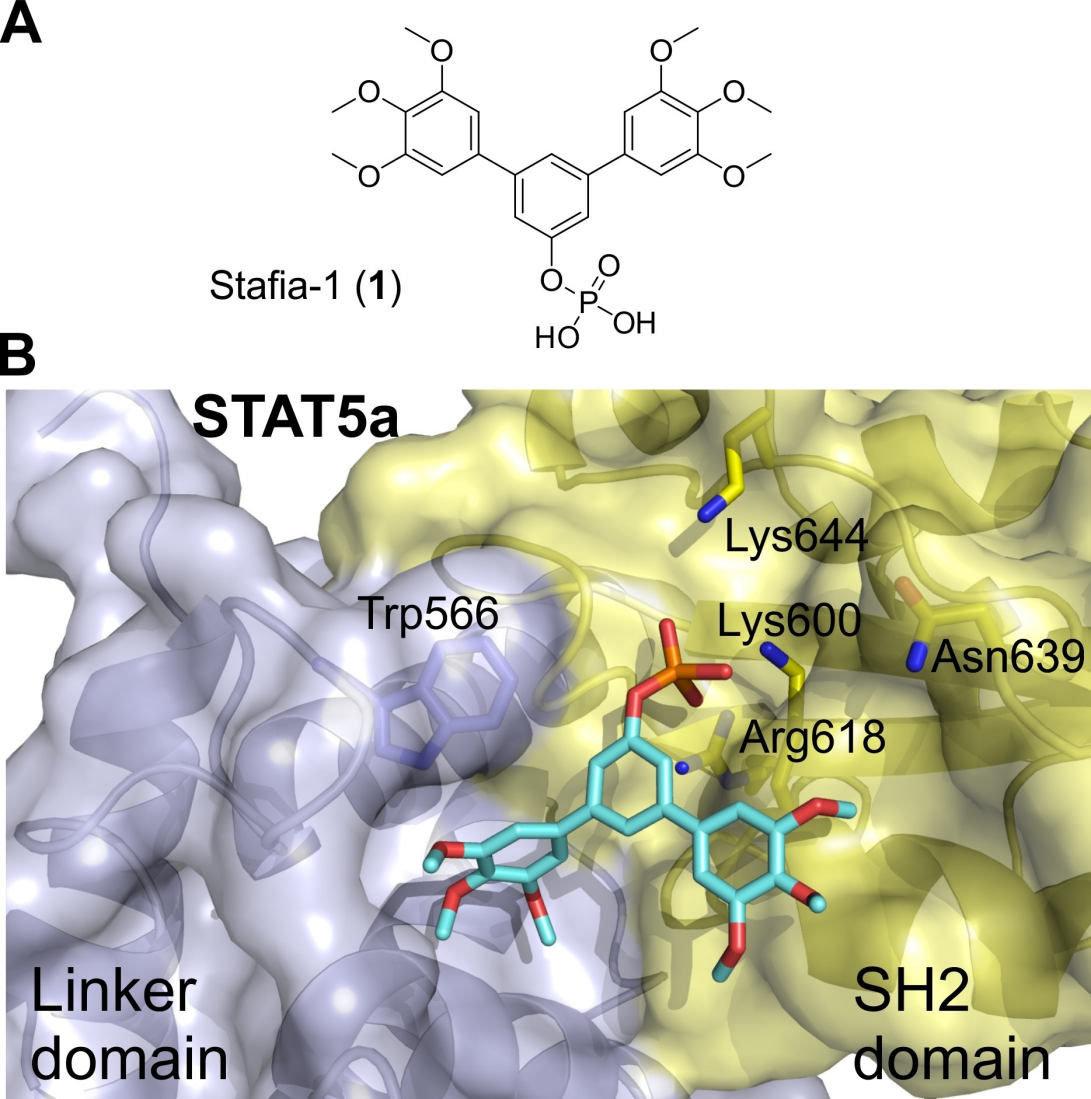
A) Structure of Stafia‐1 and B) its suggested docking‐based mode of binding^[6]^ to the X‐ray structure of murine STAT5a (PBD 1Y1U).^[8]^ The SH2 domain is shown in yellow, the linker domain is shown in light blue.^[10]^ In the docking process, side chain flexibility was allowed for the STAT5a/b‐conserved amino acids Lys600 and Arg618, and for the STAT5a/b‐divergent amino acids Trp566, Asn639, and Lys644.^[6]^

A putative binding mode of Stafia‐1, which is consistent with comparative activity analysis between wild‐type STAT5a/b and point mutants in fluorescence polarization‐based binding assays, was visualized by flexible docking into the crystal structure of STAT5a^[8]^ using AutoDock FR (Figure 1B).^[9]^ The phenyl phosphate group is predicted to engage in electrostatic interactions with Lys600 and Arg618, which are conserved in all STAT proteins. While one of the trimethoxy‐substituted phenyl rings is predicted to bind to the STAT5a SH2 domain, the other one is predicted to bind to the linker domain (Figure 1B).^[6]^

Given that the putative binding pockets of the two outer phenyl rings are not identical, it seemed unlikely that the binding potential of both protein subpockets is fully exploited by a symmetrically substituted *m*‐terphenyl phosphate. Instead, the binding pose suggested the synthesis of asymmetrically substituted *m*‐terphenyl phosphates, which meet the particular requirements for optimal binding to each individual protein subpocket, as an approach by which to develop more potent and selective inhibitors of STAT5a. As a first step towards optimized STAT5a inhibitors, we decided to keep one of the two trimethoxy‐substituted phenyl rings unchanged, whilst varying the substituents on the other aryl ring.

Synthetic access to asymmetrically substituted *m*‐terphenyls was achieved by two consecutive Suzuki couplings based on 3‐bromo‐5‐iodophenol (**2**).^[11]^ The reactivity difference between the iodo‐ and the bromo‐substituted positions was sufficient to allow for selective coupling with 3,4,5‐trimethoxyphenyl boronic acid at the iodo‐substituted position using Pd(PPh_3_)_4_ as a catalyst in 80 % yield. Bromophenol **3** then served as the common intermediate in the following Suzuki couplings that introduce the second phenyl ring bearing variable substitution patterns. This approach is related to the synthesis of *p*‐teraryls using 4‐iodophenyl trifluoromethanesulfonate derivatives as central building blocks.^[12]^ Atherton‐Todd phosphorylation^[13]^ of **4 a**–**j** afforded the benzyl‐protected *m*‐terphenyl phosphates **5 a**–**j**, which were hydrogenated to the target compounds **6 a**–**j** (Scheme 1).

**Scheme 1:**
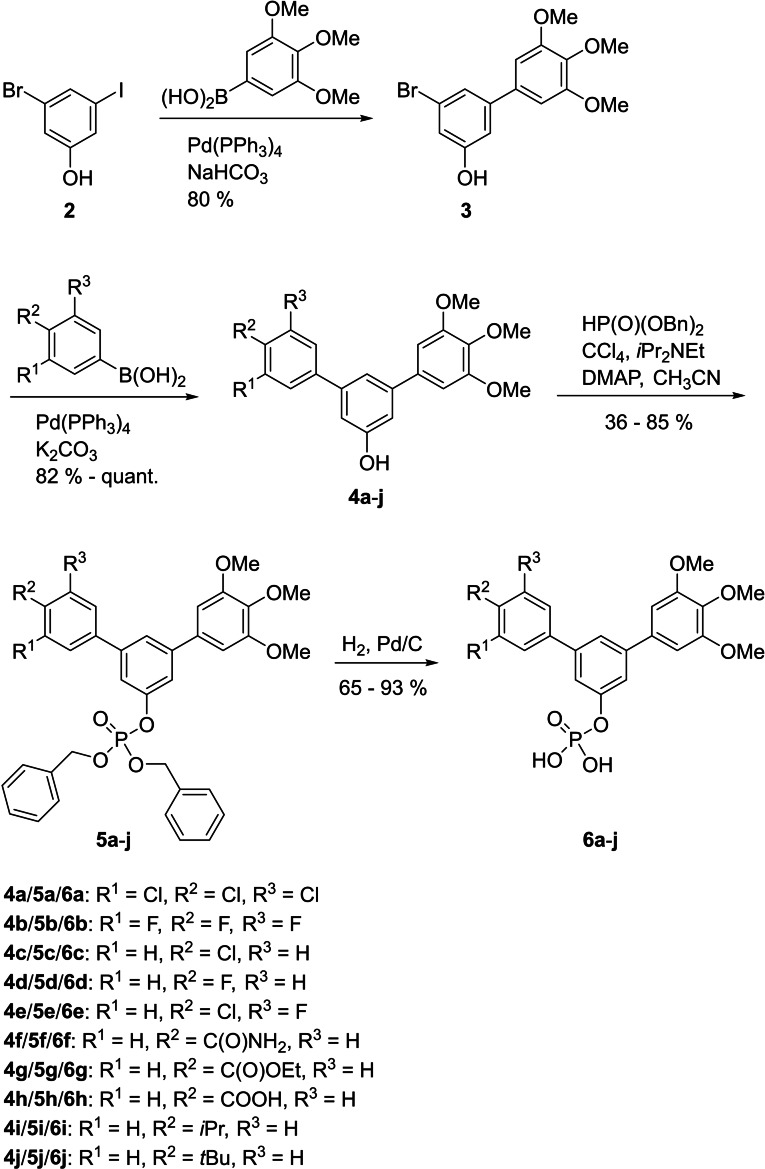
Synthesis of asymmetrically substituted *m*‐terphenyl phosphates **6 a**–**j**.

Since aryl methoxy groups can be metabolically labile,^[14]^ we focused on substituents other than methoxy on the second aryl ring of intermediates **4**. Replacement of all three methoxy groups on the variably substituted phenyl ring by chlorine atoms (**6 a**) maintains activity against STAT5a (K_i_=10.2±0.4 μM) in binding assays based on fluorescence polarization,[^5a^, ^15^] but is associated with a significant loss of selectivity over STAT5b (K_i_=23.1±1.3 μM) as compared to Stafia‐1 [K_i_ (STAT5a)=10.9±1.8 μM; 37±5 % inhibition of STAT5b at 200 μM, the highest concentration tested, Tables 1 and S1]. Replacement of all three methoxy groups on the variable aryl ring by fluorine atoms (**6 b**) also maintains activity against STAT5a (K_i_=10.9±0.2 μM) and is associated with a higher degree of selectivity over STAT5b (K_i_=29.2±1.7 μM) than observed for the triple chlorine substituted compound **6 a**. Compound **6 b** also displayed good selectivity with respect to other STAT family members (Figure 2).

**Table 1:** Structures of *m*‐terphenyl phosphates and activities against STAT5a and STAT5b. K_i_ values were calculated from IC_50_ values (n=3) using the published equation.^[17]^

No.	Structure	STAT5a K_i_ [μM]	STAT5b K_i_ [μM] or inhibition [%] at 200 μM
**1**	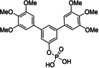	10.9±1.8 μM^[a]^	37±5 % inhibition^[a]^
**6 a**	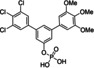	10.2±0.4 μM^[b]^	23.1±1.3 μM^[b]^
**6 b**	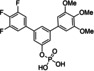	10.9±0.2 μM	29.2±1.7 μM
**6 c**	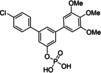	22.6±1.6 μM	46.1±0.8 μM
**6 d**	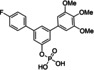	33.0±2.5 μM	51±4 % inhibition
**6 e**	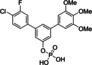	17.1±2.2 μM	51.2±4.7 μM
**6 f**	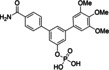	34.1±2.4 μM	21±6 % inhibition
**6 g**	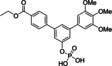	45.2±2.7 μM	61.3±1.8 μM
**6 h**	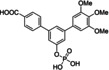	18.2±1.1 μM	50±2 % inhibition
**6 i**	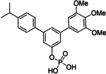	24.0±2.4 μM	25.5±2.5 μM
**6 j**	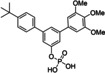	33.2±2.4 μM	25.3±2.4 μM

[a] Data taken from the literature.^[6]^ [b] n=2.

**Figure 2:**
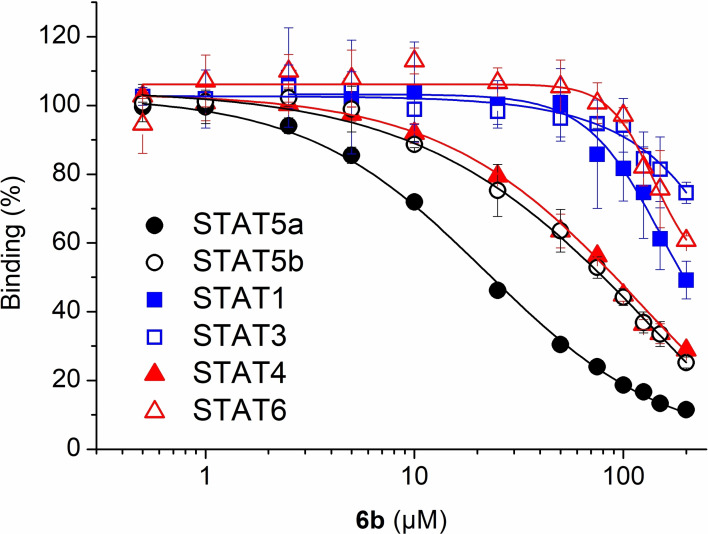
Activity of **6 b** against STAT proteins in FP assays.

Compound **6 c** containing a single chlorine substituent in the 4‐position of the variable ring was approximately half as active against STAT5a [K_i_ (STAT5a)=22.6±1.6 μM] as the triple chlorine substituted compound **6 a**, with approximately the same degree of selectivity against STAT5b [**6 c**: K_i_ (STAT5b)=46.1±0.8 μM]. A single fluorine substituent at the 4‐position of the flexibly substituted phenyl ring (**6 d**) was less beneficial for STAT5a activity [K_i_ (STAT5a)=33.0±2.5 μM] than a single chlorine substituent (**6 c**). Double substitution with chlorine and fluorine (**6 e**) in the 4‐ and 3‐position was slightly better [K_i_ (STAT5a)=17.1±2.2 μM] than the 4‐chloro substitution (**6 c**) [K_i_ (STAT5a)=22.6±1.6 μM].

Methoxy groups can act as weak hydrogen bond acceptors.^[16]^ To explore other potential hydrogen bond acceptors, we introduced a carboxamide group (**6 f**) and an ethyl ester group (**6 g**) in the 4‐position of the variable aryl ring. The carboxamide‐substituted compound **6 f** showed approximately the same activity (K_i_=34.1±2.4 μM) as the fluorine‐substituted compound **6 d** (K_i_=33.0±2.5 μM) against STAT5a. The ethyl ester **6 g** was less active against STAT5a (K_i_=45.2±2.7 μM). However, the corresponding acid **6 h** was more potent (K_i_=18.2±1.1 μM) than the ester **6 g**, and showed decent selectivity over STAT5b (50±2 % inhibition at 200 μM), suggesting that polar or even negatively charged groups on the flexibly substituted phenyl ring might be required for selective STAT5a binding. This notion was supported by the activity of alkyl‐substituted compounds **6 i** and **6 j**. While the 4‐isopropyl substituted compound **6 i** (K_i_=24.0±2.4 μM) showed approximately the same activity against STAT5a as the 4‐chloro‐substituted compound **6 c** (K_i_=22.6±1.6 μM), selectivity of **6 i** over STAT5b was completely lost [K_i_ (STAT5a)=25.5±2.5 μM]. In case of the *tert*‐butyl substituted compound **6 j**, selectivity was even slightly inverted [K_i_ (STAT5a)=33.2±2.4 μM; K_i_ (STAT5b)=25.3±2.4 μM)].

Phosphates are susceptible to hydrolytic cleavage by phosphatases in the cellular environment. Substitution of the phosphates’ bridging oxygen by methylene, monofluoromethylene, and difluoromethylene groups generates bioisosteric phosphonates, which cannot be cleaved by phosphatases.^[18]^ Although in general difluoromethylene phosphonates or methylene phosphonates are the most potent phosphonates, the monofluoromethylene phosphonate based on Stafia‐1 (Figure S1) was more potent than both the difluoromethylene phosphonate and the methylene phosphonate, despite the presence of an uninduced chiral center.^[6]^ In order to extend the synthetic methodology of asymmetrically substituted *m*‐terphenyl phosphates to phosphatase‐stable derivatives, we synthesized the monofluoromethylene phosphonate based on **6 b** as the most potent and selective terphenyl phosphate of this series. To this end, 3‐bromo‐5‐iodobenzaldehyde (**7**) was reacted with diethylphosphite to afford the α‐hydroxymethylene phosphonate **8** (Scheme 2). Nucleophilic fluorination with (diethylamino)sulfur trifluoride afforded the monofluoromethylene phosphonate **9**. Suzuki coupling with (3,4,5‐trifluorophenyl)boronic acid catalyzed by Pd(PPh_3_)_4_ proceeded selectively at the iodo‐substituted carbon atom to provide **10**. Subsequent Suzuki coupling with (3,4,5‐trimethoxyphenyl)boronic acid at the remaining, bromo‐substituted carbon atom yielded the terphenyl phosphonate **11**, which was deprotected with trimethylsilyl bromide to give the free α‐fluorobenzyl phosphonate **12**.

**Scheme 2:**
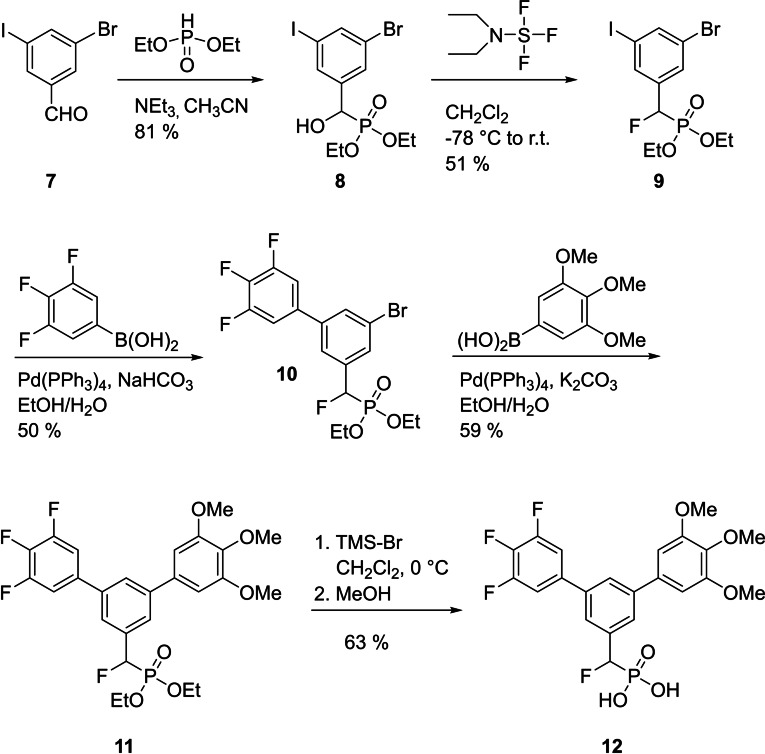
Synthesis of α‐fluorobenzyl phosphonate **12**.

Replacement of the bridging oxygen of the phosphate **6 b** [K_i_ (STAT5a)=10.9±0.2 μM] by the monofluoromethylene functionality contained in **12** was associated with only a minor degree of activity loss against STAT5a (K_i_=20.0±2.1 μM, Tables 2 and S2), resulting in improved activity of **12** against STAT5a compared to the Stafia‐1‐based monofluoromethylene phosphonate (K_i_=27.8±2.6 μM, Figure S1).^[6]^ Replacement of the bridging oxygen by monofluoromethylene was tolerated even better for STAT5b, since the monofluoromethylene phosphonate **12** is slightly more active against STAT5b (K_i_=24.9±3.3 μM) than the phosphate **6 b** [K_i_ (STAT5b)=29.2±1.7 μM]. This structural replacement was also well tolerated by STAT1, STAT3, STAT4, and STAT6, given that the activity of phosphonate **12** against these targets was only slightly decreased (in case of STAT4) or even increased (in case of STAT1, STAT3, and STAT6) as compared to the phosphate **6 b** (Tables 2 and S2). In consequence, **12** retains selectivity for STAT5a/b over other STAT family proteins (Tables 2 and S2).

**Table 2:** Selectivity profiles of phosphate **6 b** and the α‐fluorobenzyl phosphonate **12** against STAT proteins. K_i_ values were calculated from IC_50_ values (n=3) using the published equation.^[17]^

No.	Structure	STAT1 K_i_ [μM] or inhibition [%] at 200 μM	STAT3 K_i_ [μM] or inhibition [%] at 200 μM	STAT4 K_i_ [μM] or inhibition [%] at 200 μM	STAT5a K_i_ [μM] or inhibition [%] at 200 μM	STAT5b K_i_ [μM] or inhibition [%] at 200 μM	STAT6 K_i_ [μM] or inhibition [%] at 200 μM
**6 b**	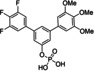	51±5 % inhibition	25±3 % inhibition	39.7±3.0 μM	10.9±0.2 μM	29.2±1.7 μM	39±1 % inhibition
**12**	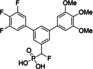	44.6±0.1 μM	71.7±3.4 μM	49.2±4.0 μM	20.0±2.1 μM	24.9±3.3 μM	71.5±1.4 μM

In summary, we have developed and implemented synthetic methodology for the synthesis of asymmetrically substituted *m*‐terphenyl phosphates and the corresponding α‐fluorobenzyl phosphonates. The data presented here provide first insights into the structure‐activity relationships of *m*‐terphenyl phosphates for use as selective STAT5a inhibitors, and serve as inspiration for further studies.

## Conflict of interest

The authors declare no conflict of interest.

## Supporting information

As a service to our authors and readers, this journal provides supporting information supplied by the authors. Such materials are peer reviewed and may be re‐organized for online delivery, but are not copy‐edited or typeset. Technical support issues arising from supporting information (other than missing files) should be addressed to the authors.

Supporting Information

## Data Availability

The data that support the findings of this study are available from the corresponding author upon reasonable request.
