# Supplementary material for: Asymmetrically Substituted m‐Terphenyl Phosphates Inhibit the Transcription Factor STAT5a
Source: Chembiochem. 2021 Dec 29;23(4):e202100603. doi: 10.1002/cbic.202100603 (PMC9303812; doi:10.1002/cbic.202100603)
Supplement: Supplementary file 1 — Supporting Information [file CBIC-23-0-s001.pdf]

ChemBioChem

Supporting Information

Asymmetrically Substituted *m*-Terphenyl Phosphates Inhibit the Transcription Factor STAT5a

Daniel Müller-Klieser and Thorsten Berg*

Table of Contents

Figure S1 ... 2

Table S1 ... 2

Table S2 ... 3

Fluorescence polarization assays ... 3

General synthetic methods ... 4

Synthesis and spectroscopic characterization of compounds ... 5

NMR spectra ... 25

Supporting references ... 43

Figure S1

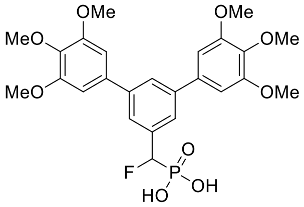

K_i (STAT5a) = 27.8 ± 2.6 µM

Figure S1: Structure of the monofluoromethylene phosphonate based on Stafia-1 and its activity against STAT5a in a fluorescence polarization assay as published previously.[1]

Table S1

No.	Structure	STAT5a IC_{50} (µM)	STAT5a K_i (µM)	STAT5b IC_{50} (µM) or inhibition (%) at 200 µM	STAT5b K_i (µM)
**1**	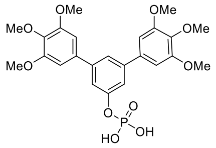	22.2 ± 3.6 µM[a]	10.9 ± 1.8 µM[a]	37 ± 5 % inhibition[a]	n/a
**6a**	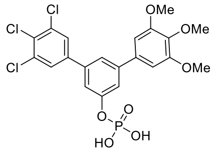	21.8 ± 0.9 µM (n=2)	10.2 ± 0.4 µM (n=2)	47.0 ± 2.6 µM (n=2)	23.1 ± 1.3 µM (n=2)
**6b**	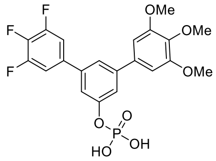	22.1 ± 0.4 µM	10.9 ± 0.2 µM	59.4 ± 3.4 µM	29.2 ± 1.7 µM
**6c**	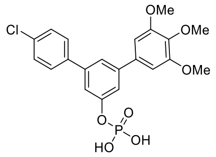	45.7 ± 3.3 µM	22.6 ± 1.6 µM	93.8 ± 1.5 µM	46.1 ± 0.8 µM
**6d**	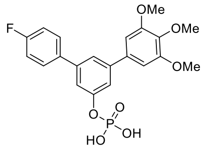	66.5 ± 5.0 µM	33.0 ± 2.5 µM	51 ± 4 % inhibition	n/a
**6e**	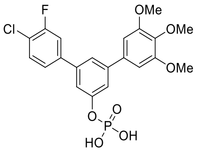	34.7 ± 4.4 µM	17.1 ± 2.2 µM	104.1 ± 9.5 µM	51.2 ± 4.7 µM

No.	Structure	STAT5a IC_{50} (µM)	STAT5a K_i (µM)	STAT5b IC_{50} (µM) or inhibition (%) at 200 µM	STAT5b K_i (µM)
**6f**	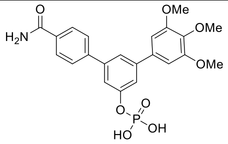	68.9 ± 4.9 µM	34.1 ± 2.4 µM	21 ± 6 % inhibition	n/a
**6g**	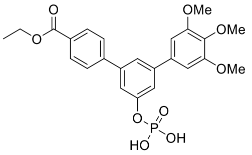	91.2 ± 5.5 µM	45.2 ± 2.7 µM	124.6 ± 3.7 µM	61.3 ± 1.8 µM
**6h**	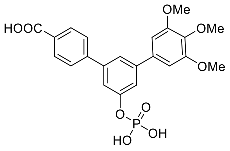	36.8 ± 2.2 µM	18.2 ± 1.1 µM	50 ± 2 % inhibition	n/a
**6i**	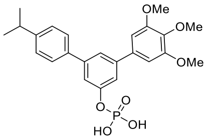	48.5 ± 4.8 µM	24.0 ± 2.4 µM	51.8 ± 5.1 µM	25.5 ± 2.5 µM
**6j**	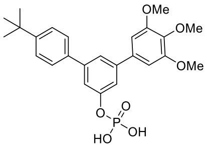	66.9 ± 4.9 µM	33.2 ± 2.4 µM	51.4 ± 4.9 µM	25.3 ± 2.4 µM

Table S1: Activities of test compounds against STAT5a and STAT5b in FP assays. Experiments were carried out in triplicate (n = 3) unless stated otherwise. n/a: not applicable.
[a]data taken from the literature.[1]
K_i values were calculated from IC_{50} values using the published equation.[2]

Table S2

No	Structure	STAT1 IC_{50} (µM)	STAT3 IC_{50} (µM)	STAT4 IC_{50} (µM)	STAT5a IC_{50} (µM)	STAT5b IC_{50} (µM)	STAT6 IC_{50} (µM)
**12**	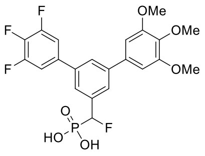	90.6 ± 0.2	144.8 ± 6.8	99.3 ± 8.0	40.4 ± 4.3	50.7 ± 6.8	144.3 ± 2.8

Table S2: Activities of **12** against STAT5 proteins in FP assays.

Fluorescence polarization assays

Fluorescence polarization assays were essentially carried out as previously described.[1] These assays quantitate to which extent a test compound is able to interfere with binding between a STAT protein and a fluorescein-labeled phosphotyrosine-containing peptide, which is known to bind to the SH2 domain of the respective STAT protein. The following protein

concentrations were used: 100 nM for STAT1, 150 nM for STAT3, 30 nM for STAT4, 180 nM for STAT5a, 90 nM for STAT5b and 105 nM for STAT6. All peptides were used at a concentration of 10 nM. The following peptides were used: 5-carboxyfluorescein-GpYDKPHVL for STAT1; 5-carboxyfluorescein-GpYLPQTV-NH_2 for STAT3; 5-carboxyfluorescein-GpYLPQNID-OH for STAT4; 5-carboxyfluorescein-GpYLVLDKW for STAT5a/b, and 5-carboxyfluorescein-GpYVPWQDLI-OH for STAT6. As a buffer system, 10 mM Tris (pH 8.0), 50 mM NaCl, 1 mM EDTA, 1 mM DTT, 0.1 % Nonidet P-40 substitute, and 2 % DMSO in water was used. All compounds tested were incubated for 1 h with the protein. Subsequently, the fluorescein-labeled peptide was added and the fluorescence polarization was measured with an Infinite F500 plate reader (Tecan) after another hour. IC_{50} values were converted to K_i-values using the published equation.[2]

General synthetic methods

Method 1: Suzuki coupling

A Schlenk flask was charged with 80 mg of aryl bromide **3** (236 µmol, 1 eq.), the corresponding boronic acid (472 µmol, 2 eq.), 82 mg of K_2CO_3 (590 µmol, 2.5 eq.) and 5 ml of a water/ethanol 3:2 solution was added. The solution was degassed by applying ultrasonic under vacuum for one minute four times. 14 mg of $Pd(PPh_3)_4$ (12 µmol, 5 mol %) were added and the solution was refluxed overnight. After cooling down, the mixture was extracted with ethyl acetate, washed with brine and dried over Na_2SO_4. The crude product was purified by flash column chromatography.

Method 2: Synthesis of dibenzylphosphate esters

The corresponding phenol (1 eq.) was dissolved in 10 ml/mmol MeCN or DMF and CCl_4 (5 eq.), DIPEA (2 eq.) and DMAP (0.1 eq.) were added. After cooling down to 0°C, dibenzyl phosphite (1.5 eq.) was added dropwise and stirring was continued at the same temperature. After completion of the reaction (mostly 30-60 minutes) it was quenched by adding a 0.5 M KH_2PO_4 solution. The mixture was extracted with ethyl acetate, washed with brine and dried over Na_2SO_4. The crude product was purified by flash column chromatography.

Method 3: Debenzylation by hydrogenolysis

The corresponding benzyl protected phosphate was dissolved in 20 ml/mmol of an ethanol/ethyl acetate 2:1 mixture. Then, a few mg of 10 % Pd/C were added and the mixture was stirred under a hydrogen atmosphere. Upon completion of the reaction, the mixture was filtered through celite and cotton. In case the purity of the product was not sufficient, purification was done by reversed-phase column chromatography.

Synthesis and spectroscopic characterization of compounds

5-Bromo-3',4',5'-trimethoxy-[1,1'-biphenyl]-3-ol (**3**)

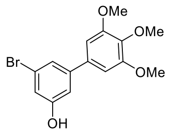

To a degassed solution of 800 mg (2.68 mmol; 1 eq.) of 3-bromo-5-iodophenol, 681 mg (3.21 mmol; 1.2 eq.) of trimethoxy phenylboronic acid, 742 mg (8.83 mmol; 3.3 eq.) of $NaHCO_3$ in 26 ml of ethanol and 13 ml of water was added 155 mg (134 µmol, 0.05 eq.) of $Pd(PPh_3)_4$ and the mixture was refluxed for 16 h. After extraction with ethyl acetate the solution was dried over $MgSO_4$. The crude product was purified by flash column chromatography (hexane/ethyl acetate 5:1 → 2:1) and 729 mg (80 %) of a colorless solid were obtained.
R_f = 0.45 (hexane/ethyl acetate 2:1); Melting point = 61°C; ^{1}H NMR (400 MHz, Chloroform-*d*) δ 7.25 (dd, *J* = 1.6, 1.6 Hz, 1H), 7.00 (dd, *J* = 2.3, 1.6 Hz, 1H), 6.93 (dd, *J* = 2.3, 1.6 Hz, 1H), 6.70 (s, 2H), 3.90 (s, 6H), 3.89 (s, 3H) ppm; ^{13}C NMR (101 MHz, $CDCl_3$) δ 156.85, 153.56, 144.55, 138.01, 135.73, 123.14, 122.66, 117.68, 113.35, 104.49, 61.16, 56.43 ppm; IR (KBr): $\tilde{\nu}$ = 3447, 3064, 3006, 2976, 2940, 2909, 2835, 1955, 1710, 1608, 1583, 1513, 1488, 1461, 1431, 1415, 1393, 1331, 1314, 1277, 1239, 1179, 1165, 1127, 1081, 1037, 1009, 987, 961, 878, 855, 829, 818, 783, 758, 727, 697, 671, 651, 542, 531, 495, 449 cm^{-1}; UV/Vis: λ (nm) = 268, 229; HRMS (ESI) found: [M-H$^+$] m/z = 337.0077, calcd. for $C_{15}H_{14}BrO_4$: 337.0081.

3,4,5-Trichloro-3'',4'',5''-trimethoxy-[1,1':3',1''-terphenyl]-5'-ol (**4a**)

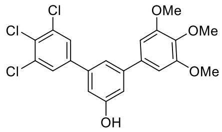

Compound **4a** was synthesized from compound **3** according to Method 1. The crude product was purified by flash column chromatography (hexane/ethyl acetate 6:1 → 3:1) and 93 mg (90 %) of a colorless solid were obtained.
R_f = 0.50 (hexane/ethyl acetate 3:2); Melting point = 214 °C; ^{1}H NMR (400 MHz, Chloroform-*d*) δ 7.62 (s, 2H), 7.22 (dd, *J* = 1.6, 1.6 Hz, 1H), 7.07 – 7.03 (m, 1H), 6.99 – 6.94 (m, 1H), 6.76 (s, 2H), 5.15 (s, 1H), 3.94 (s, 6H), 3.91 (s, 3H) ppm; ^{13}C NMR (101 MHz, $CDCl_3$) δ 156.59, 153.69, 144.31, 140.91, 140.01, 138.23, 136.42, 134.66, 130.74, 127.45, 118.59, 114.59, 113.00, 104.72, 61.15, 56.46 ppm; IR (KBr): $\tilde{\nu}$ = 3392, 3066, 2998, 2936, 2838, 1736, 1704, 1583, 1543, 1512, 1492, 1465, 1431, 1406, 1366, 1301, 1240, 1186, 1165, 1127, 1037, 1001, 939, 873, 852, 832, 807, 764, 742, 700, 684, 670, 639, 556, 505 cm^{-1}; UV/Vis: λ (nm) = 265, 229, 215; HRMS (ESI) found: [M+H$^+$] m/z = 439.0274, calcd. for $C_{21}H_{18}Cl_3O_4$: 439.0265.

Dibenzyl 3,4,5-trichloro-3",4",5"-trimethoxy-[1,1':3',1''-terphenyl]-5'-yl) phosphate (**5a**)

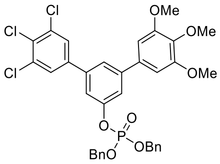

Compound **5a** was synthesized from compound **4a** (93 mg, 212 µmol) according to Method 2. The crude product was purified by flash column chromatography (hexane/ethyl acetate 6:1 → 3:1) yielding 65 mg (44 %) of a colorless oil.

R_f = 0.55 (hexane/ethyl acetate 3:2); ^{1}H NMR (400 MHz, Chloroform-*d*) δ 7.52 (s, 2H), 7.45 – 7.39 (m, 1H), 7.38 – 7.27 (m, 11H), 7.17 – 7.11 (m, 1H), 6.70 (s, 2H), 5.24 – 5.12 (m, 4H), 3.91 (s, 6H), 3.90 (s, 3H) ppm; ^{13}C NMR (76 MHz, $CDCl_3$) δ 153.73, 151.45 (d, *J* = 6.9 Hz), 144.32, 140.13, 139.93, 138.51, 135.60, 135.40 (d, *J* = 6.5 Hz), 134.72, 131.05, 128.97, 128.81, 128.25, 127.44, 122.58, 119.25 (d, *J* = 5.0 Hz), 117.56 (d, *J* = 4.8 Hz), 104.79, 70.38 (d, *J* = 5.9 Hz), 61.14, 56.49 ppm; ^{31}P NMR (162 MHz, $CDCl_3$) δ -6.15 ppm; IR (Film): $\tilde{\nu}$ = 3686, 3667, 3646, 3443, 2997, 2961, 2834, 1669, 1653, 1647, 1636, 1578, 1495, 1463, 1431, 1397, 1362, 1300, 1237, 1185, 1128, 1004, 827, 699, 518 cm^{-1}; UV/Vis: λ (nm) = 264, 232, 217; HRMS (ESI) found: [M+H^{+}] m/z = 699.0850, calcd. for $C_{35}H_{31}Cl_3O_7P$: 699.0868.

(3,4,5-Trichloro-3",4",5"-trimethoxy-[1,1':3',1''-terphenyl]-5'-yl) dihydrogen phosphate (**6a**)

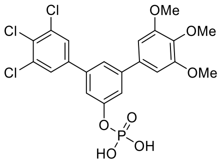

Compound **6a** was synthesized from compound **5a** (53 mg, 76 µmol) according to Method 3. The crude product was purified by reversed-phase column chromatography (water/acetonitrile 3:1) yielding 27 mg (70 %) of a colorless solid.

R_f (RP) = 0.20 (water/acetonitrile 4:1); Melting point = 154 °C; ^{1}H NMR (400 MHz, Methanol-*d*$_4$) δ 7.91 (s, 2H), 7.57 – 7.50 (m, 2H), 7.48 – 7.42 (m, 1H), 6.98 (s, 2H), 3.93 (s, 6H), 3.81 (s, 3H) ppm; ^{13}C NMR (101 MHz, MeOD) δ 156.41, 154.77, 144.44, 143.13, 140.03, 138.96, 138.23, 135.33, 130.77, 128.72, 120.61 (d, *J* = 5.3 Hz), 120.45, 119.00 (d, *J* = 4.8 Hz), 105.87, 61.19, 56.81 ppm; ^{31}P NMR (162 MHz, MeOD) δ -3.04 ppm; IR (KBr): $\tilde{\nu}$ = 3435, 2936, 2839, 2359, 2343, 1642, 1583, 1544, 1511, 1466, 1428, 1403, 1384, 1365, 1298, 1241, 1216, 1186, 1165, 1126, 1037, 1005, 993, 941, 924, 861, 834, 807, 781, 699, 684, 669, 550, 530, 520 cm^{-1}; UV/Vis: λ (nm) = 263, 216; HRMS (ESI) found: [M-H^{+}] m/z = 516.9777, calcd. for $C_{21}H_{17}Cl_3O_7P$: 516.9783.

3,4,5-Trifluoro-3'',4'',5''-trimethoxy-[1,1':3',1''-terphenyl]-5'-ol (**4b**)

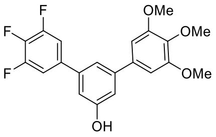

Compound **4b** was synthesized from compound **3** according to Method 1. The crude product was purified by flash column chromatography (hexane/ethyl acetate 6:1 → 2:1) and 88 mg (96 %) of a colorless solid were obtained.

R_f = 0.65 (dichloromethane/acetone 95:5); Melting point = 192°C; ^{1}H NMR (400 MHz, Chloroform-*d*) δ 7.24 – 7.16 (m, 3H), 7.05 – 7.02 (m, 1H), 6.95 – 6.91 (m, 1H), 6.77 (s, 2H), 5.25 (s, 1H), 3.93 (s, 6H), 3.91 (s, 3H) ppm; ^{13}C NMR (75 MHz, $CDCl_3$) δ 156.62, 153.67, 144.17, 140.48, 138.13, 136.50, 118.47, 114.36, 112.99, 111.55 – 111.10 (m), 104.66, 61.16, 56.42 ppm; ^{19}F NMR (377 MHz, Chloroform-*d*) δ -133.77 – -134.07 (m), -161.96 (tt, *J* = 20.5, 6.5 Hz) ppm; IR (KBr): $\tilde{\nu}$ = 3677, 3650, 3628, 3420, 3004, 2942, 2836, 2361, 1586, 1531, 1491, 1465, 1453, 1420, 1396, 1341, 1279, 1240, 1188, 1165, 1128, 1092, 1069, 1042, 998, 967, 868, 849, 835, 786, 755, 729, 706, 668, 651, 521 cm^{-1}; UV/Vis: λ (nm) = 257, 233; HRMS (ESI) found: [$M+Na^+$] m/z = 413.0969, calcd. for $C_{21}H_{17}F_3NaO_4$: 413.0971.

Dibenzyl (3,4,5-trifluoro-3”,4”,5”-trimethoxy-[1,1':3',1''-terphenyl]-5'-yl) phosphate (**5b**)

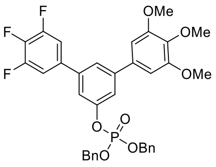

Compound **5b** was synthesized from compound **4b** (46 mg, 118 µmol) according to Method 2. The crude product was purified by flash column chromatography (hexane/ethyl acetate 6:1 → 2:1) and 29 mg (38 %) of a colorless oil were obtained.

R_f = 0.20 (hexane/ethyl acetate 4:1); ^{1}H NMR (400 MHz, Chloroform-*d*) δ 7.41 – 7.39 (m, 1H), 7.37 – 7.31 (m, 10H), 7.30 – 7.28 (m, 1H), 7.18 – 7.12 (m, 1H), 7.12 – 7.07 (m, 2H), 6.70 (s, 2H), 5.22 – 5.11 (m, 4H), 3.91 (s, 6H), 3.90 (s, 3H) ppm; ^{13}C NMR (101 MHz, $CDCl_3$) δ 153.75, 151.49 (d, *J* = 7.3 Hz), 144.25, 135.62, 135.47, 135.40, 128,98, 128.82, 128.25, 122.49, 119.01 (d, *J* = 5.2 Hz), 117.50 (d, *J* = 4.6 Hz), 111.60 – 111.29 (m), 104.77, 70.36 (d, *J* = 5.8 Hz), 61.14, 56.48 ppm; ^{19}F NMR (377 MHz, Chloroform-*d*) δ -133.58 – -133.72 (m), -161.48 (tt, *J* = 20.5, 6.4 Hz) ppm; ^{31}P NMR (162 MHz, $CDCl_3$) δ -6.17 ppm; IR (Film): $\tilde{\nu}$ = 3067, 3033, 3007, 2958, 2938, 2833, 1958, 1618, 1606, 1582, 1530, 1512, 1466, 1416, 1395, 1337, 1286, 1267, 1243, 1216, 1188, 1161, 1129, 1107, 1083, 1044, 1008, 993, 968, 898, 853, 832, 787, 749, 697, 666, 643, 608, 598, 539, 528, 485, 463 cm^{-1}; UV/Vis: λ (nm) = 258, 231, 215, 203 nm; HRMS (ESI) found: [$M+Na^+$] m/z = 673.1551, calcd. for $C_{35}H_{30}F_3NaO_7P$: 673.1573.

(3,4,5-Trifluoro-3”,4”,5”-trimethoxy-[1,1':3',1''-terphenyl]-5'-yl) dihydrogen phosphate (**6b**)

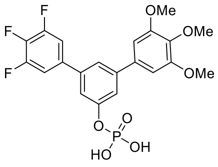

Compound **6b** was synthesized from compound **5b** (29 mg, 45 µmol) according to Method 3. Thereby 20 mg (93 %) of a colorless solid were obtained.

R_f (RP) = 0.65 (water/acetonitrile 1:1); Melting point >260 °C; ^{1}H NMR (400 MHz, Methanol-*d*$_4$) δ 7.57 – 7.45 (m, 5H), 6.97 (s, 2H), 3.93 (s, 6H), 3.81 (s, 3H) ppm; ^{13}C NMR (101 MHz, CD_3OD) δ 155.62 (d, *J* = 5.8 Hz), 154.81, 152.50 (ddd, *J* = 247.2, 10.2, 4.2 Hz), 144.54, 140.76, 139.04, 138.02, 121.14, 120.30 (d, *J* = 4.4 Hz), 118.98 (d, *J* = 4.1 Hz), 112.64 – 112.34 (m), 105.84, 61.19, 56.80 ppm; ^{19}F NMR (377 MHz, Methanol-*d*$_4$) δ -137.90 (dd, *J* = 19.9, 9.4 Hz), -166.92 (tt, *J* = 19.9, 9.8 Hz) ppm; ^{31}P NMR (162 MHz, CD_3OD) δ -4.67 ppm; IR (KBr): $\tilde{\nu}$ = 3446, 3002, 2939, 2844, 1719, 1618, 1585, 1531, 1512, 1466, 1416, 1396, 1337, 1271, 1243, 1188, 1165, 1127, 1044, 1007, 963, 940, 854, 834, 787, 759, 707, 667, 648, 607, 531 cm^{-1}; UV/Vis: λ (nm) = 257, 208 nm; HRMS (ESI) found: [M-H$^+$] m/z = 469.0657, calcd. for $C_{21}H_{17}F_3O_7P$: 469.0669.

4''-Chloro-3,4,5-trimethoxy-[1,1':3',1''-terphenyl]-5'-ol (**4c**)

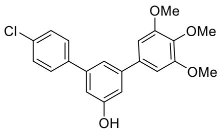

Compound **4c** was synthesized from compound **3** according to Method 1. The crude product was purified by flash column chromatography (hexane/ethyl acetate 9:1 → 3:1) and 85 mg (97 %) of a colorless solid were obtained.

R_f = 0.40 (hexane/ethyl acetate 3:2); Melting point = 156 °C; ^{1}H NMR (400 MHz, Chloroform-*d*) δ 7.56 – 7.50 (m, 2H), 7.45 – 7.38 (m, 2H), 7.29 – 7.24 (m, 1H), 7.00 (ddd, *J* = 7.4, 2.2, 2.2 Hz, 2H), 6.78 (s, 2H), 5.30 (s, 1H), 3.92 (s, 6H), 3.90 (s, 3H) ppm; ^{13}C NMR (101 MHz, $CDCl_3$) δ 156.54, 153.60, 143.85, 142.29, 139.34, 137.97, 136.87, 133.90, 129.11, 128.59, 118.62, 113.59, 113.12, 104.66, 61.14, 56.39 ppm; IR (KBr): $\tilde{\nu}$ = 3374, 2998, 2971, 2936, 2905, 2832, 1738, 1702, 1584, 1513, 1481, 1464, 1452, 1433, 1408, 1388, 1369, 1304, 1240, 1208, 1186, 1165, 1128, 1092, 1071, 1036, 1012, 996, 922, 883, 872, 827, 788, 751, 726, 703, 679, 668, 637, 546, 528, 486, 477 cm^{-1}; UV/Vis: λ (nm) = 261, 231; HRMS (ESI) found: [M+H$^+$] m/z = 371.1046, calcd. for $C_{21}H_{20}ClO_4$: 371.1045.

Dibenzyl (4''-chloro-3,4,5-trimethoxy-[1,1':3',1''-terphenyl]-5'-yl) phosphate (**5c**)

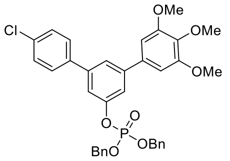

Compound **5c** was synthesized from compound **4c** (54 mg, 146 µmol) according to Method 2. The crude product was purified by flash column chromatography (hexane/ethyl acetate 9:1 → 3:1) yielding 62 mg (67 %) of a colorless oil.

R_f = 0.45 (hexane/ethyl acetate 3:2); ^{1}H NMR (400 MHz, Chloroform-*d*) δ 7.50 – 7.46 (m, 1H), 7.46 – 7.38 (m, 4H), 7.38 – 7.30 (m, 11H), 7.29 – 7.24 (m, 2H), 6.72 (s, 2H), 5.28 – 5.04 (m, 4H), 3.90 (s, 9H) ppm; ^{13}C NMR (101 MHz, $CDCl_3$) δ 153.68, 151.39 (d, *J* = 7.0 Hz), 143.94, 142.25, 138.57, 138.35, 136.02, 135.52 (d, *J* = 6.8 Hz), 134.21, 129.16, 128.88, 128.78, 128.64, 128.22, 122.68, 118.18 (d, *J* = 5.1 Hz), 117.60 (d, *J* = 4.5 Hz), 104.76, 70.26 (d, *J* = 5.9 Hz), 61.14, 56.45 ppm; ^{31}P NMR (162 MHz, $CDCl_3$) δ -6.10 ppm; IR (Film): $\tilde{\nu}$ = 3088, 3066, 3034, 3000, 2936, 2897, 2830, 2593, 1958, 1902, 1652, 1604, 1582, 1513, 1463, 1456, 1431, 1406, 1385, 1357, 1299, 1238, 1213, 1180, 1164, 1127, 1108, 1092, 1042, 965, 919, 879, 848, 825, 784, 742, 697, 679, 666, 638, 621, 600, 547, 507, 499, 475 cm^{-1}; UV/Vis: λ (nm) = 263, 230, 215; HRMS (ESI) found: [$M+Na^+$] m/z = 653.1463, calcd. for $C_{35}H_{32}ClNaO_7P$: 653.1466.

(4''-Chloro-3,4,5-trimethoxy-[1,1':3',1''-terphenyl]-5'-yl) dihydrogen phosphate (**6c**)

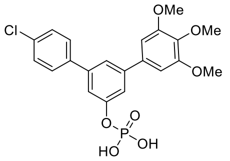

Compound **6c** was synthesized from compound **5c** (30 mg, 48 µmol) according to Method 3. Thereby 14 mg (65 %) of a colorless solid were obtained.

R_f (RP) = 0.65 (water/acetonitrile 2:1); Melting point = 129 °C; ^{1}H NMR (400 MHz, Methanol-*d*$_4$) δ 7.92 – 7.22 (m, 7H), 6.93 (s, 2H), 3.91 (s, 6H), 3.80 (s, 3H) ppm; ^{13}C NMR (75 MHz, CD_3OD) δ 154.80, 144.47, 139.05, 134.75, 129.93, 129.73, 128.72, 128.19, 122.25, 119.11, 118.91, 105.74, 61.19, 56.77; ^{31}P NMR (162 MHz, CD_3OD) δ -4.91 ppm; IR (KBr): $\tilde{\nu}$ = 3444, 2935, 2849, 1646, 1636, 1583, 1509, 1499, 1465, 1431, 1424, 1398, 1386, 1359, 1297, 1242, 1181, 1163, 1127, 1092, 1073, 1037, 1006, 991, 952, 903, 828, 765, 703, 627, 550, 508, 413 cm^{-1}; UV/Vis: λ (nm) = 261, 230, 215; HRMS (ESI) found: [$M-H^+$] m/z = 449.0538, calcd. for $C_{21}H_{19}ClO_7P$: 449.0562.

4''-Fluoro-3,4,5-trimethoxy-[1,1':3',1''-terphenyl]-5'-ol (**4d**)

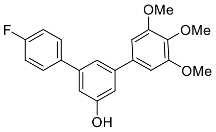

Compound **4d** was synthesized from compound **3** according to Method 1. The crude product was purified by flash column chromatography (hexane/ethyl acetate 6:1 → 2:1) and 79 mg (95 %) of a colorless oil were obtained.

R_f = 0.40 (hexane/ethyl acetate 3:2); Melting point = 143° C; ^{1}H NMR (300 MHz, Chloroform-*d*) δ 7.61 – 7.52 (m, 2H), 7.27 – 7.26 (m, 1H), 7.18 – 7.09 (m, 2H), 7.02 – 6.96 (m, 2H), 6.79 (s, 2H), 3.93 (s, 6H), 3.91 (s, 3H) ppm; ^{13}C NMR (75 MHz, $CDCl_3$) δ 162.81 (d, *J* = 246.9 Hz), 156.37, 153.62, 153.56, 143.83, 142.59, 138.04, 136.99 (d, *J* = 3.3 Hz), 136.86, 128.94 (d, *J* = 8.1 Hz), 118.80, 115.85 (d, *J* = 21.5 Hz), 113.25, 113.16, 104.68, 61.14, 56.40 ppm; ^{19}F NMR (377 MHz, $CDCl_3$) δ -115.00 ppm; IR (KBr): $\tilde{\nu}$ = 3409, 3068, 3052, 3002, 2937, 2839, 1602, 1584, 1537, 1514, 1485, 1463, 1434, 1411, 1391, 1368, 1304, 1271, 1239, 1187, 1162, 1128, 1071, 1037, 998, 980, 885, 830, 812, 781, 723, 701, 667, 608, 567, 552, 528, 499, 419 cm^{-1}; UV/Vis: λ (nm) = 257, 230, 214; HRMS (ESI) found: $[M+H^+]$ m/z = 355.1339, calcd. for $C_{21}H_{20}FO_4$: 355.1340.

Dibenzyl (4''-fluoro-3,4,5-trimethoxy-[1,1':3',1''-terphenyl]-5'-yl) phosphate (**5d**)

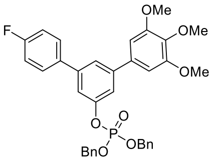

Compound **5d** was synthesized from compound **4d** (29 mg, 82 µmol) according to Method 2. The crude product was purified by flash column chromatography (hexane/ethyl acetate 9:1 → 3:1) and 23 mg (75 %) of a colorless oil were obtained.

R_f = 0.45 (hexane/ethyl acetate 3:2); ^{1}H NMR (400 MHz, Chloroform-*d*) δ 7.52 – 7.43 (m, 3H), 7.38 – 7.29 (m, 10H), 7.28 – 7.25 (m, 2H), 7.16 – 7.08 (m, 2H), 6.72 (s, 2H), 5.24 – 5.11 (m, 4H), 3.90 (s, 9H) ppm; ^{13}C NMR (101 MHz, $cdcl_3$) δ 162.96 (d, *J* = 247.3 Hz), 153.73, 151.41 (d, *J* = 6.9 Hz), 143.87, 142.53, 138.53, 136.31 (d, *J* = 3.2 Hz), 136.08, 135.60 (d, *J* = 6.9 Hz), 129.02 (d, *J* = 8.1 Hz), 128.86, 128.77, 128.21, 122.71, 117.87 (d, *J* = 5.0 Hz), 117.66 (d, *J* = 4.6 Hz), 115.90 (d, *J* = 21.5 Hz), 104.97, 70.26 (d, *J* = 5.8 Hz), 61.12, 56.51 ppm; ^{31}P NMR (162 MHz, $CDCl_3$) δ -6.11 ppm; IR (Film): $\tilde{\nu}$ = 3066, 3034, 2998, 2963, 2939, 2897, 2831, 1702, 1605, 1581, 1514, 1463, 1457, 1432, 1409, 1389, 1358, 1297, 1239, 1224, 1179, 1162, 1127, 1105, 1034, 988, 918, 883, 856, 832, 780, 740, 698, 667, 621, 599, 565, 547, 510, 503, 488

cm^{-1}; UV/Vis: λ (nm) = 258, 233; HRMS (ESI) found: [M+Na$^+$] m/z = 637.1750, calcd. for $C_{35}H_{32}FNaO_7P$: 637.1762.

(4''-Fluoro-3,4,5-trimethoxy-[1,1':3',1''-terphenyl]-5'-yl) dihydrogen phosphate (**6d**)

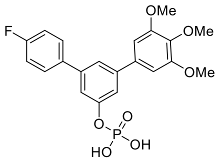

Compound **6d** was synthesized from compound **5d** (29 mg, 47 µmol) according to Method 3. Thereby 15 mg (72 %) of a colorless solid were obtained.

R_f (RP) = 0.70 (water/acetonitrile 2:1); Melting point = 165 °C; ^{1}H NMR (400 MHz, Methanol-*d*$_4$) δ 7.76 – 7.66 (m, 2H), 7.53 – 7.37 (m, 3H), 7.17 (dd, *J* = 8.5, 8.5 Hz, 2H), 6.95 (s, 2H), 3.92 (s, 6H), 3.80 (s, 3H) ppm; ^{13}C NMR (101 MHz, CD_3OD) δ 164.00 (d, *J* = 245.3 Hz), 155.54, 154.76, 144.17, 142.92, 138.90, 138.53 (d, *J* = 3.0 Hz), 138.47, 130.06 (d, *J* = 8.0 Hz), 121.12, 119.30 – 118.98 (m), 116.44 (d, *J* = 21.7 Hz), 105.81, 61.20, 56.78 ppm; ^{19}F NMR (377 MHz, CD_3OD) δ -118.60 ppm; ^{31}P NMR (162 MHz, MeOD) δ -4.56 ppm; IR (KBr): $\tilde{\nu}$ = 3434, 2936, 2841, 2362, 1669, 1645, 1603, 1582, 1513, 1463, 1435, 1408, 1389, 1359, 1299, 1240, 1183, 1162, 1126, 1103, 1074, 1037, 1004, 989, 926, 903, 832, 780, 701, 668, 569, 553, 526, 515, 504, 425, 418 cm^{-1}; UV/Vis: λ (nm) = 258, 231, 210; HRMS (ESI) found: [M-H$^+$] m/z = 433.0858, calcd. for $C_{35}H_{31}FO_7P$: 433.0858.

3-Fluoro-4-chloro-3'',4'',5''-trimethoxy-[1,1':3',1''-terphenyl]-5'-ol (**4e**)

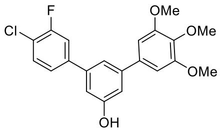

Compound **4e** was synthesized from compound **3** (100 mg, 295 µmol) according to Method 1. The crude product was purified by flash column chromatography (hexane/ethyl acetate 3:2) and 109 mg (95 %) of a colorless solid were obtained.

R_f = 0.40 (hexane/ethyl acetate 3:2); Melting point = 70 °C; ^{1}H NMR (400 MHz, Chloroform-*d*) δ 7.50 – 7.43 (m, 1H), 7.39 (dd, *J* = 10.2, 2.1 Hz, 1H), 7.35 – 7.31 (m, 1H), 7.26 – 7.24 (m, 1H), 7.03 (dd, *J* = 2.4, 1.6 Hz, 1H), 6.98 (dd, *J* = 2.4, 1.6 Hz, 1H), 6.78 (s, 2H), 3.93 (s, 6H), 3.91 (s, 3H) ppm; ^{13}C NMR (75 MHz, $CDCl_3$) δ 158.45 (d, *J* = 248.4 Hz), 156.55, 153.66, 144.06, 141.53, 141.44, 141.23 (d, *J* = 1.7 Hz), 138.12, 136.62, 131.03, 123.60 (d, *J* = 3.6 Hz), 118.60, 115.41 (d, *J* = 21.8 Hz), 114.10, 113.04, 104.68, 61.15, 56.42 ppm; IR (KBr): $\tilde{\nu}$ = 3433, 3002, 2936, 2839, 1583, 1513, 1479, 1464, 1432, 1414, 1393, 1365, 1306, 1277, 1240, 1185, 1164, 1127, 1078, 1051, 997, 953, 880, 861, 827, 779, 709, 675, 670, 640, 561, 534, 447 cm$^-$

[1]; UV (DCM): λ_{max} = 262, 230 nm; HRMS (ESI) found: [M+H$^+$] m/z = 389.0949, calcd. for $C_{21}H_{18}ClFO_4$: 389.0950.

Dibenzyl (3-fluoro-4-chloro-3”,4”,5”-trimethoxy-[1,1':3',1''-terphenyl]-5'-yl) phosphate (**5e**)

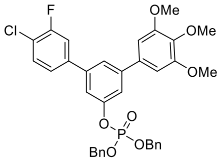

Compound **5e** was synthesized from compound **4e** (107 mg, 276 µmol) according to Method 2. The crude product was purified by flash column chromatography (hexane/ethyl acetate 4:1 → 3:1) and 70 mg (39 %) of a colorless oil were obtained.

R_f = 0.25 (hexane/ethyl acetate 3:2); ^{1}H NMR (300 MHz, Chloroform-*d*) δ 7.50 – 7.39 (m, 3H), 7.38 – 7.28 (m, 10H), 7.26 – 7.27 (m, 1H), 7.25 – 7.20 (m, 2H), 6.71 (s, 2H), 5.24 – 5.07 (m, 4H), 3.91 – 3.90 (m, 9H) ppm; ^{31}P NMR (162 MHz, Chloroform-*d*) δ - 6.14 ppm; IR (Film): $\tilde{\nu}$ = 3666, 3477, 3090, 3066, 3031, 3010, 2959, 2937, 2899, 2832, 1604, 1580, 1511, 1497, 1463, 1431, 1410, 1390, 1356, 1303, 1281, 1240, 1217, 1191, 1161, 1128, 1110, 1080, 1038, 1006, 991, 969, 957, 898, 830, 821, 753, 698, 675, 666, 646, 599, 559, 538, 486, 451 cm^{-1}; UV (DCM): λ_{max} = 264; 231 nm; HRMS (ESI) found: [M+H$^+$] m/z = 649.1532, calcd. for $C_{35}H_{31}ClFO_6P$: 649.1553.

(3-Fluoro-4-chloro-3”,4”,5”-trimethoxy-[1,1':3',1''-terphenyl]-5'-yl) dihydrogen phosphate (**6e**)

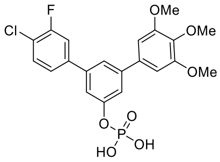

Compound **6e** was synthesized from compound **5e** (60 mg, 93 µmol) according to Method 3. Thereby 38 mg (87 %) of a colorless solid were obtained.

R_f (RP) = 0.55 (water/acetonitrile 1:1); Melting point = 127 °C; ^{1}H NMR (400 MHz, Methanol-*d*$_4$) δ 7.70 – 7.39 (m, 6H), 6.94 (s, 2H), 3.92 (s, 6H), 3.81 (s, 3H) ppm; ^{13}C NMR (101 MHz, CD_3OD) δ 159.60 (d, *J* = 247.6 Hz), 154.86, 154.40 – 154.03 (m), 144.83, 142.71 (d, *J* = 6.9 Hz), 141.90, 139.21, 137.76, 137.62, 132.07, 124.96 (d, *J* = 2.8 Hz), 122.31, 121.09 (d, *J* = 17.7 Hz), 119.85, 118.79, 116.26 (d, *J* = 22.3 Hz), 105.81, 61.19, 56.79 ppm; ^{31}P NMR (162 MHz, Methanol-*d*$_4$) δ -5.48 ppm; IR (KBr): $\tilde{\nu}$ = 3444, 2936, 2850, 2358, 2336, 1732, 1715, 1697, 1635, 1583, 1511, 1498, 1464, 1434, 1410, 1393, 1357, 1303, 1278, 1242, 1186, 1161, 1127, 1081, 1051, 1036, 1008, 993, 955, 865, 831, 789, 705, 675, 668, 604, 551, 530, 522, 507, 496, 483, 454, 442 cm^{-1}; UV (DCM): λ_{max}= 260, 207 nm; HRMS (ESI) found: [M-H$^+$] m/z = 467.0468, calcd. for $C_{21}H_{18}ClFO_7P$: 467.0451.

4''-Carbamoyl-3,4,5-trimethoxy-[1,1':3',1''-terphenyl]-5'-ol (**4f**)

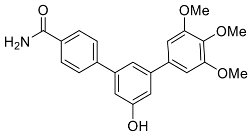

Compound **4f** was synthesized from compound **3** according to Method 1. The crude product was purified by flash column chromatography (ethyl acetate/acetone/methanol 8:1:1) and 90 mg (quant.) of a colorless solid were obtained.

R_f = 0.40 (acetone/DCM 1:1); Melting point = 207 °C; ^{1}H NMR (400 MHz, DMSO-d_6) δ 9.71 (s, 1H), 8.02 (s, 1H), 7.99 – 7.94 (m, 2H), 7.80 – 7.75 (m, 2H), 7.39 – 7.32 (m, 2H), 7.08 – 7.02 (m, 2H), 6.92 (s, 2H), 3.87 (s, 6H), 3.70 (s, 3H) ppm; ^{13}C NMR (101 MHz, DMSO-d_6) δ 167.56, 158.15, 153.14, 142.99, 142.65, 141.17, 137.25, 136.17, 133.18, 128.07, 126.66, 116.57, 113.52, 112.73, 104.40, 60.06, 56.02 ppm; IR (KBr): $\tilde{\nu}$ = 3404, 3273, 3184, 2996, 2935, 2906, 2830, 1683, 1664, 1608, 1584, 1561, 1510, 1487, 1464, 1432, 1411, 1394, 1331, 1305, 1280, 1238, 1210, 1188, 1166, 1128, 1071, 1037, 998, 980, 885, 854, 829, 795, 774, 699, 668, 627, 613, 509 cm^{-1}; UV/Vis: λ (nm) = 269, 230, 209; HRMS (ESI) found: [M+Na$^+$] m/z = 402.1300, calcd. for $C_{22}H_{21}NNaO_5$: 402.1300.

Dibenzyl (4''-carbamoyl-3,4,5-trimethoxy-[1,1':3',1''-terphenyl]-5'-yl) phosphate (**5f**)

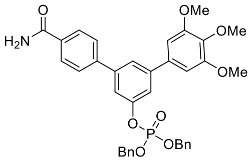

Compound **5f** was synthesized from compound **4f** (90 mg, 236 µmol) according to Method 2. The crude product was purified by flash column chromatography (dichloromethane → dichloromethane/acetone 3:1) and 54 mg (36 %) of a colorless oil were obtained.

R_f = 0.45 (acetone/DCM 1:1); ^{1}H NMR (400 MHz, Methanol-d_4) δ 7.98 – 7.93 (m, 2H), 7.71 – 7.63 (m, 3H), 7.39 – 7.28 (m, 12H), 6.92 – 6.80 (m, 2H), 5.27 – 5.14 (m, 4H), 3.94 – 3.85 (m, 6H), 3.84 – 3.75 (m, 3H) ppm; ^{31}P NMR (162 MHz, CD_3OD) δ -7.20 ppm; IR (Film): $\tilde{\nu}$ = 2938, 2830, 2542, 2434, 2360, 1662, 1607, 1584, 1561, 1510, 1488, 1465, 1431, 1395, 1334, 1305, 1278, 1238, 1187, 1166, 1128, 1071, 1036, 999, 923, 885, 875, 853, 829, 793, 775, 742, 698, 668, 628, 610, 527, 501, 479 cm^{-1}; UV/Vis: λ (nm) = 265, 205 nm; HRMS (ESI) found: [M+Na$^+$] m/z = 662.1926, calcd. for $C_{36}H_{34}NaNO_8P$: 662.1915.

(4''-Carbamoyl-3,4,5-trimethoxy-[1,1':3',1''-terphenyl]-5'-yl) dihydrogen phosphate (**6f**)

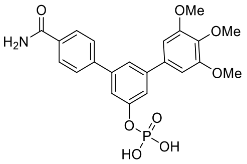

Compound **6f** was synthesized from compound **5f** (27 mg, 42 µmol) according to Method 3. After removing the solvent, the residue was dissolved in methanol and filtered over cotton. Thereby 17 mg (86 %) of a colorless solid were obtained.

R_f (RP) = 0.80 (water/acetonitrile 1:1); Melting point = 207 °C; ^{1}H NMR (400 MHz, Methanol-*d*$_4$) δ 8.00 – 7.91 (m, 2H), 7.87 – 7.78 (m, 2H), 7.67 – 7.57 (m, 2H), 7.42 (dd, *J* = 1.7, 1.7 Hz, 1H), 6.99 (s, 2H), 3.93 (s, 6H), 3.81 (s, 3H) ppm; ^{13}C NMR (101 MHz, CD_3OD) δ 172.23, 157.56 (d, *J* = 6.0 Hz), 154.65, 146.32, 143.81, 142.20, 139.05, 138.67, 133.41, 129.13, 128.26, 119.86 (d, *J* = 4.9 Hz), 119.48, 119.22 (d, *J* = 4.7 Hz), 105.87, 61.20, 56.79 ppm; ^{31}P NMR (162 MHz, CD_3OD) δ 0.54 ppm; IR (KBr): $\tilde{\nu}$ = 3432, 2934, 2839, 2359, 1662, 1582, 1511, 1464, 1430, 1407, 1392, 1361, 1300, 1244, 1183, 1164, 1127, 999, 976, 894, 852, 833, 802, 778, 755, 700, 669, 647, 627, 564, 535, 506 cm^{-1}; UV/Vis: λ (nm) = 270, 210; HRMS (ESI) found: [M-H$^+$] m/z = 458.1000, calcd. for $C_{22}H_{21}NO_8P$: 458.1010.

Ethyl 5'-hydroxy-3'',4'',5''-trimethoxy-[1,1':3',1''-terphenyl]-4-carboxylate (**4g**)

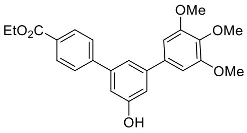

82 mg (216 µmol) of carboxylic acid **4h** were dissolved in 1.35 ml of dry ethanol and 43 mg (70 µmol) of oxone ($KHSO_5$ x 0.5 $KHSO_4$ x 0.5 K_2SO_4) were added. The reaction mixture was stirred at 65 °C for one week. After completion of the reaction the crude product was filtered and purified by flash column chromatography (hexane/ethyl acetate 4:1). After removal of the solvent, 68 mg of compound **4g** (169 µmol, 78 %) were obtained as a pale-yellow oil.

R_f = 0.30 (hexane/ethyl acetate 3:2); ^{1}H NMR (400 MHz, $CDCl_3$) δ 8.16 – 8.09 (m, 2H), 7.70 – 7.63 (m, 2H), 7.33 (dd, *J* = 1.5, 1.5 Hz, 1H), 7.10 – 7.03 (m, 2H), 6.79 (s, 2H), 5.61 (s, 1H), 4.42 (q, *J* = 7.1 Hz, 2H), 3.92 (s, 6H), 3.91 (s, 3H), 1.42 (t, *J* = 7.1 Hz, 3H) ppm; ^{13}C NMR (101 MHz, $CDCl_3$) δ 166.75, 156.64, 153.64, 145.30, 143.94, 142.34, 138.05, 136.81, 130.25, 129.71, 127.28, 118.87, 114.13, 113.40, 104.70, 61.28, 61.15, 56.41, 14.49 ppm; IR (KBr): $\tilde{\nu}$ = 3649, 3431, 2981, 2965, 2931, 2850, 2832, 1705, 1608, 1584, 1512, 1486, 1463, 1438, 1412, 1394, 1369, 1323, 1305, 1285, 1275, 1241, 1184, 1164, 1127, 1070, 1020, 997, 980, 887, 871, 848, 828, 772, 717, 703, 666, 557, 528, 512, 501, 487 cm^{-1}; UV (DCM): λ_{max} = 272; 229 nm; HRMS (ESI) found: [M+H$^+$] m/z = 409.1659, calcd. for $C_{24}H_{24}O_6$: 409.1646.

Ethyl 5'-((bis(benzyloxy)phosphoryl)oxy)-3'',4'',5''-trimethoxy-[1,1':3',1''-terphenyl]-4-carboxylate (**5g**)

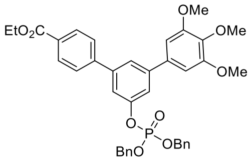

Compound **4g** was synthesized from compound **5g** (97 mg, 237µmol) according to Method 2. The crude product was purified by flash column chromatography (hexane/ethyl acetate 2:1) and 57 mg (36 %) of a colorless oil was obtained.

R_f = 0.30 (hexane/ethyl acetate 3:2); ^{1}H NMR (400 MHz, $CDCl_3$) δ 8.14 – 8.07 (m, 2H), 7.58 (d, *J* = 8.5 Hz, 2H), 7.54 (s, 1H), 7.37 – 7.29 (m, 12H), 6.73 (s, 2H), 5.25 – 5.11 (m, 4H), 4.42 (q, *J* = 7.1 Hz, 2H), 3.90 (s, 9H), 1.43 (t, *J* = 7.1 Hz, 3H) ppm; ^{13}C NMR (101 MHz, $CDCl_3$) δ 166.51, 153.70, 151.41 (d, *J* = 7.0 Hz), 144.38, 143.99, 142.36, 138.38, 135.95, 135.53, 130.26, 130.02, 128.90, 128.79, 128.23, 127.31, 122.98, 118.69 (d, *J* = 5.1 Hz), 117.88 (d, *J* = 4.6 Hz), 104.78, 70.30 (d, *J* = 5.8 Hz), 61.25, 61.14, 56.46, 14.51 ppm; ^{31}P NMR (162 MHz, Chloroform-*d*) δ -6.13 ppm; IR (Film): $\tilde{\nu}$ = 3475, 3064, 3032, 2977, 2959, 2937, 2903, 2840, 1713, 1606, 1580, 1510, 1462, 1456, 1431, 1407, 1390, 1366, 1358, 1275, 1243, 1214, 1180, 1162, 1127, 1105, 1034, 1006, 989, 916, 883, 856, 831, 773, 742, 698, 665, 638, 598, 547, 510, 492, 460, 449, 425, 404 cm^{-1}; UV (DCM): λ_{max} = 234; 273 nm; HRMS (ESI) found: [M+H$^+$] m/z = 669.2233, calcd. for $C_{38}H_{37}O_9P$: 669.2248.

Ethyl 3'',4'',5''-trimethoxy-5'-(phosphonooxy)-[1,1':3',1''-terphenyl]-4-carboxylate (**6g**)

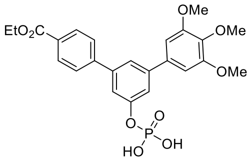

Compound **6g** was synthesized from compound **5g** (58 mg, 87 µmol) according to Method 3. Thereby 28 mg (66 %) of a colorless solid were obtained.

R_f (RP) = 0.60 (water/acetonitrile 1:1); Melting point = 150 °C; ^{1}H NMR (400 MHz, CD_3OD) δ 8.09 (d, *J* = 7.8 Hz, 2H), 7.82 (d, *J* = 7.8 Hz, 2H), 7.58 (s, 1H), 7.53 (s, 2H), 6.96 (s, 2H), 4.39 (q, *J* = 7.1 Hz, 2H), 3.92 (s, 6H), 3.81 (s, 3H), 1.41 (t, *J* = 7.1 Hz, 3H) ppm; ^{13}C NMR (101 MHz, CD_3OD) δ 167.97, 154.81, 146.66, 144.49, 142.17, 139.03, 138.15, 131.03, 130.15, 128.32, 121.72, 120.07, 119.28, 105.83, 62.17, 61.19, 56.80, 14.62 ppm; ^{31}P NMR (162 MHz, Methanol-*d*$_4$) δ -4.87 ppm; IR (KBr): $\tilde{\nu}$ = 3442, 2926, 2851, 1713, 1637, 1607, 1583, 1511, 1464, 1433, 1408, 1391, 1366, 1295, 1278, 1242, 1182, 1163, 1126, 1106, 1073, 938, 904, 853, 833, 774, 706, 666, 557, 531, 512, 504, 492 cm^{-1}; UV (DCM): λ_{max}= 272, 211 nm; HRMS (ESI) found: [M-H$^+$] m/z = 487.1147, calcd. for $C_{24}H_{24}O_9P$: 487.1163.

5'-Hydroxy-3'',4'',5''-trimethoxy-[1,1':3',1''-terphenyl]-4-carboxylic acid (**4h**)

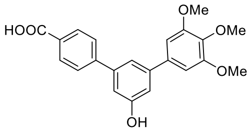

Compound **4h** was synthesized from compound **3** according to Method 1. The crude product was purified by flash column chromatography (hexane/ethyl acetate 3:2 and 0.5 % HOAc) and 74 mg (82 %) of a colorless solid were obtained.

R_f = 0.20 (hexane/ethyl acetate 1:1); Melting point = 234 °C; ^{1}H NMR (400 MHz, Methanol-d_4) δ 8.13 – 8.07 (m, 2H), 7.78 – 7.73 (m, 2H), 7.33 (dd, *J* = 1.6, 1.6 Hz, 1H), 7.08 – 7.02 (m, 2H), 6.90 (s, 2H), 3.92 (s, 6H), 3.81 (s, 3H) ppm; ^{13}C NMR (101 MHz, CD_3OD) δ 169.76, 159.44, 154.78, 147.01, 144.76, 143.19, 138.97, 138.64, 131.29, 130.86, 128.14, 118.36, 114.86, 114.04, 105.72, 61.20, 56.75 ppm; IR (KBr): $\tilde{\nu}$ = 3417, 3001, 2939, 2834, 2671, 2536, 1691, 1607, 1584, 1512, 1486, 1464, 1434, 1412, 1392, 1368, 1303, 1240, 1183, 1166, 1126, 998, 923, 884, 850, 830, 798, 777, 747, 707, 666, 521, 474, 457 cm^{-1}; UV/Vis: λ (nm) = 273, 229, 211; HRMS (ESI) found: [M+H$^+$] m/z = 381.1319, calcd. for $C_{22}H_{21}O_6$: 381.1333.

Benzyl 5'-hydroxy-3'',4'',5''-trimethoxy-[1,1':3',1''-terphenyl]-4-carboxylate (**4h'**)

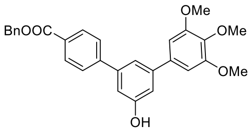

48 mg (126 µmol) of acid **4h** were dissolved in 0.5 ml of dry DMF and 15 mg (151 µmol, 1.2 eq.) of $KHCO_3$ and 36 µl (332 µmol, 2.6 eq.) of benzyl bromide were added. After stirring for 48 hours, the reaction was quenched by adding 5 ml of water. The mixture was extracted three times with ethyl acetate and washed with a 5 % $NaHCO_3$ solution. Purification of the crude product **4h'** was done by flash column chromatography (hexane/ethyl acetate 9:1 → 3:2) and yielded 45 mg (76 %) of a colorless oil.

R_f = 0.50 (hexane/ethyl acetate 1:1); Melting point = 42 °C; ^{1}H NMR (400 MHz, Chloroform-*d*) δ 8.16 (d, *J* = 8.3 Hz, 2H), 7.67 (d, *J* = 8.3 Hz, 2H), 7.52 – 7.31 (m, 6H), 7.08 – 7.03 (m, 2H), 2H), 6.79 (s, 2H), 5.40 (s, 2H), 5.26 (s, 1H), 3.92 (s, 6H), 3.91 (s, 3H) ppm; ^{13}C NMR (101 MHz, $CDCl_3$) δ 166.47, 156.52, 153.65, 145.48, 143.98, 142.31, 138.09, 136.72, 136.15, 130.43, 129.38, 128.79, 128.46, 128.35, 127.34, 118.97, 114.14, 113.37, 104.69, 66.98, 61.15, 56.41 ppm; IR (Film): $\tilde{\nu}$ = 3036, 3002, 2935, 2836, 2251, 1956, 1715, 1606, 1583, 1513, 1487, 1455, 1434, 1412, 1392, 1374, 1304, 1274, 1244, 1183, 1166, 1122, 1102, 1016, 999, 981, 910, 882, 849, 831, 773, 733, 700, 667, 648, 639, 602, 587, 528 cm^{-1}; UV/Vis: λ (nm) = 274, 228, 220, 214; HRMS (ESI) found: [M+H$^+$] m/z = 471.1799, calcd. for $C_{29}H_{27}O_6$: 471.1802.

Benzyl 5'-((bis(benzyloxy)phosphoryl)oxy)-3'',4'',5''-trimethoxy-[1,1':3',1''-terphenyl]-4-carboxylate (**5h**)

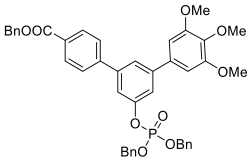

Compound **5h** was synthesized from compound **4h'** (40 mg, 85 µmol) according to Method 2. The crude product was purified by flash column chromatography (hexane/ethyl acetate 9:1 → 2:1) yielding 46 mg (74 %) of the product as a colorless oil.

R_f = 0.55 (hexane/ethyl acetate 1:1); ^{1}H NMR (400 MHz, Chloroform-*d*) δ 8.22 – 8.01 (m, 2H), 7.61 – 7.28 (m, 20H), 6.72 (s, 2H), 5.41 (s, 2H), 5.25 – 5.07 (m, 4H), 3.90 (s, 9H) ppm; ^{13}C NMR (101 MHz, $CDCl_3$) δ 166.30, 153.71, 151.42 (d, *J* = 7.1 Hz), 144.63, 144.00, 142.28, 138.40, 136.17, 135.92, 135.49 (d, *J* = 6.9 Hz), 130.43, 129.64, 128.90, 128.79, 128.46, 128.34, 128.23, 127.37, 122.97, 118.72 (d, *J* = 4.9 Hz), 117.88 (d, *J* = 4.9 Hz), 104.79, 70.30 (d, *J* = 5.8 Hz), 66.97, 61.14, 56.46 ppm; ^{31}P NMR (162 MHz, $CDCl_3$) δ -6.17 ppm; IR (Film): $\tilde{\nu}$ = 3067, 3014, 2961, 2916, 2898, 2848, 1716, 1607, 1581, 1541, 1509, 1498, 1464, 1457, 1432, 1409, 1391, 1359, 1273, 1238, 1216, 1181, 1162, 1128, 1104, 1037, 1006, 989, 966, 918, 885, 854, 832, 754, 697, 667, 619, 601, 509, 494, 457, 418, 413 cm^{-1}; UV/Vis: λ (nm) = 273, 232; HRMS (ESI) found: [M+Na^+] m/z = 753.2230, calcd. for $C_{43}H_{39}NaO_6P$: 753.2224.

3'',4'',5''-Trimethoxy-5'-(phosphonoxy)-[1,1':3',1''-terphenyl]-4-carboxylic acid (**6h**)

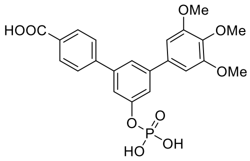

Compound **6h** was synthesized from compound **5h** (45 mg, 59 µmol) according to Method 3. Thereby 19 mg (70 %) of a colorless solid were obtained.

R_f = 0.40 (water/acetonitrile 2:1); Melting point = 152 °C; ^{1}H NMR (400 MHz, Methanol-d_4) δ 8.11 (d, *J* = 7.9 Hz, 2H), 7.82 (d, *J* = 7.9 Hz, 2H), 7.63 (s, 1H), 7.52 (s, 2H), 6.96 (s, 2H), 3.93 (s, 6H), 3.81 (s, 3H) ppm; ^{13}C NMR (101 MHz, CD_3OD) δ 169.65, 154.84, 146.32, 144.63, 143.03, 139.10, 137.93, 131.35, 131.00, 128.25, 122.24, 119.90, 119.17, 105.79, 61.19, 56.78 ppm; ^{31}P NMR (162 MHz, CD_3OD) δ -5.20 ppm; IR (KBr): $\tilde{\nu}$ = 3447, 3005, 2937, 2837, 1700, 1606, 1583, 1512, 1465, 1433, 1409, 1391, 1358, 1297, 1241, 1180, 1163, 1126, 1072, 1037, 1006, 991, 947, 903, 883, 855, 831, 777, 706, 666, 624, 554, 528, 505, 499 cm^{-1}; UV/Vis: λ (nm) = 261, 228, 215, 207; HRMS (ESI) found: [M-H^+] m/z = 459.0853, calcd. for $C_{22}H_{20}O_9P$: 459.0850.

4''-Isopropyl-3,4,5-trimethoxy-[1,1':3',1''-terphenyl]-5'-ol (**4i**)

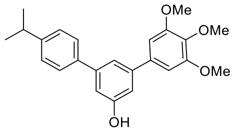

Compound **4i** was synthesized from compound **3** according to Method 1. The crude product was purified by flash column chromatography (hexane/ethyl acetate 9:1 → 2:1) and 85 mg (95 %) of a colorless solid were obtained.

R_f (RP) = 0.40 (hexane/ethyl acetate 3:2); Melting point >260 °C; ^{1}H NMR (400 MHz, Chloroform-*d*) δ 7.59 – 7.50 (m, 2H), 7.37 – 7.29 (m, 3H), 7.05 – 6.93 (m, 2H), 6.80 (s, 2H), 4.99 (s, 1H), 3.92 (s, 6H), 3.90 (s, 3H), 2.97 (hept, *J* = 6.9 Hz, 1H), 1.30 (d, *J* = 6.9 Hz, 6H) ppm; ^{13}C NMR (101 MHz, $CDCl_3$) δ 156.26, 153.59, 148.69, 143.62, 143.59, 138.40, 137.95, 137.02, 127.30, 127.07, 118.92, 113.12, 112.94, 104.63, 61.13, 56.38, 33.99, 24.15 ppm; IR (KBr): $\tilde{\nu}$ = 3742, 3444, 2958, 2928, 2360, 2329, 1644, 1583, 1514, 1460, 1392, 1303, 1240, 1165, 1126, 1002, 829, 752, 698, 420 cm^{-1}; UV/Vis: λ (nm) = 261, 229, 214; HRMS (ESI) found: [M+Na^{+}] m/z = 401.1730, calcd. for $C_{24}H_{26}NaO_4$: 401.1723.

Dibenzyl (4''-isopropyl-3,4,5-trimethoxy-[1,1':3',1''-terphenyl]-5'-yl) phosphate (**5i**)

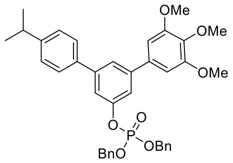

Compound **5i** was synthesized from compound **4i** (78 mg, 206 µmol) according to Method 2. The crude product was purified by flash column chromatography (hexane/ethyl acetate 6:1 → 2:1) and 112 mg (85 %) of a colorless oil were obtained.

R_f = 0.50 (hexane/ethyl acetate 3:2); ^{1}H NMR (400 MHz, Chloroform-*d*) δ 7.53 (s, 1H), 7.50 – 7.44 (m, 2H), 7.37 – 7.28 (m, 14H), 6.73 (s, 2H), 5.21 – 5.11 (m, 4H), 3.90 (s, 3H), 3.89 (s, 6H), 2.97 (hept, *J* = 7.0 Hz, 1H), 1.31 (d, *J* = 7.0 Hz, 6H) ppm; ^{13}C NMR (101 MHz, $CDCl_3$) δ 153.63, 151.31 (d, *J* = 7.1 Hz), 148.94, 143.62, 143.55, 137.71, 136.29, 135.61, 135.54, 128.82, 128.75, 128.20, 127.35, 127.09, 122.79, 117.50 (d, *J* = 4.0 Hz), 117.38 (d, *J* = 5.2 Hz), 104.70, 70.20 (d, *J* = 5.7 Hz), 61.13, 56.41, 34.00, 24.14 ppm; ^{31}P NMR (162 MHz, $CDCl_3$) δ -6.13 ppm; IR (Film): $\tilde{\nu}$ = 2829, 1605, 1580, 1509, 1464, 1457, 1432, 1411, 1393, 1360, 1296, 1241, 1216, 1180, 1162, 1129, 1036, 1008, 990, 919, 831, 755, 696, 666, 602, 491, 413 cm^{-1}; UV/Vis: λ (nm) = 263, 229, 215; HRMS (ESI) found: [M+H^{+}] m/z = 639.2484, calcd. for $C_{38}H_{40}O_7P$: 639.2506.

(4''-Isopropyl-3,4,5-trimethoxy-[1,1':3',1''-terphenyl]-5'-yl) dihydrogen phosphate (**6i**)

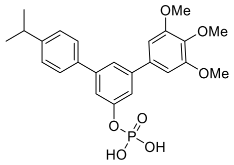

Compound **6i** was synthesized from compound **5i** (80 mg, 125 µmol) according to Method 3. Thereby 39 mg (68 %) of a colorless solid were obtained.

R_f (RP) = 0.25 (water/acetonitrile 2:1); Melting point = 182 °C; ^{1}H NMR (400 MHz, Methanol-*d*$_4$) δ 7.64 – 7.54 (m, 3H), 7.46 – 7.39 (m, 2H), 7.32 (d, *J* = 7.6 Hz, 2H), 6.92 (s, 2H), 3.91 (s, 6H), 3.80 (s, 3H), 2.94 (hept, *J* = 7.0 Hz, 1H), 1.28 (d, *J* = 7.0 Hz, 6H) ppm; ^{13}C NMR (101 MHz, CD_3OD) δ 154.82, 153,87, 149.84, 144.50, 144.46, 139.14, 139.08, 138.01, 128.12, 127.94, 122.44, 118.69, 105.75, 61.19, 56.76, 35.08, 24.40 ppm; ^{31}P NMR (162 MHz, CD_3OD) δ -5.45 ppm; IR (KBr): $\tilde{\nu}$ = 3676, 3669, 3656, 3648, 3628, 3566, 3444, 2959, 2367, 1654, 1647, 1582, 1513, 1464, 1459, 1434, 1411, 1394, 1361, 1297, 1242, 1161, 1127, 1036, 1007, 991, 955, 904, 830, 694, 413 cm^{-1}; UV/Vis: λ (nm) = 263, 229, 217, 204; HRMS (ESI) found: [M-H$^+$] m/z = 457.1423, calcd. for $C_{24}H_{26}O_7P$: 457.1421.

4''-(*tert*-Butyl)-3,4,5-trimethoxy-[1,1':3',1''-terphenyl]-5'-ol (**4j**)

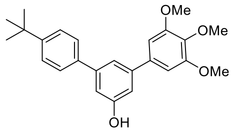

Compound **4j** was synthesized from compound **3** according to Method 1. The crude product was purified by flash column chromatography (hexane/ethyl acetate 9:1 → 3:1) and 93 mg (98 %) of a colorless solid were obtained.

R_f = 0.45 (hexane/ethyl acetate 3:2); Melting point = 167 °C; ^{1}H NMR (400 MHz, Chloroform-*d*) δ 7.56 (d, *J* = 8.4 Hz, 2H), 7.48 (d, *J* = 8.4 Hz, 2H), 7.33 (dd, *J* = 1.5, 1.5 Hz, 1H), 7.03 (dd, *J* = 2.5, 1.5 Hz, 1H), 7.00 (dd, *J* = 2.5, 1.5 Hz, 1H), 6.80 (s, 2H), 5.18 (s, 1H), 3.92 (s, 6H), 3.90 (s, 3H), 1.37 (s, 9H) ppm; ^{13}C NMR (101 MHz, $CDCl_3$) δ 156.26, 153.60, 150.96, 143.61, 143.48, 137.98, 137.94, 137.02, 127.02, 125.93, 118.92, 113.11, 112.96, 104.61, 61.13, 56.37, 34.74, 31.51 ppm; IR (KBr): $\tilde{\nu}$ = 3688, 3675, 3668, 3648, 3433, 2961, 2904, 2866, 2835, 1733, 1716, 1700, 1684, 1635, 1584, 1514, 1483, 1462, 1433, 1410, 1392, 1364, 1303, 1271, 1239, 1185, 1165, 1127, 998, 980, 887, 828, 726, 684, 568, 558, 526 cm^{-1}; UV/Vis: λ (nm) = 261, 233, 210; HRMS (ESI) found: [M+H$^+$] m/z = 393.2057, calcd. for $C_{25}H_{29}O_4$: 393.2061.

Dibenzyl (4''-(*tert*-butyl)-3,4,5-trimethoxy-[1,1':3',1''-terphenyl]-5'-yl) phosphate (**5j**)

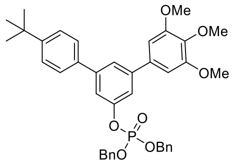

Compound **5j** was synthesized from compound **4j** (79 mg, 201 µmol) according to Method 2. The crude product was purified by flash column chromatography (hexane/ethyl acetate 9:1 → 3:1) yielding 94 mg (72 %) of a colorless oil.

R_f = 0.50 (hexane/ethyl acetate 3:2); ^{1}H NMR (400 MHz, Chloroform-*d*) δ 7.54 (s, 1H), 7.48 (s, 3H), 7.37 – 7.29 (m, 11H), 7.27 (s, 2H), 6.73 (s, 2H), 5.28 – 5.07 (m, 4H), 3.90 (s, 3H), 3.89 (s, 6H), 1.38 (s, 9H) ppm; ^{13}C NMR (101 MHz, $CDCl_3$) δ 153.64, 151.33 (d, *J* = 7.1 Hz), 151.21, 143.62, 143.45, 137.31, 136.29, 135.62, 135.55, 128.81, 128.76, 128.20, 127.08, 125.95, 122.78, 117.64 (d, J = 4.5 Hz), 117.53 (d, *J* = 5.3 Hz), 104.71, 70.20 (d, *J* = 5.6 Hz), 61.13, 56.41, 34.76, 31.50 ppm; ^{31}P NMR (162 MHz, $CDCl_3$) δ -6.13 ppm; IR (Film): $\tilde{\nu}$ = 3445, 3033, 3006, 2961, 2901, 2866, 2829, 1604, 1579, 1511, 1463, 1430, 1407, 1390, 1360, 1296, 1240, 1214, 1180, 1161, 1128, 1036, 1007, 989, 918, 880, 854, 831, 747, 697, 683, 666, 600, 491, 418, 412 cm^{-1}; UV/Vis: λ (nm) = 263, 230, 213; HRMS (ESI) found: [M+Na^{+}] m/z = 675.2500, calcd. for $C_{39}H_{41}NaO_7P$: 675.2482.

(4''-(*tert*-Butyl)-3,4,5-trimethoxy-[1,1':3',1''-terphenyl]-5'-yl) dihydrogen phosphate (**6j**)

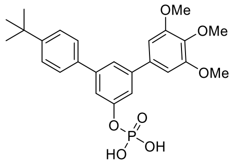

Compound **6j** was synthesized from compound **5j** (35 mg, 54 µmol) according to Method 3. The crude product was purified by reversed-phase column chromatography (water: acetonitrile 2:1) and 15 mg (90 %) of a colorless solid were obtained.

R_f (RP) = 0.35 (water/acetonitrile 4:3); Melting point = 140 °C; ^{1}H NMR (400 MHz, Methanol-*d*$_4$) δ 7.70 – 7.59 (m, 2H), 7.51 – 7.45 (m, 5H), 6.96 (s, 2H), 3.92 (s, 6H), 3.81 (s, 3H), 1.36 (s, 9H) ppm; ^{13}C NMR (101 MHz, CD_3OD) δ 155.61 (d, J = 6.6 Hz), 154.73, 151.61, 143.98, 143.83, 139.29, 138.82, 138.71, 127.83, 126.68, 120.99, 119.02 (d, J = 4.8 Hz), 118.91 (d, J = 4.2 Hz), 105.79, 61.20, 56.77, 35.36, 31.79 ppm; ^{31}P NMR (162 MHz, CD_3OD) δ -4.37 ppm; IR (KBr): $\tilde{\nu}$ = 3434, 2960, 2359, 2340, 1651, 1645, 1583, 1514, 1463, 1434, 1410, 1391, 1362, 1302, 1240, 1186, 1163, 1126, 1072, 1001, 906, 831, 701, 586, 567, 505 cm^{-1}; UV/Vis: λ (nm) = 263, 232, 219; HRMS (ESI) found: [M-H^{+}] m/z = 471.1578, calcd. for $C_{25}H_{28}O_7P$: 471.1578.

Diethyl ((3-bromo-5-iodophenyl)(hydroxy)methyl) phosphonate (**8**)

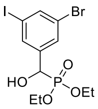

0.90 g (2.90 mmol) of 3-bromo-5-iodobenzaldehyde (**7**) were dissolved in 5 ml of MeCN and then 0.37 ml (3.18 mmol; 1.1 eq.) of diethyl phosphite and 0.88 ml (6.37 mmol; 2.2 eq.) of triethylamine were added dropwise. After 14 h of stirring, the solvent was removed and the residue was purified by flash column chromatography (hexane/ethyl acetate 19:1 → 5:1). Thereby 1.10 g (81 %) of a pale-yellow solid were obtained.

R_f = 0.20 (hexane/ethyl acetate 1:1); Melting point = 84 °C; ^{1}H NMR (400 MHz, Chloroform-*d*) δ 7.79 (s, 1H), 7.76 (s, 1H), 7.61 (s, 1H), 4.94 (d, *J* = 11.5 Hz, 1H), 4.20 – 3.99 (m, 4H), 1.34 – 1.24 (m, 6H) ppm; ^{13}C NMR (76 MHz, Chloroform-*d*) δ 141.01 (d, *J* = 1.8 Hz), 139.10 (d, *J* = 3.3 Hz), 134.74 (d, *J* = 5.4 Hz), 129.57 (d, *J* = 5.4 Hz), 122.83 (d, *J* = 3.1 Hz), 94.10 (d, *J* = 3.3 Hz), 69.44 (d, *J* = 159.5 Hz), 64.15 (d, *J* = 7.1 Hz), 63.51 (d, *J* = 7.5 Hz), 16.53 (d, *J* = 5.5 Hz), 16.51 (d, *J* = 6.0 Hz) ppm; ^{31}P NMR (162 MHz, $CDCl_3$) δ 19.83 ppm; IR (KBr): $\tilde{\nu}$ = 3068, 2982, 2928, 2906, 2865, 2828, 2648, 2359, 1635, 1579, 1550, 1475, 1441, 1415, 1392, 1368, 1289, 1276, 1246, 1229, 1199, 1162, 1111, 1100, 1043, 1022, 976, 893, 861, 821, 784, 724, 679, 640, 576, 538, 477, 424 cm^{-1}; UV/Vis: λ (nm) = 258, 231, 215, 203 nm; HRMS (ESI) found: [M+Na$^+$] m/z = 470.8841, calcd. for $C_{11}H_{15}BrNaO_4P$: 470.8828.

Diethyl ((3-bromo-5-iodophenyl)fluoromethyl) phosphonate (**9**)

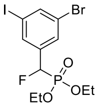

1.6 ml (12.4 mmol; 1.2 eq.) of DAST were dissolved in 22 ml of dry DCM and cooled down to -78°C. 4.6 g (10.3 mmol; 1 eq.) of α-hydroxy phosphonate **8** in 44 ml of dry DCM were added dropwise and the mixture was stirred for 2 h at the same temperature. After slowly warming up to rt, the mixture was stirred for another 15 h. The reaction was quenched by adding ca. 200 ml of a saturated $NaHCO_3$ solution, extracted three times with DCM and dried over $MgSO_4$. The crude product was purified by flash column chromatography (hexane/ethyl acetate 19:1 → 3:1) yielding 2.4 g (51 %) of a colorless solid.

R_f = 0.35 (hexane/ethyl acetate 3:2); Melting point = 45° C; ^{1}H NMR (400 MHz, Chloroform-*d*) δ 7.85 (s, 1H), 7.72 (s, 1H), 7.57 (s, 1H), 5.58 (dd, *J* = 44.7, 8.5 Hz, 1H), 4.32 – 3.89 (m, 4H), 1.37 – 1.19 (m, 6H) ppm; ^{13}C NMR (101 MHz, Chloroform-*d*) δ 140.36 (dd, *J* = 2.7, 1.7 Hz), 137.08 (dd, *J* = 19.1, 1.3 Hz), 134.13 (dd, *J* = 7.3, 5.4 Hz), 129.03 (dd, *J* = 7.4, 5.2 Hz), 123.16 (d, *J* = 2.8 Hz), 94.30 (d, *J* = 2.7 Hz), 87.82 (dd, *J* = 187.4, 169.2 Hz), 64.26 (d, *J* = 7.0 Hz), 63.85 (d, *J* = 6.9 Hz), 16.53 (d, *J* = 5.6 Hz) ppm; ^{19}F NMR (377 MHz, Chloroform-*d*) δ -203.32

(dd, J = 81.2, 44.7 Hz) ppm; ^{31}P NMR (162 MHz, Chloroform-*d*) δ 13.46 (d, J = 81.2 Hz) ppm; IR (KBr): $\tilde{\nu}$ = 3627, 3444, 3065, 2983, 2932, 2908, 2867, 1636, 1630, 1582, 1552, 1477, 1442, 1418, 1392, 1368, 1290, 1265, 1251, 1227, 1192, 1163, 1111, 1098, 1056, 1029, 976, 905, 862, 817, 805, 789, 753, 726, 680, 648, 576 cm^{-1}; UV/Vis: λ (nm) = 234, 220; HRMS (ESI) found: [M+Na$^+$] m/z = 472,8775, calcd. for $C_{11}H_{14}BrFINaO_3P$: 472,8785.

Diethyl (fluoro-(5-bromo-3',4',5'-trifluoro-[1,1'-biphenyl]-3-yl)methyl) phosphonate (**10**)

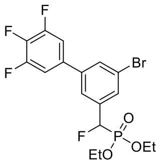

To a degassed solution of 175 mg (388 µmol; 1 eq.) of α-fluorophosphonate **9**, 82 mg (466 µmol; 1.2 eq.) of trifluorophenylboronic acid, 98 mg (1.15 mmol; 3.0 eq.) of $NaHCO_3$ in 2 ml of ethanol and 2 ml of water 18 mg (16 µmol; 0.05 eq.) $Pd(PPh_3)_4$ were added and the mixture was refluxed for 36 h. After extracting three times with ethyl acetate and drying over $MgSO_4$, the crude product was purified by flash column chromatography (hexane/ethyl acetate 5:1 → 2:1) yielding 88 mg (50 %) of a colorless oil.

R_f = 0.20 (hexane/ethyl acetate 3:1); ^{1}H NMR (400 MHz, Chloroform-*d*) δ 7.69 – 7.57 (m, 2H), 7.54 (s, 1H), 7.28 – 7.13 (m, 2H), 5.70 (dd, J = 44.7, 8.4 Hz, 1H), 4.30 – 3.98 (m, 4H), 1.37 – 1.26 (m, 2H) ppm; ^{13}C NMR (101 MHz, $CDCl_3$) δ 151.68 (ddd, J = 251.5, 11.1, 4.0 Hz), 140.48, 139.99 (dt, J = 253.5, 15.1 Hz), 136.40, 136.22, 135.27 (d, J = 5.0 Hz), 130.60, 129.35 (dd, J = 7.6, 5.5 Hz), 123.94 – 123.75 (m), 123.34 (d, J = 2.0 Hz), 111.74 – 111.23 (m), 88.51 (dd, J = 186.7, 169.1 Hz), 64.25 (d, J = 6.9 Hz), 63.77 (d, J = 7.3 Hz), 16.56 (d, J = 5.5 Hz) ppm; ^{19}F NMR (377 MHz, Chloroform-*d*) δ -132.12 – -133.56 (m), -160.95 (tt, J = 20.5, 6.3 Hz), -202.67 (dd, J = 81.4, 44.7 Hz) ppm; ^{31}P NMR (162 MHz, Chloroform-*d*) δ 13.85 (d, J = 81.4 Hz) ppm; IR (KBr): $\tilde{\nu}$ = 3464, 3074, 2986, 2930, 2912, 2871, 2244, 1713, 1618, 1604, 1573, 1532, 1477, 1450, 1429, 1402, 1362, 1255, 1213, 1165, 1115, 1098, 1045, 1031, 976, 910, 876, 854, 819, 788, 734, 706, 689, 663, 580, 545, 490, 448 cm^{-1}; UV/Vis: λ (nm) = 252, 234; HRMS (ESI) found: [M+Na$^+$] m/z = 476.9540, calcd. for $C_{17}H_{16}BrF_4NaO_3P$: 476.9549.

Diethyl (fluoro-(3,4,5-trifluoro-3'',4'',5''-trimethoxy-[1,1':3',1''-terphenyl]-5'-yl)methyl) phosphonate (**11**)

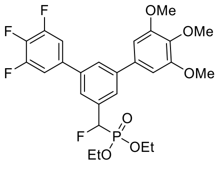

88 mg (193 µmol; 1 eq.) of compound **10**, 82 mg (387 µmol; 2 eq.) of trimethoxyboronic acid and 67 mg (483 µmol; 2.5 mmol) of K_2CO_3 were dissolved in 2 ml of water and 3 ml of ethanol and the solution was degassed. After adding 9 mg (8 µmol; 0.04 eq.) of $Pd(PPh_3)_4$ the mixture was refluxed overnight, then extracted three times with ethyl acetate, washed with brine and dried over Na_2SO_4. The crude product was purified by flash column chromatography (hexane/ethyl acetate 4:1 → 3:2) yielding 54 mg (59 %) of a colorless oil.

R_f = 0.40 (hexane/ethyl acetate 3:2); ^{1}H NMR (400 MHz, Chloroform-*d*) δ 7.65 (s, 1H), 7.63 (s, 1H), 7.58 (s, 1H), 7.31 – 7.19 (m, 2H), 6.78 (s, 2H), 5.81 (dd, *J* = 44.8, 8.1 Hz, 1H), 4.30 – 4.02 (m, 4H), 3.94 (s, 6H), 3.90 (s, 3H), 1.39 – 1.23 (m, 6H) ppm; ^{13}C NMR (101 MHz, $CDCl_3$) δ 153.79, 151.65 (ddd, *J* = 250.2, 10.0, 4.3 Hz), 142.86 (d, *J* = 2.1 Hz), 139.23, 138.46, 136.80 – 136.58 (m), 136.03, 134.80 (dd, *J* = 18.5, 1.4 Hz), 126.49, 125.45 (dd, *J* = 6.2, 6.2 Hz), 124.09 (dd, *J* = 6.1, 6.1 Hz), 111.73 – 111.17 (m), 104.82, 89.29 (dd, *J* = 185.3, 169.3 Hz), 64.09 (d, *J* = 7.0 Hz), 63.59 (d, *J* = 6.9 Hz), 61.14, 56.49, 16.62 (d, *J* = 2.6 Hz), 16.56 (d, *J* = 2.7 Hz) ppm; ^{19}F NMR (377 MHz, Chloroform-*d*) δ -136.85 (ddd, *J* = 20.8, 6.7, 6.7 Hz), -165.81 (tt, *J* = 20.8, 6.4 Hz), -200.94 (dd, *J* = 82.8, 44.8 Hz) ppm; ^{31}P NMR (162 MHz, Chloroform-*d*) δ 14.49 (d, *J* = 82.8 Hz) ppm; IR (Film): $\tilde{\nu}$ = 3444, 3006, 2938, 2870, 2848, 2359, 2341, 1731, 1618, 1584, 1531, 1508, 1465, 1443, 1416, 1396, 1339, 1244, 1217, 1186, 1167, 1130, 1100, 1046, 1032, 976, 892, 857, 834, 757, 708, 667, 646, 584, 542, 491, 445 cm^{-1}; UV/Vis: λ (nm) = 258, 228, 222, 220, 204 nm; HRMS (ESI) found: $[M+Na^+]$ m/z = 565.1385, calcd. for $C_{26}H_{27}F_4NaO_6P$: 565.1374.

(Fluoro(3,4,5-trifluoro-3'',4'',5''-trimethoxy-[1,1':3',1''-terphenyl]-5'-yl)methyl) phosphate (**12**)

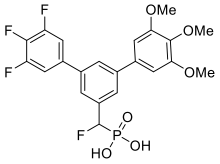

39 mg (72 µmol; 1 eq.) of diethyl phosphonate **11** were dissolved in 1.3 ml of dry DCM and cooled to 0 °C. 0.1 ml (647 µmol; 9 eq.) of TMS-Br were added dropwise and the mixture was stirred for 2 h at the same temperature. After warming up to room temperature, the mixture was stirred overnight. The solvent was removed and the residue was coevaporated three times with DCM and methanol respectively. Purification of compound **12** was done by reversed-phase column chromatography (acetonitrile: water 2:3). Thereby 22 mg (63 %) of a colorless solid were obtained.

R_f (RP) = 0.20 (water/acetonitrile 3:2); Melting point = 124 °C; ^{1}H NMR (400 MHz, Methanol-*d*$_4$) δ 7.79 (s, 1H), 7.75 (s, 1H), 7.67 (s, 1H), 7.57 – 7.50 (m, 2H), 6.98 – 6.96 (m, 2H), 5.83 (d, *J* = 44.2 Hz, 1H), 3.92 (s, 6H), 3.81 (s, 3H) ppm; ^{13}C NMR (101 MHz, CD_3OD) δ 154.90, 152.66 (ddd, *J* = 247.7, 5.7, 4.1 Hz), 151.40, 143.52, 140.32 (dt, *J* = 267.9, 15.2 Hz), 139.90, 139.43,

138.77, 137.93, 137.78, 126.78, 125.51 – 125.34 (m), 112.78 – 112.37 (m), 105.89, 61.19, 56.80 ppm; ^{19}F NMR (377 MHz, Methanol-d_4) δ -137.44 – -137.90 (m), -166.40 – -166.72 (m), -201.66 (dd, J = 81.0, 44.2 Hz) ppm; ^{31}P NMR (162 MHz, Methanol-d_4) δ 11.78 (br) ppm; IR (KBr): $\tilde{\nu}$ = 3446, 3075, 3017, 2971, 2941, 2907, 2835, 2689, 2596, 2400, 2359, 2330, 2159, 1798, 1716, 1618, 1585, 1530, 1509, 1465, 1444, 1417, 1397, 1339, 1270, 1242, 1217, 1188, 1172, 1128, 1044, 1005, 966, 936, 892, 856, 834, 755, 708, 667, 647, 582, 531, 471, 459, 446, 416 cm^{-1}; UV/Vis: λ (nm) = 259, 208 nm; HRMS (ESI) found: $[M-H^+]$ m/z = 485.0777, calcd. for $C_{22}H_{18}F_4O_6P$: 485.0783.

NMR spectra

^{1}H NMR of compound **6a**

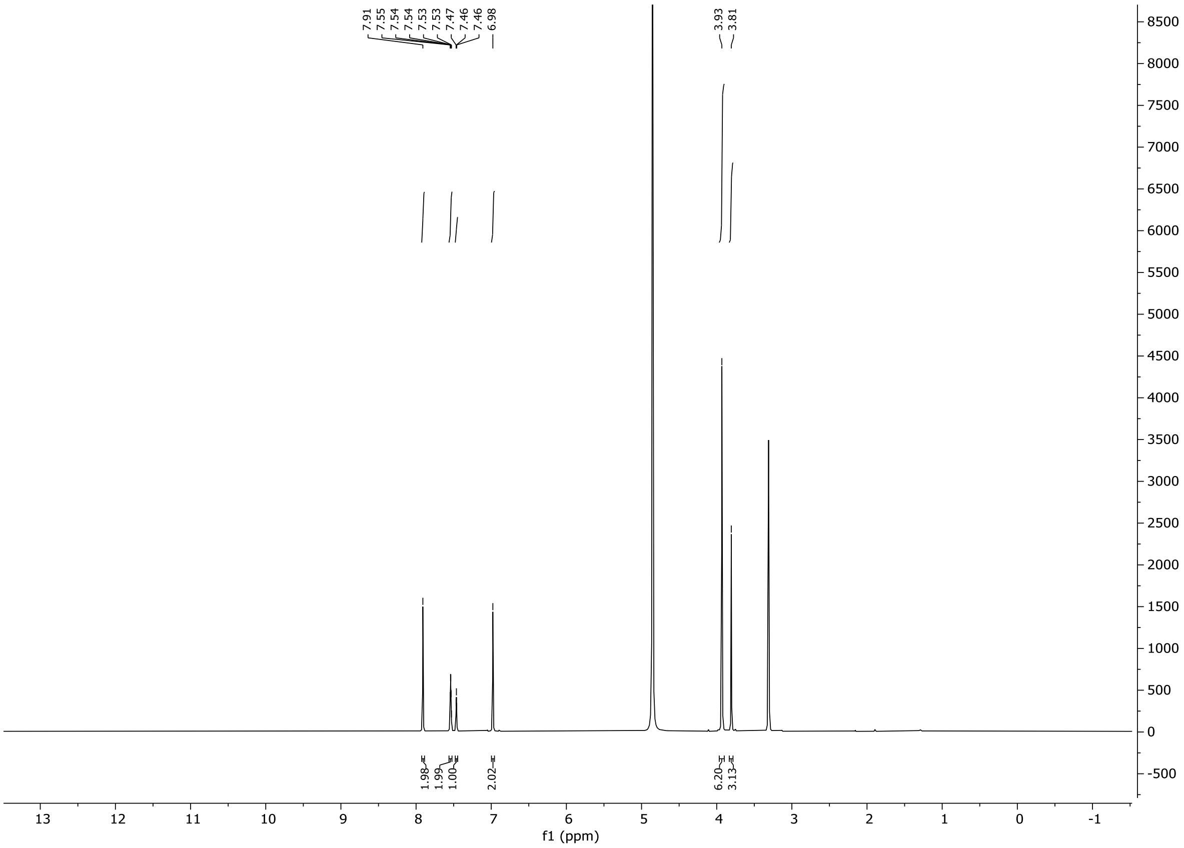

^{13}C NMR of compound **6a**

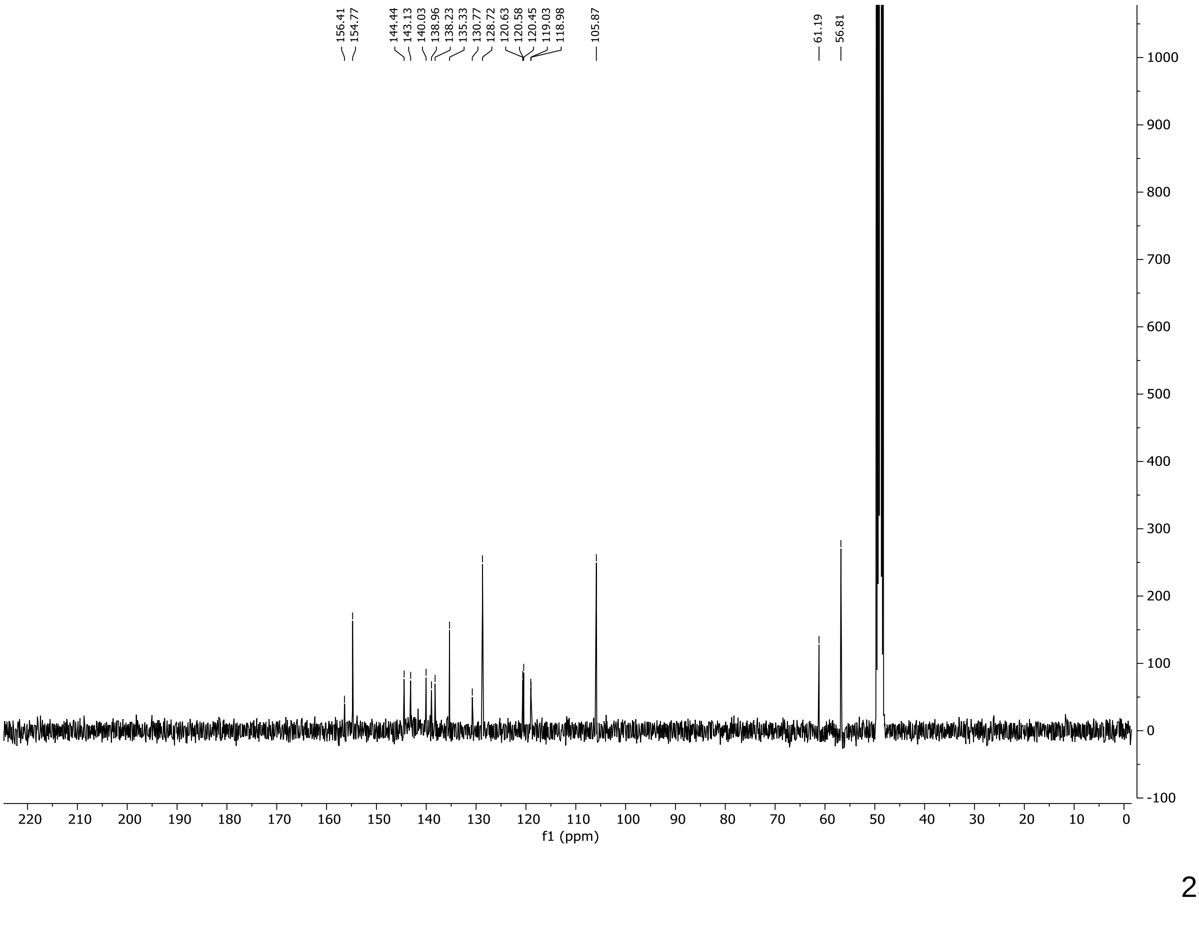

^{31}P NMR of compound **6a**

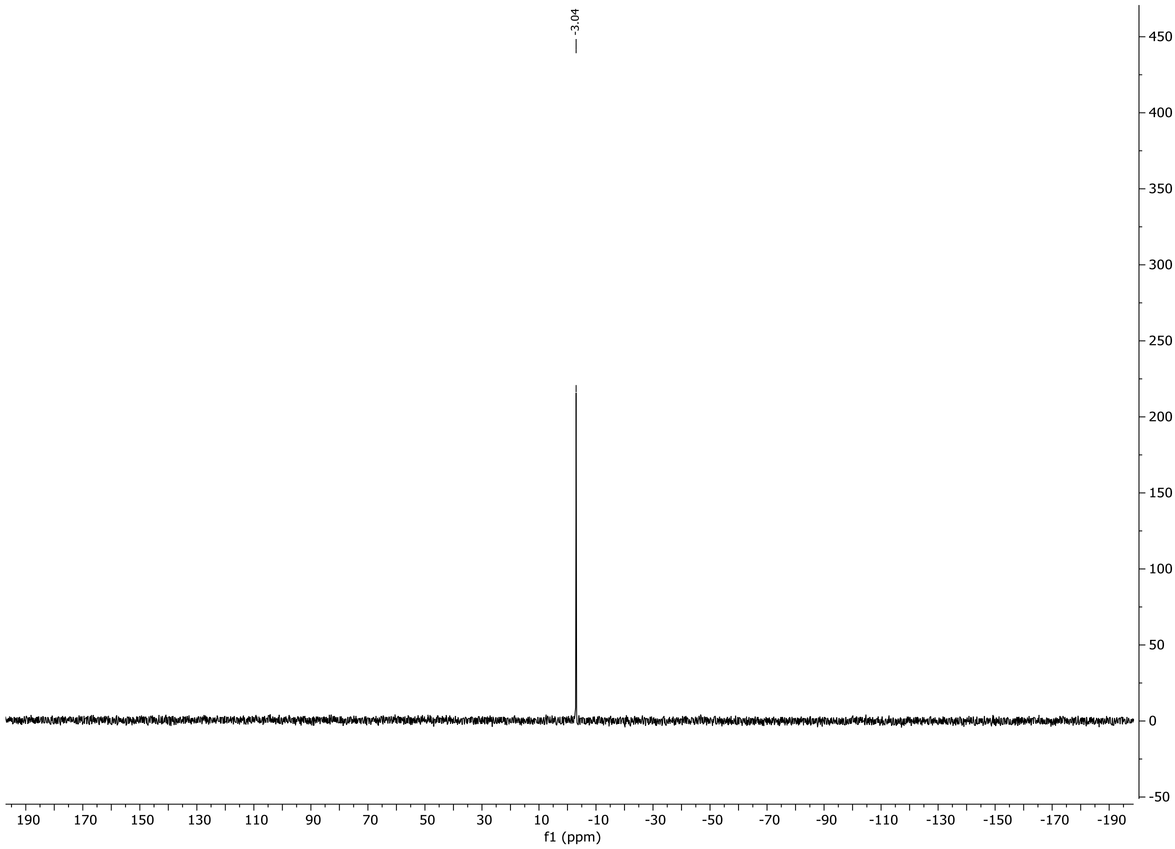

^{1}H NMR of compound **6b**

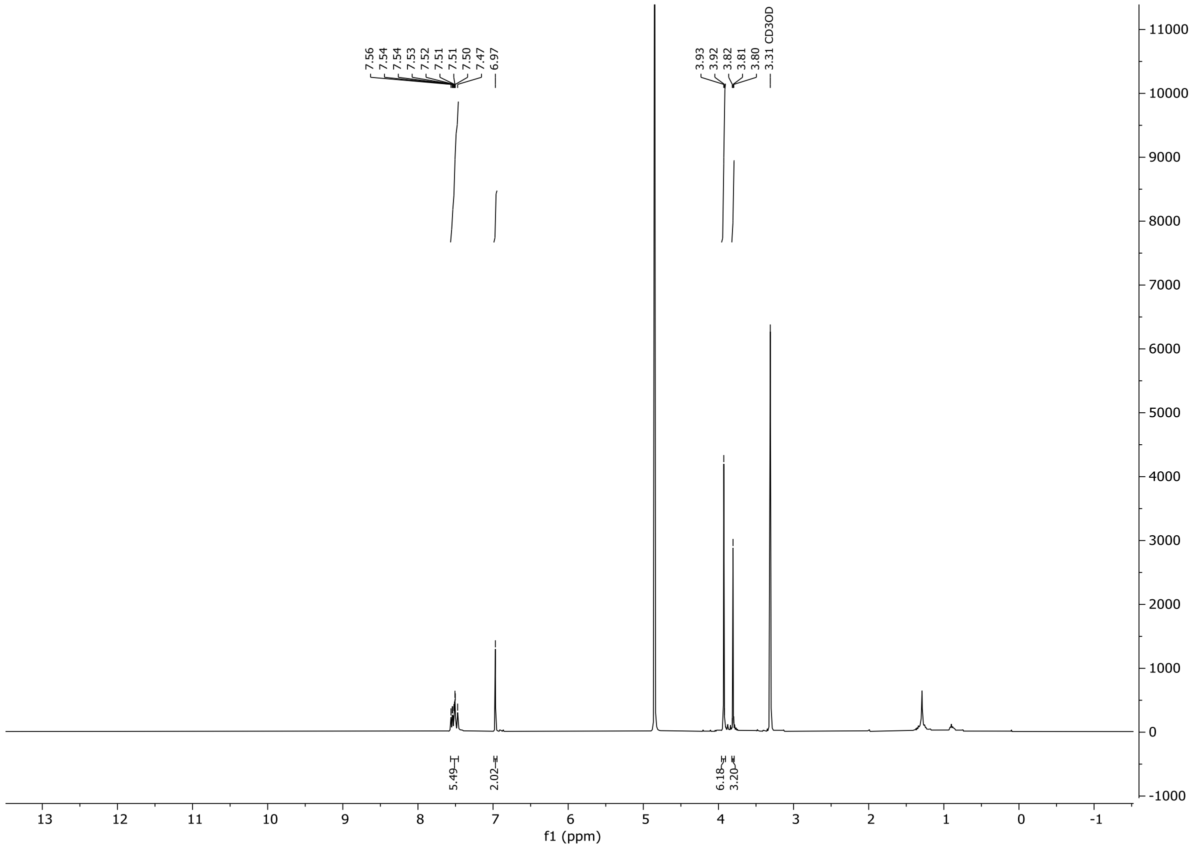

^{13}C NMR of compound **6b**

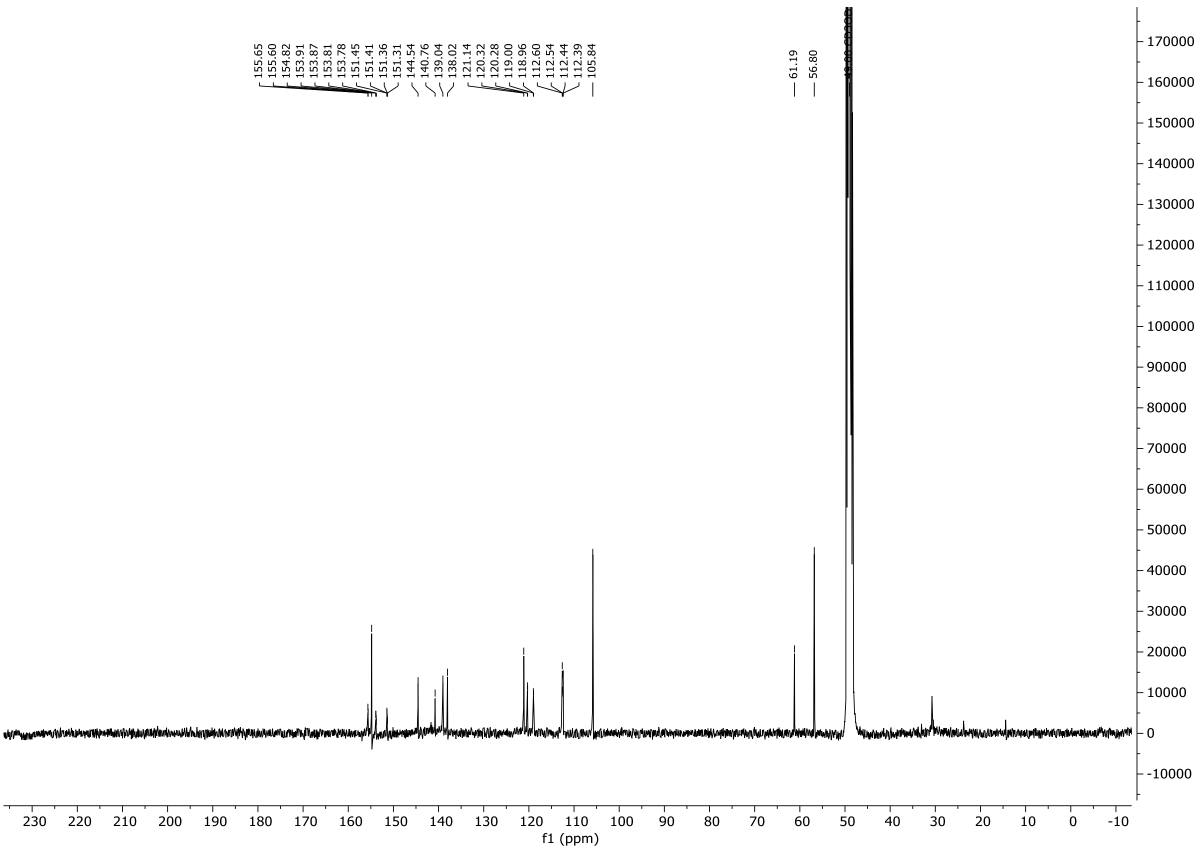

^{19}F NMR of compound **6b**

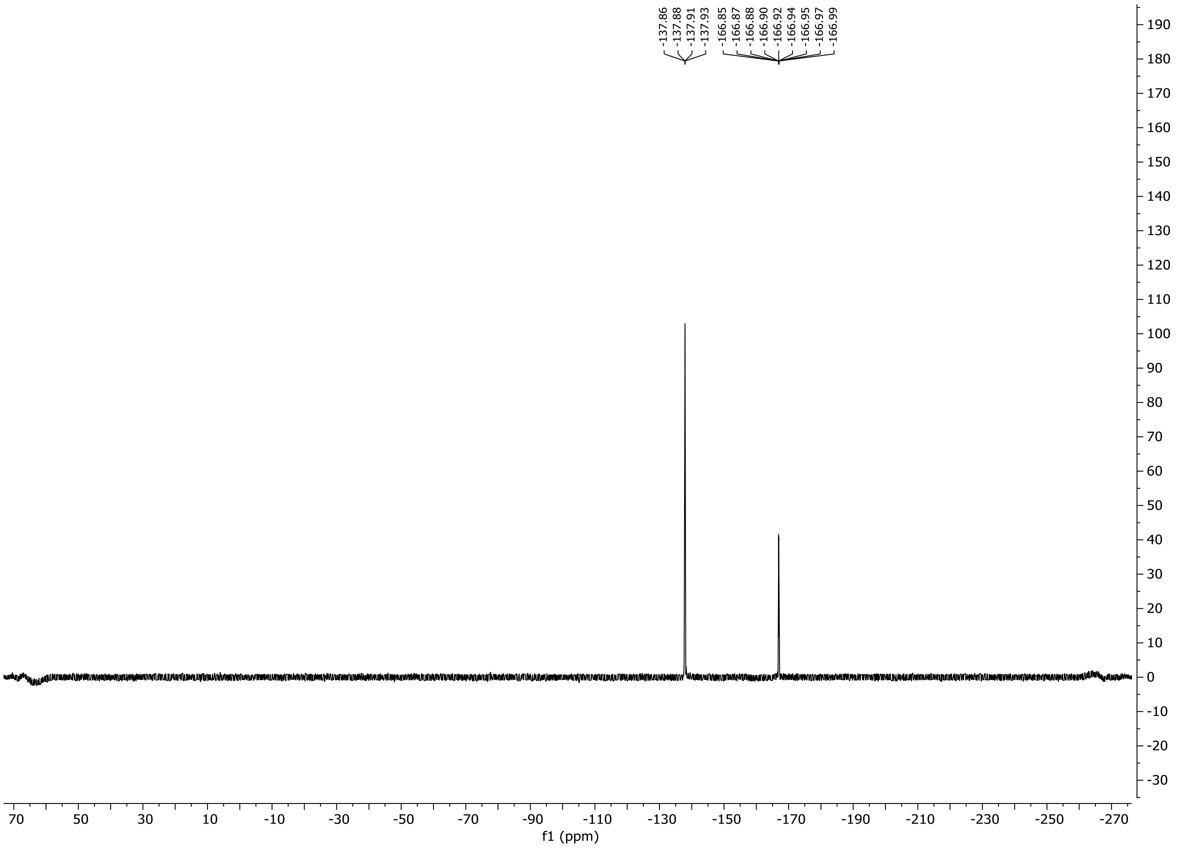

^{31}P NMR of compound **6b**

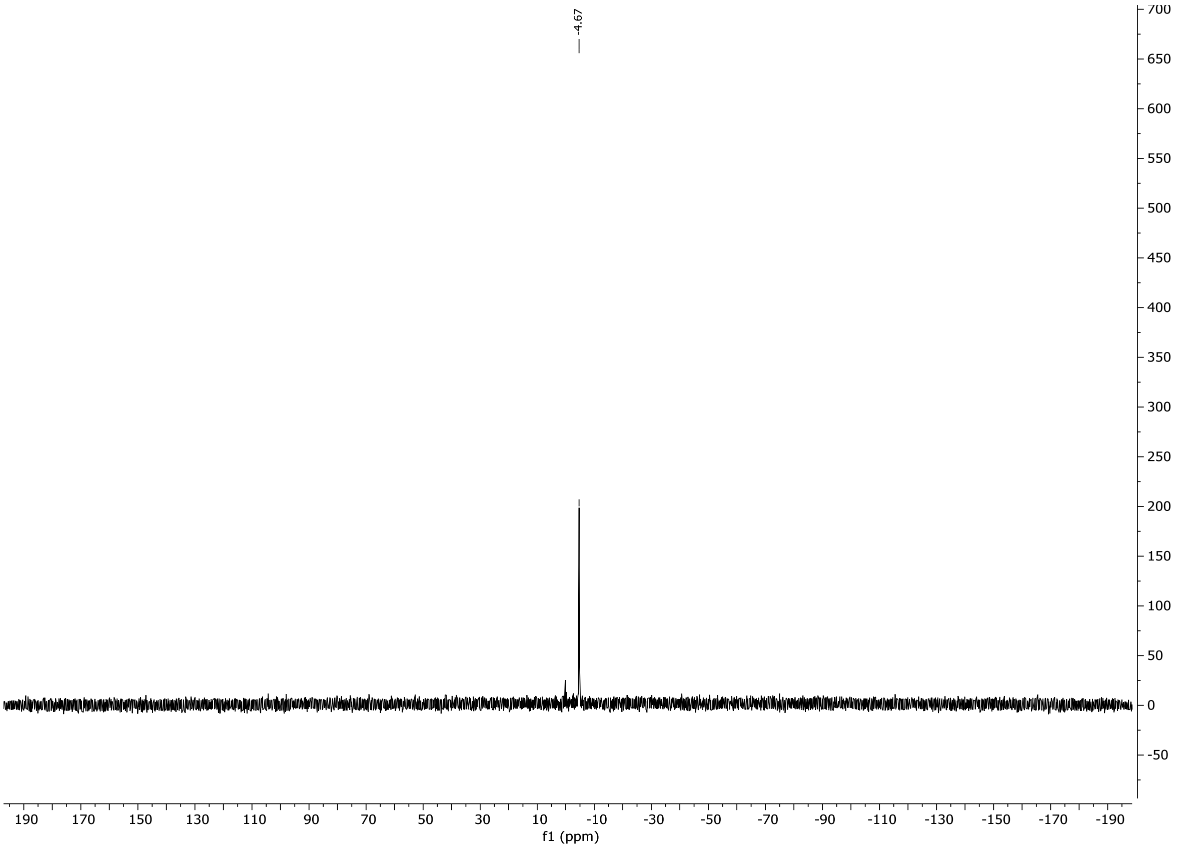

^{1}H NMR of compound **6c**

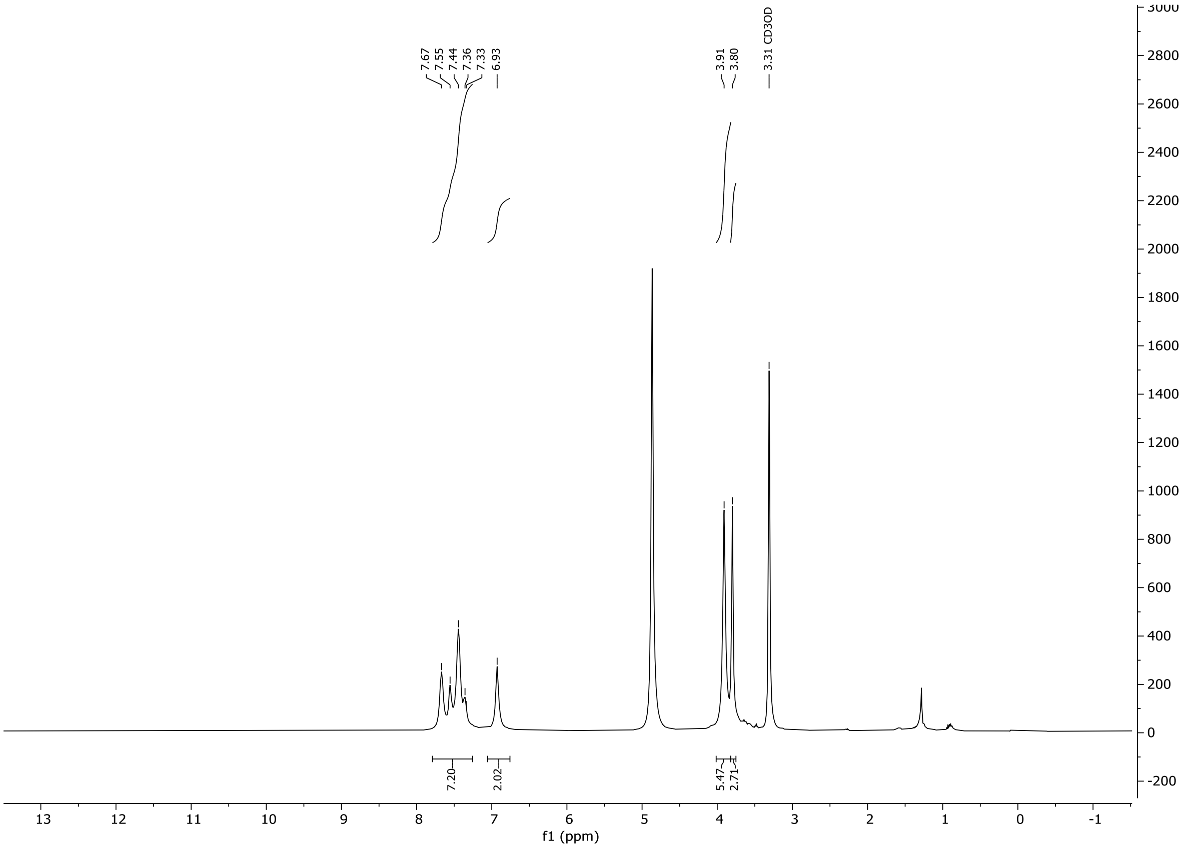

^{13}C NMR of compound **6c**

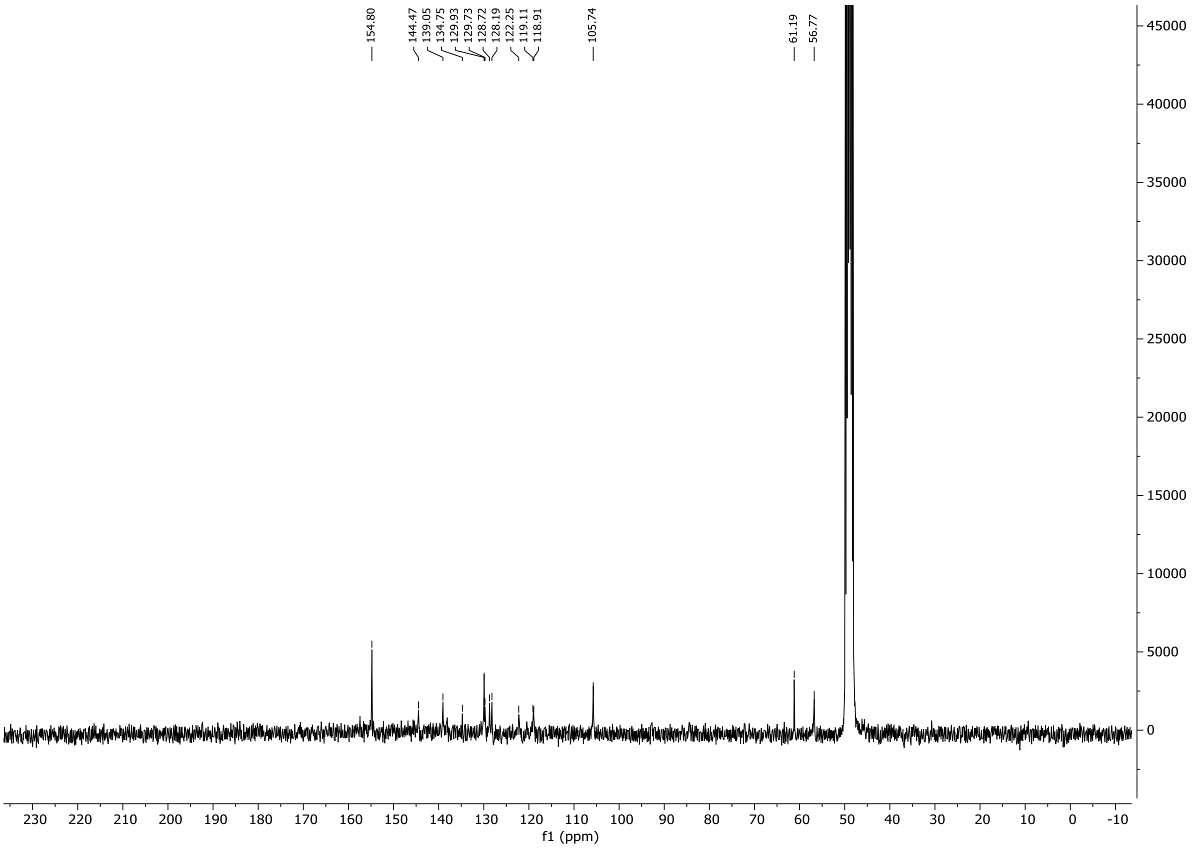

^{31}P NMR of compound **6c**

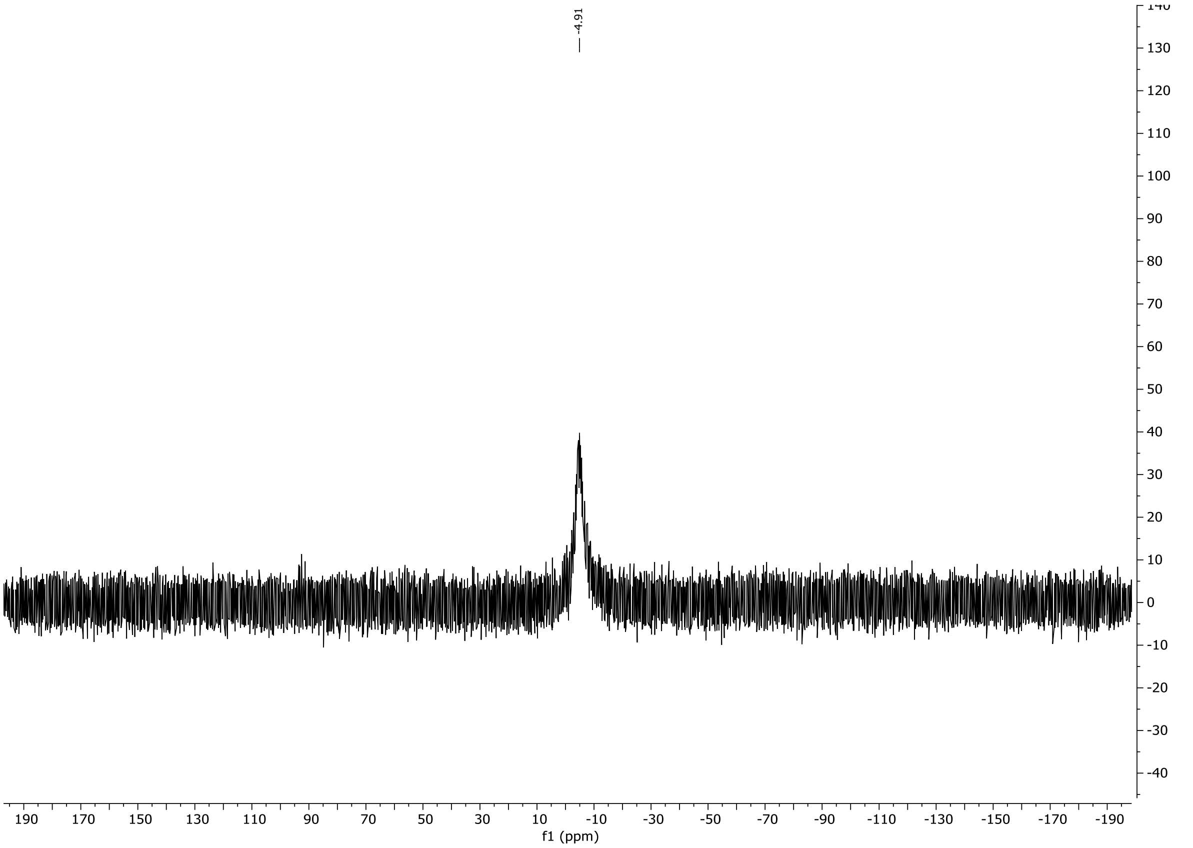

^{1}H NMR of compound **6d**

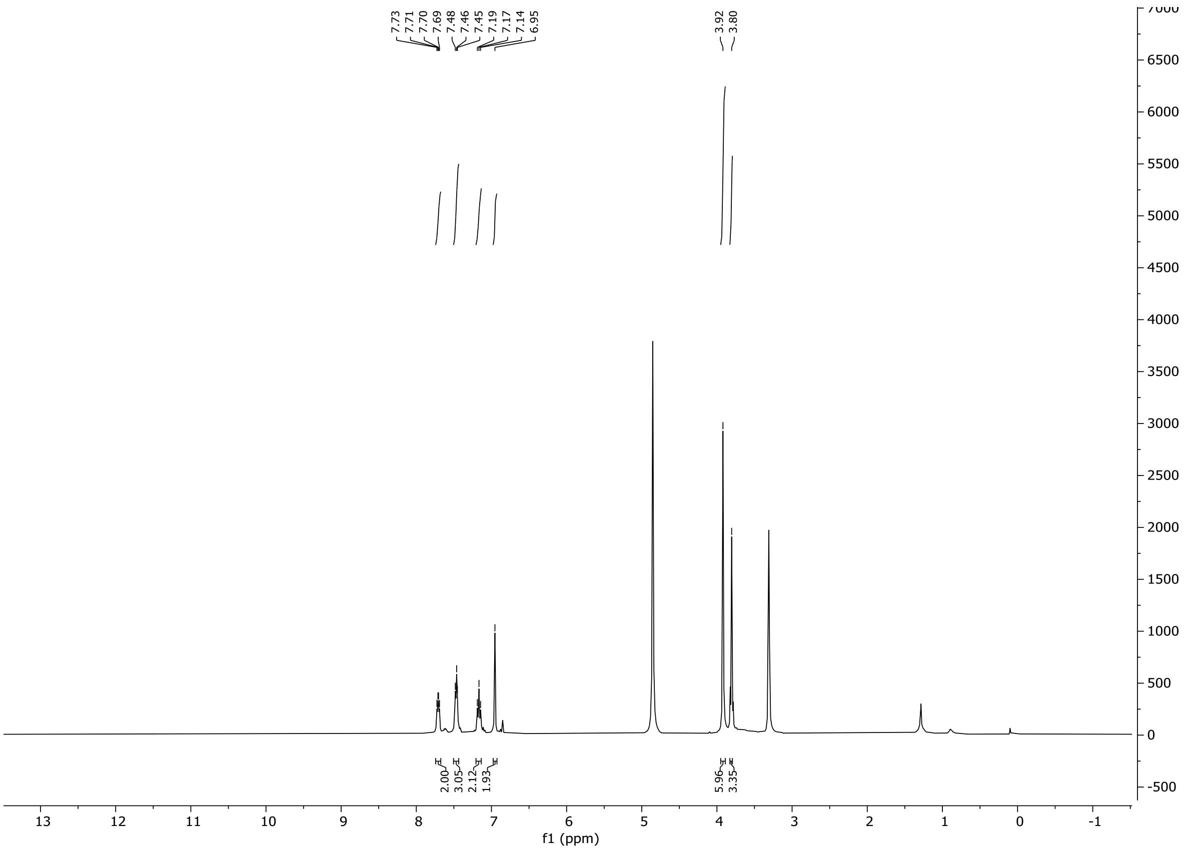

^{13}C NMR of compound **6d**

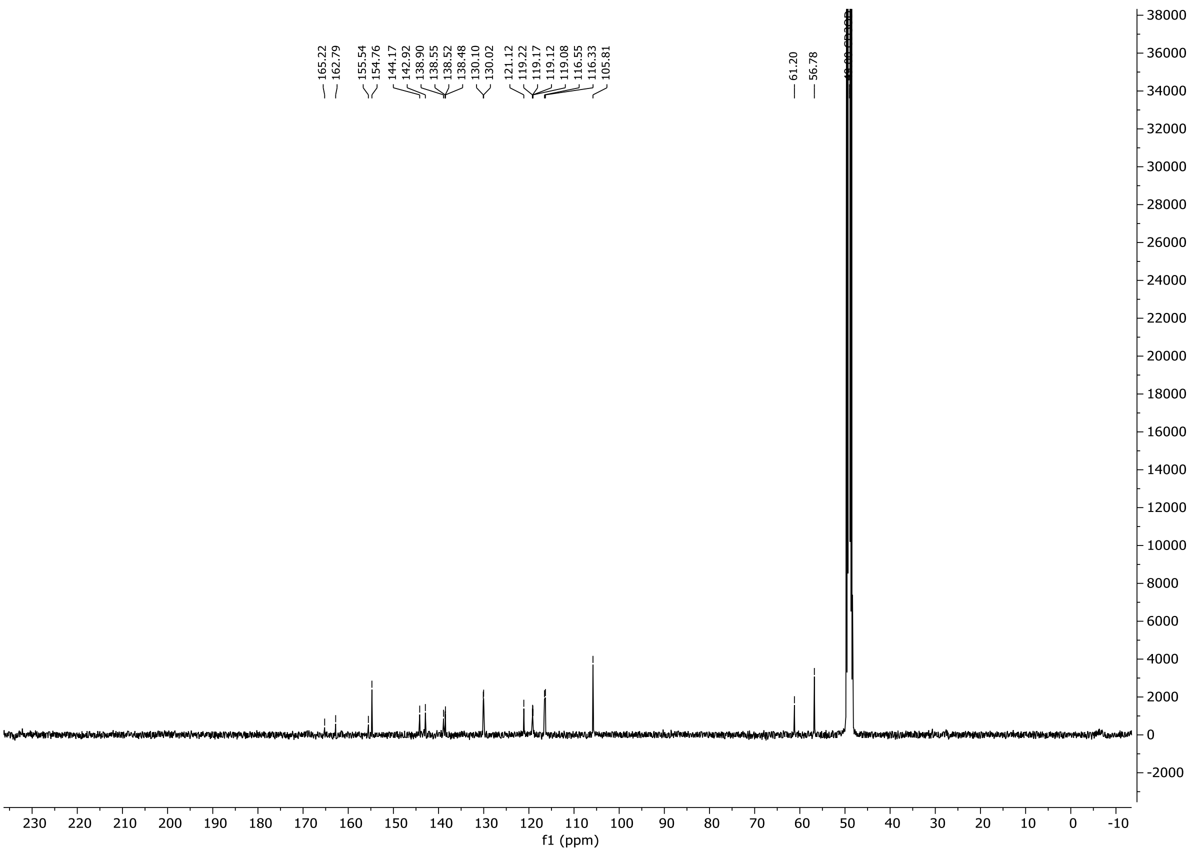

^{19}F NMR of compound **6d**

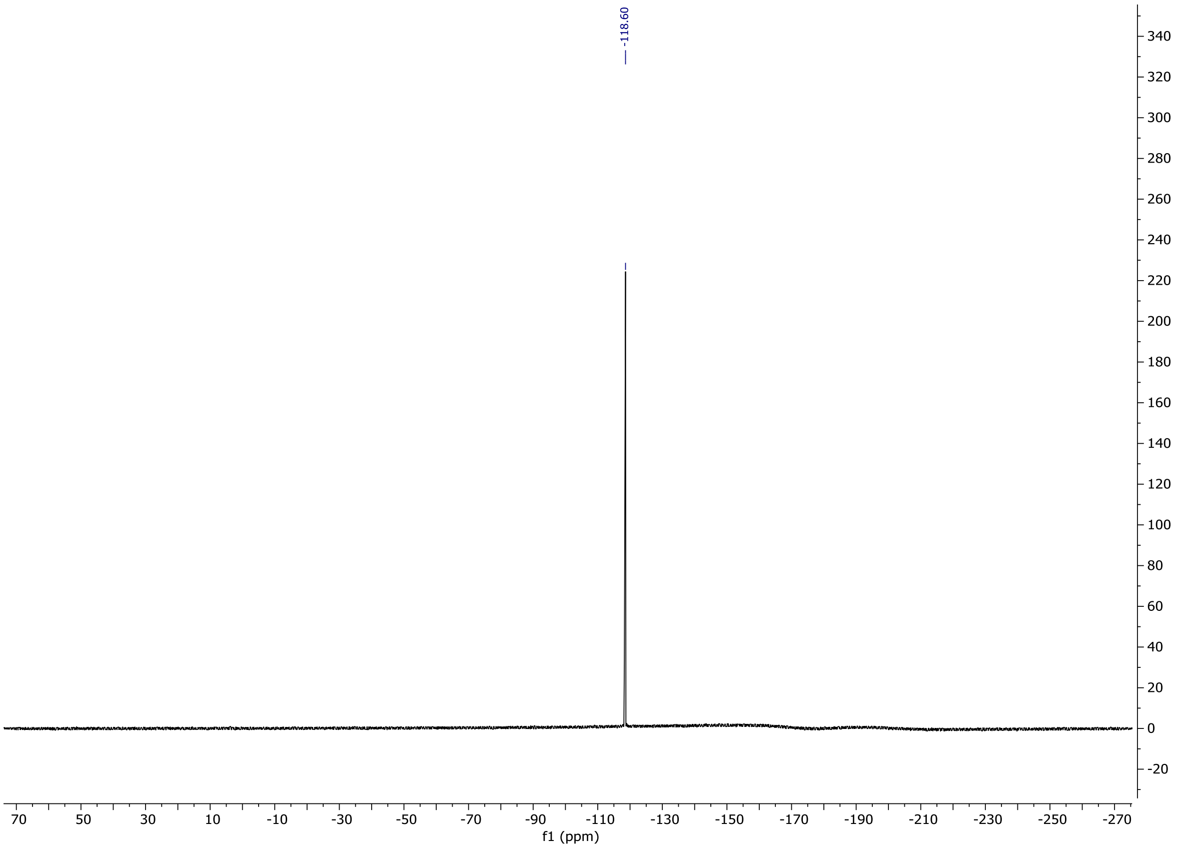

^{31}P NMR of compound **6d**

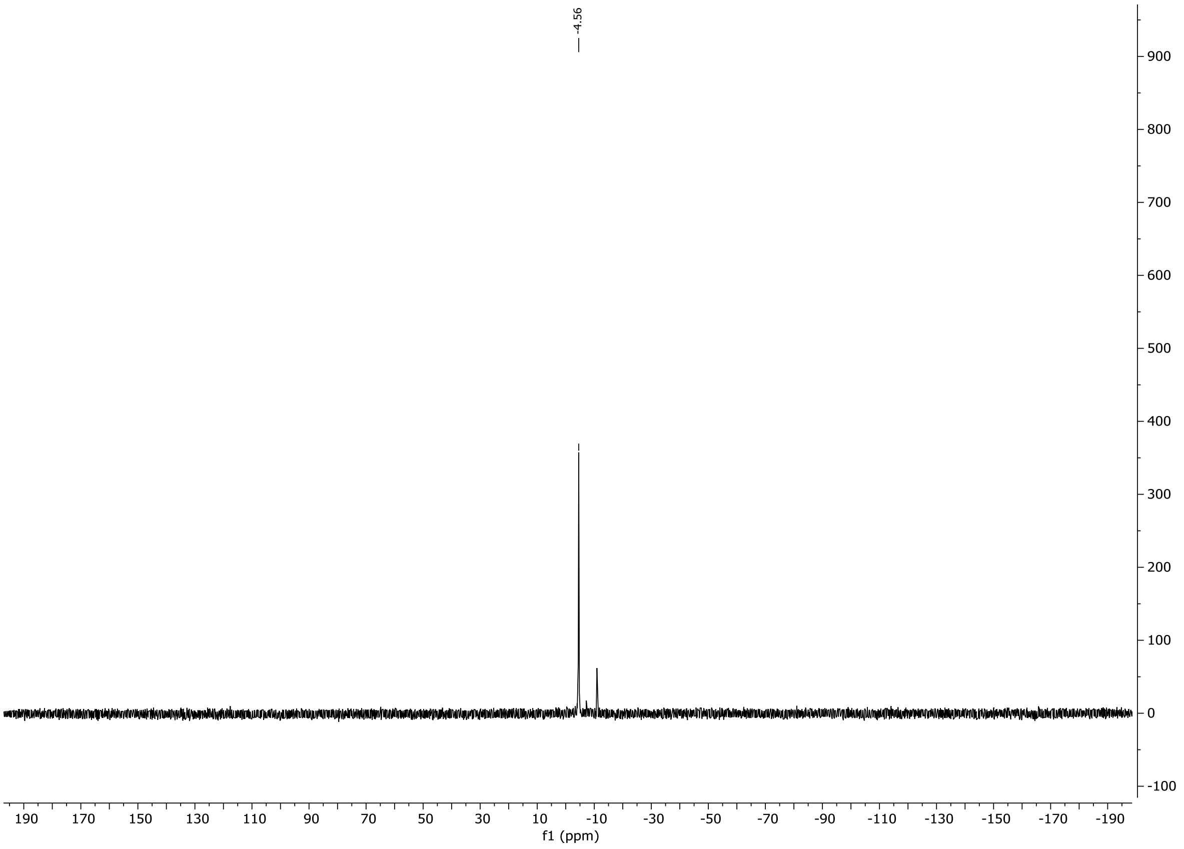

^{1}H NMR of compound **6e**

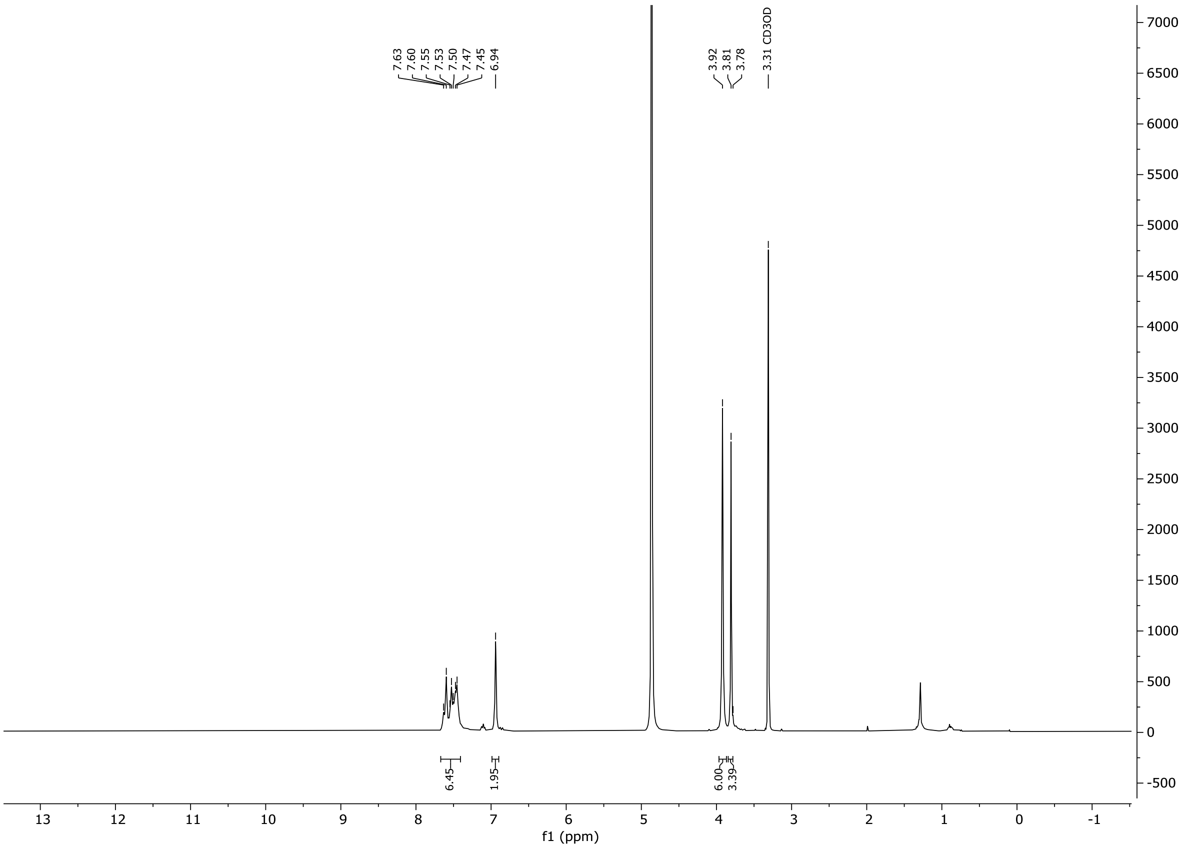

^{13}C NMR of compound **6e**

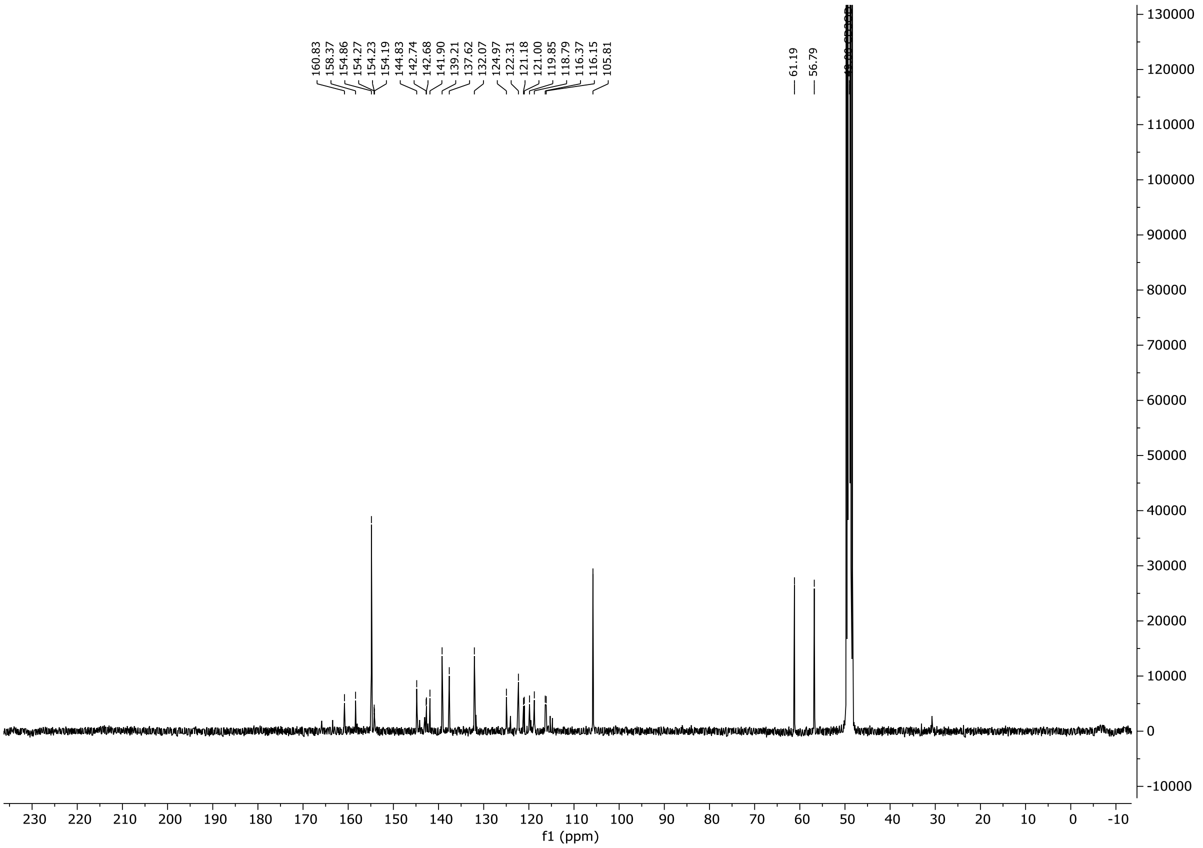

^{31}P NMR of compound **6e**

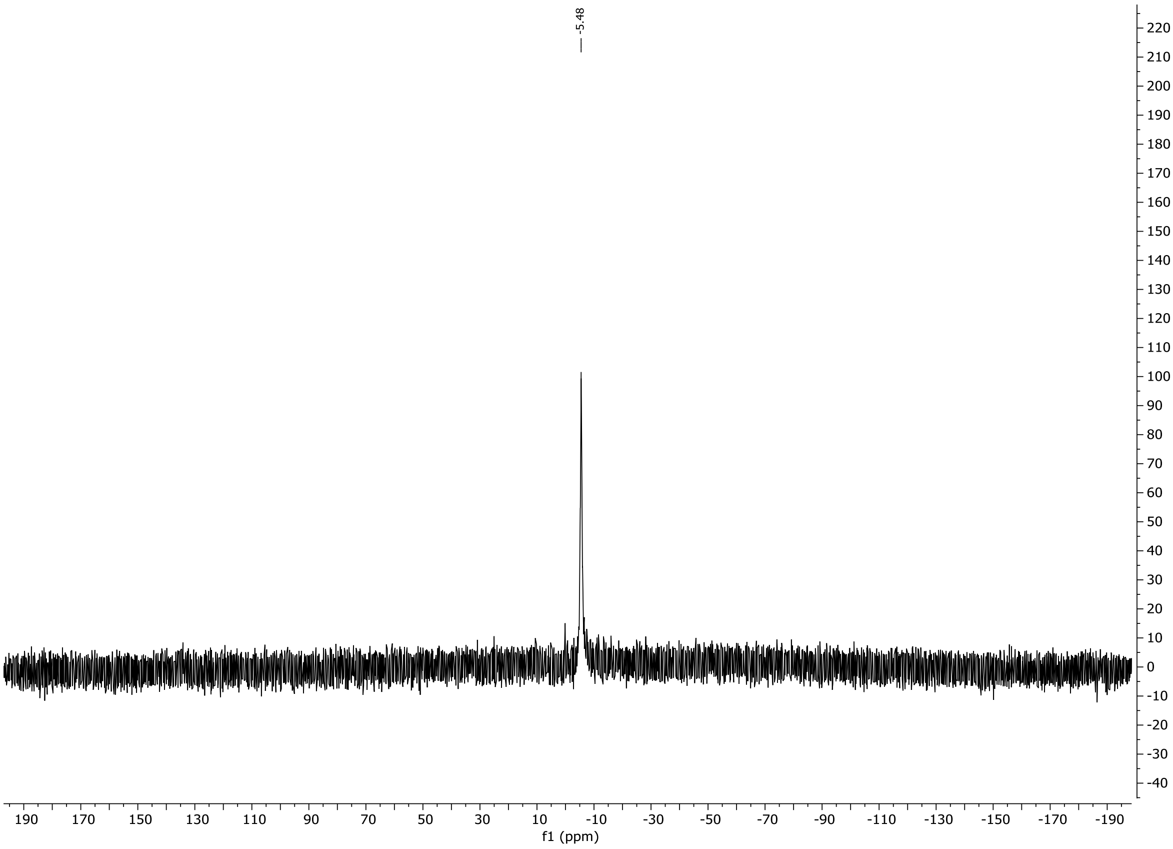

^{1}H NMR of compound **6f**

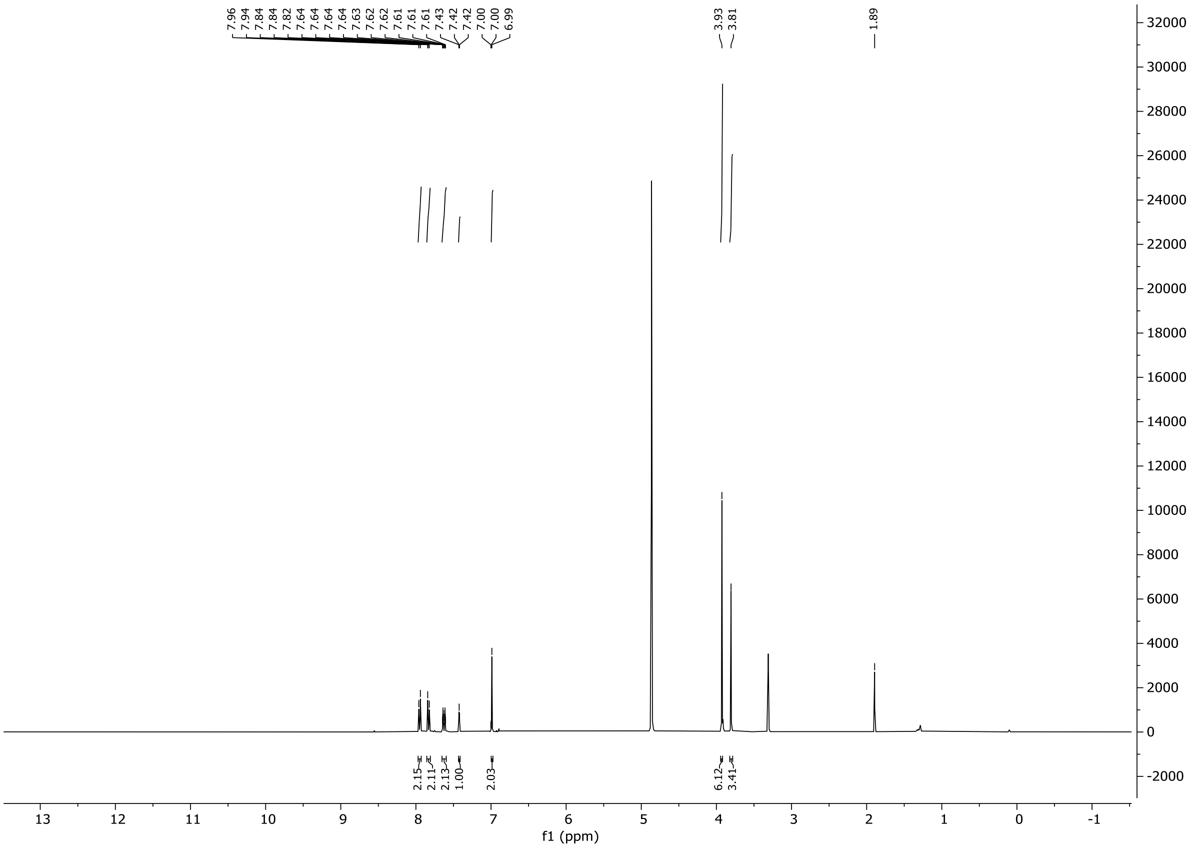

^{13}C NMR of compound **6f**

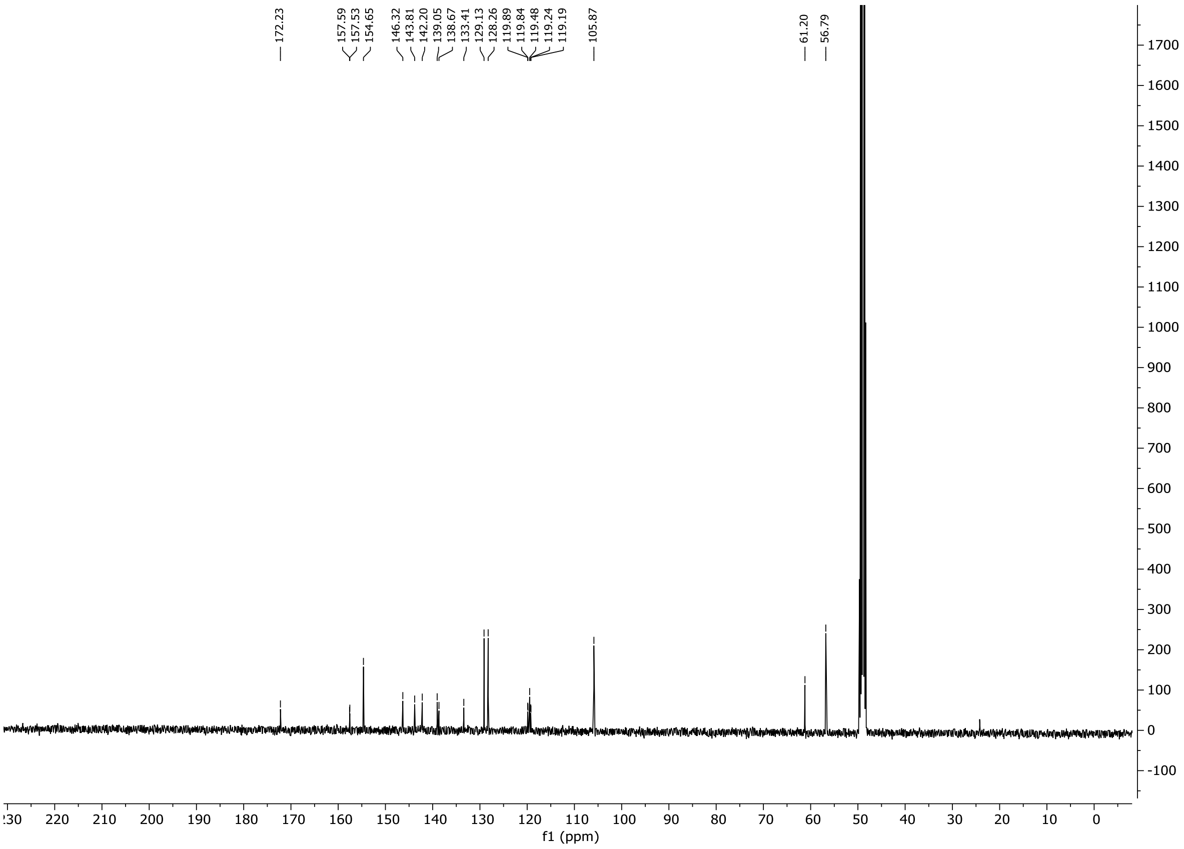

^{31}P NMR of compound **6f**

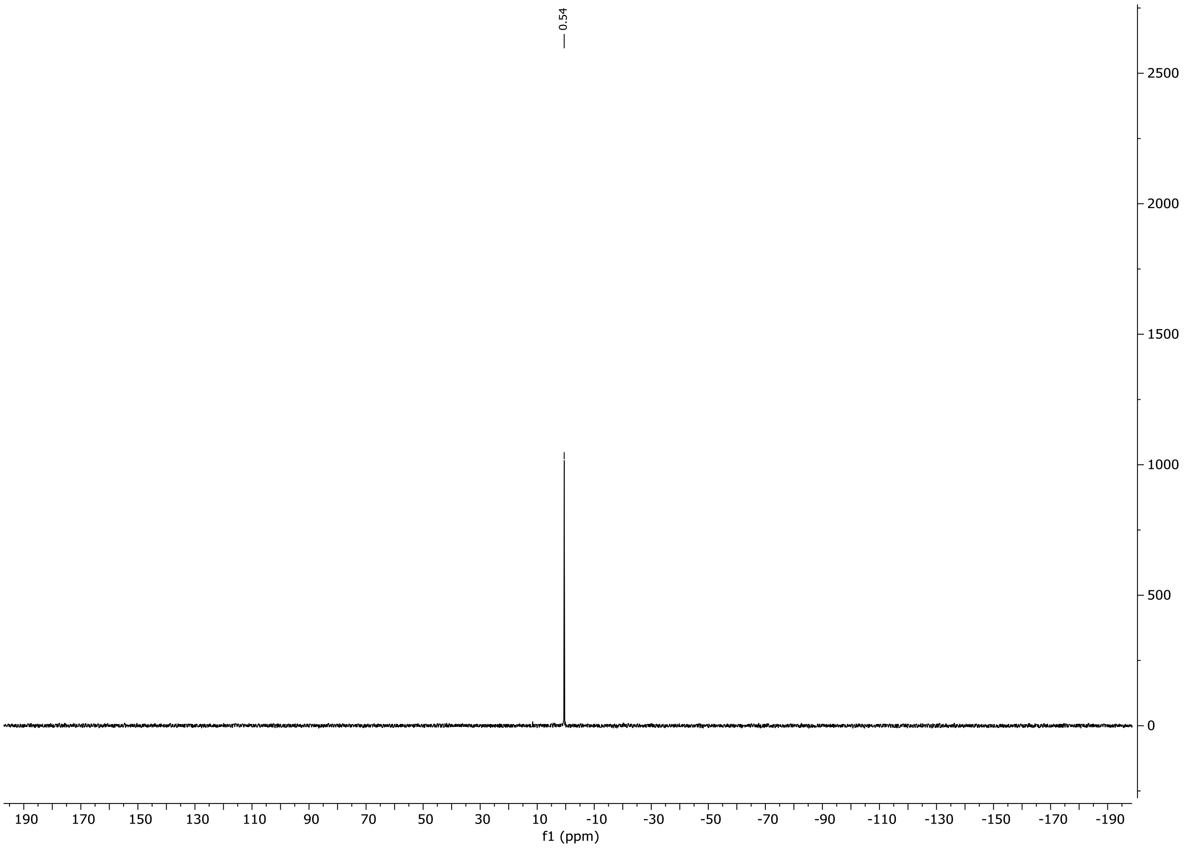

^{1}H NMR of compound **6g**

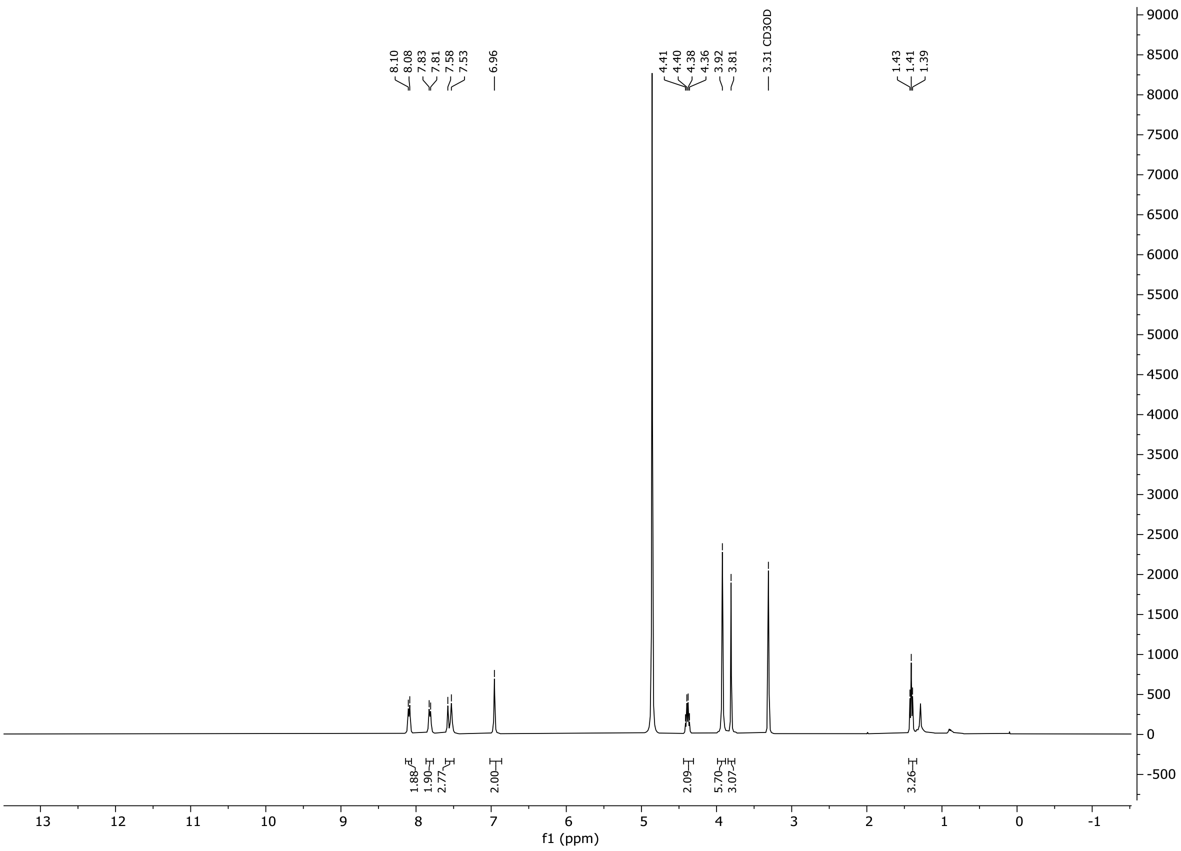

^{13}C NMR of compound **6g**

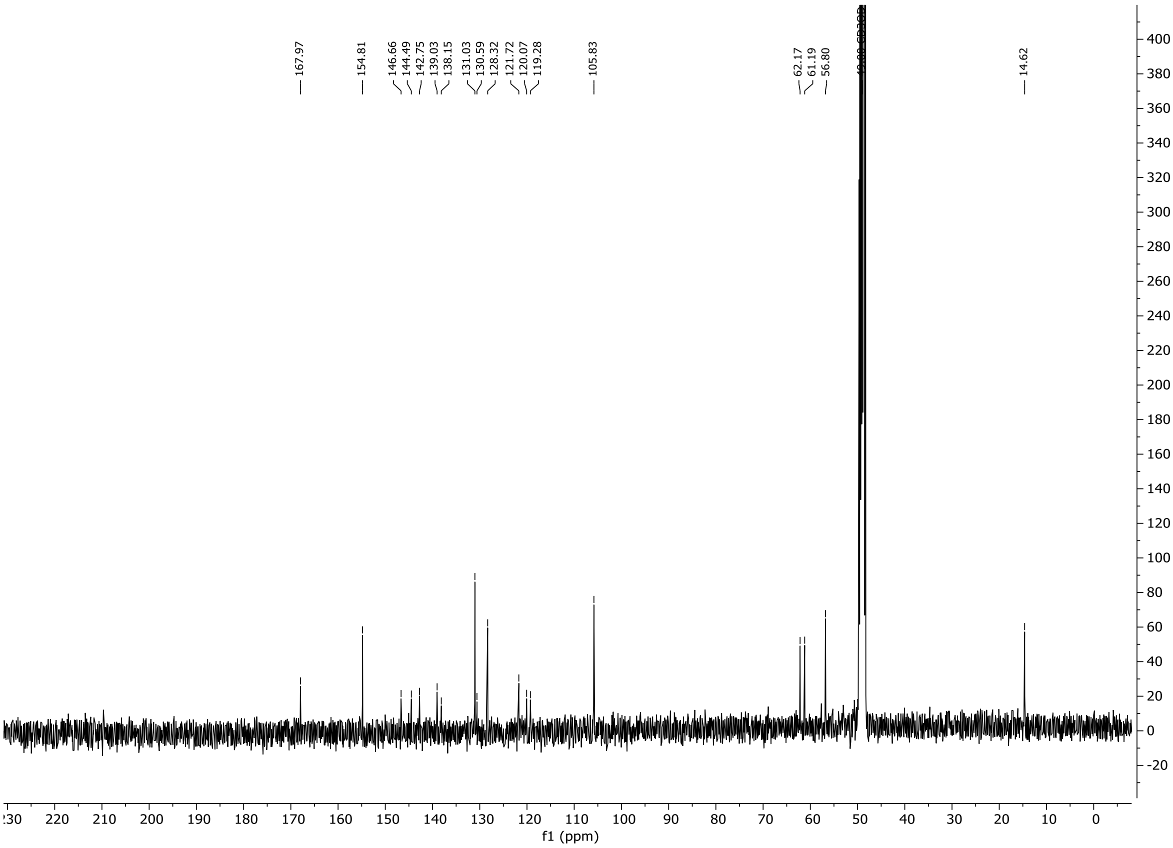

^{31}P NMR of compound **6g**

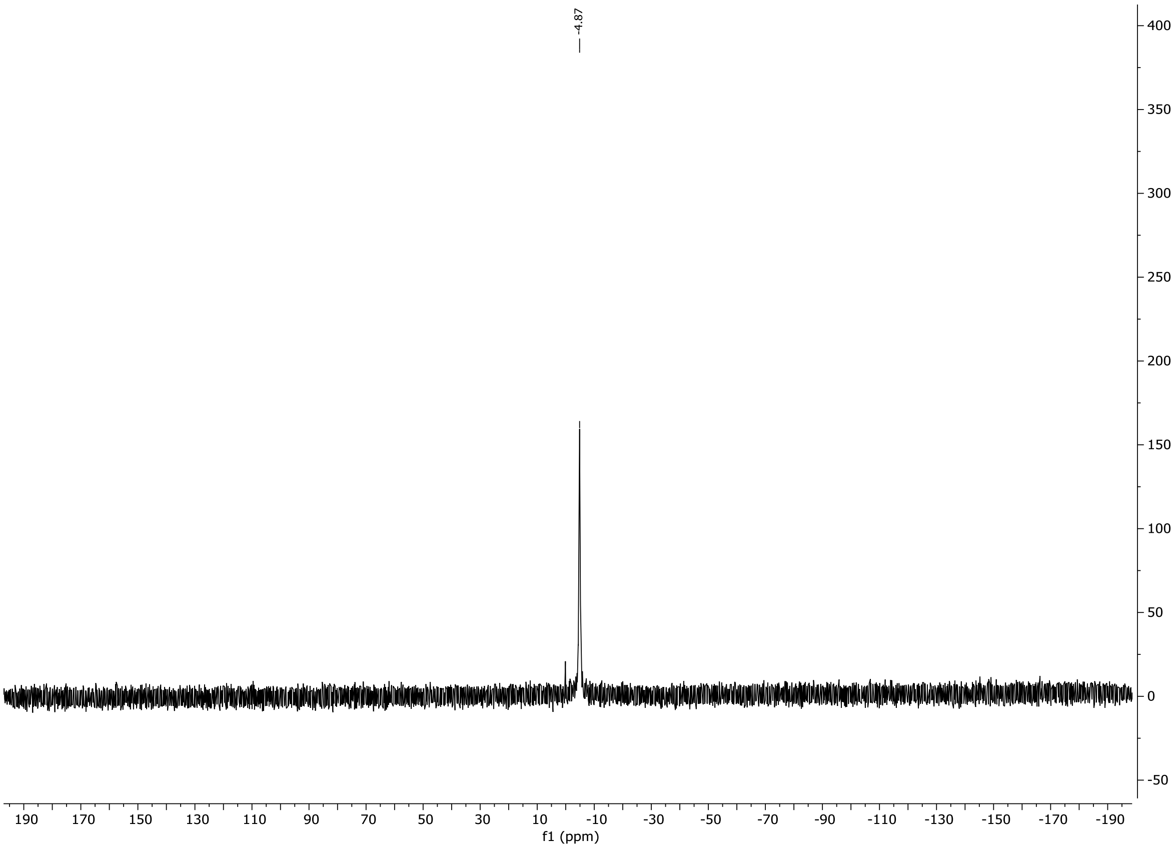

^{1}H NMR of compound **6h**

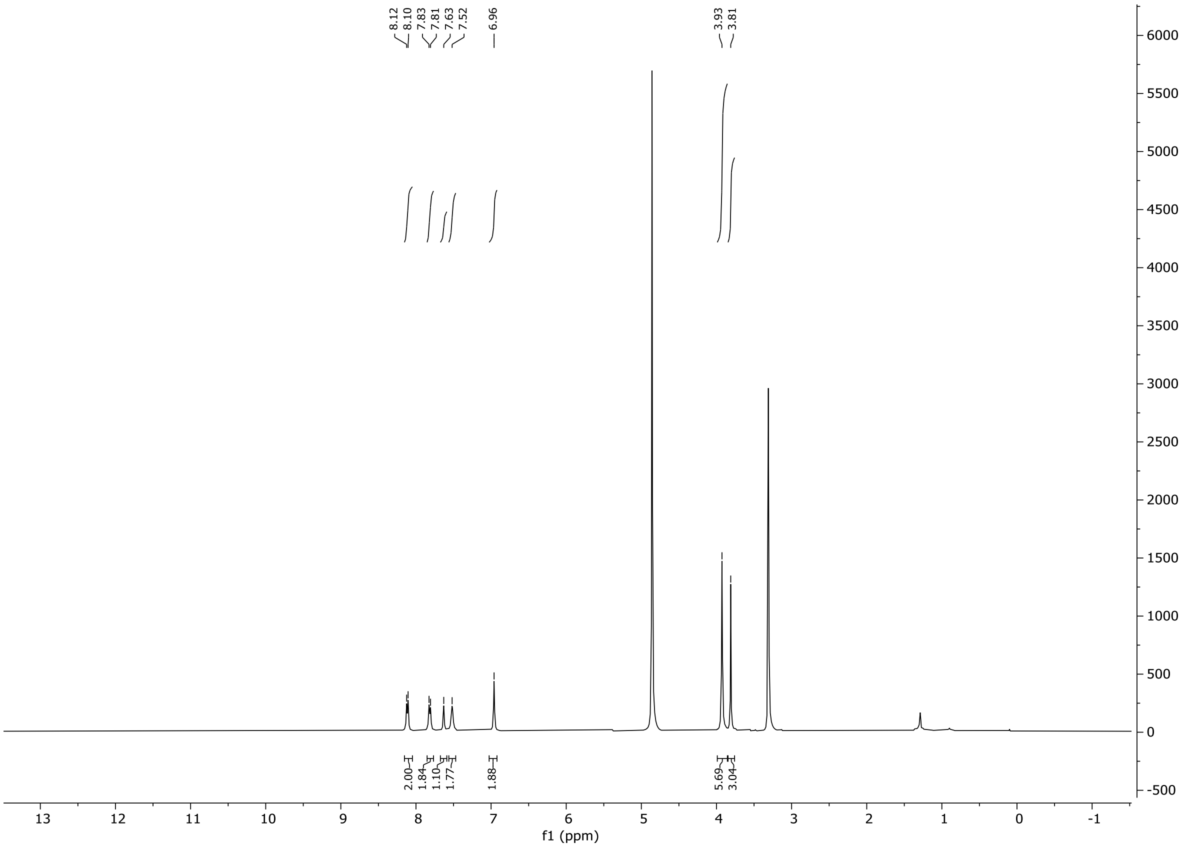

^{13}C NMR of compound **6h**

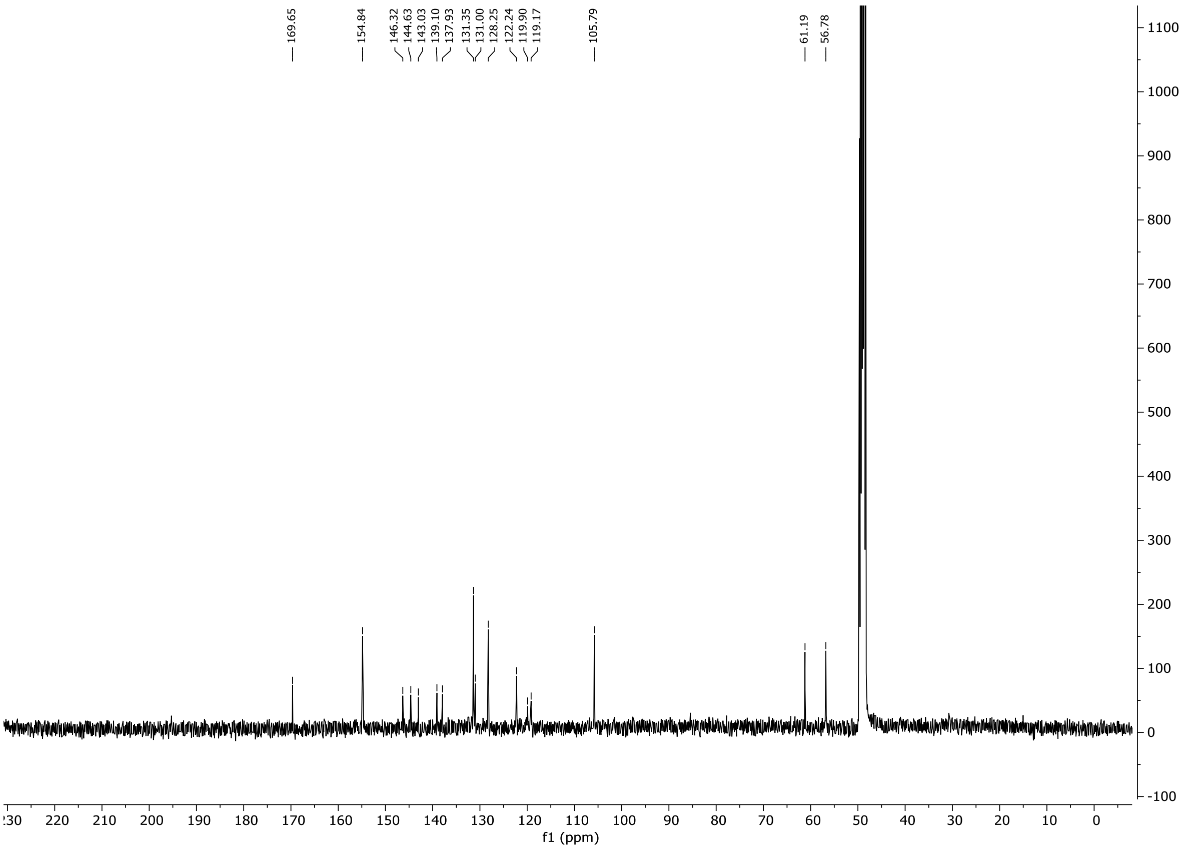

^{31}P NMR of compound **6h**

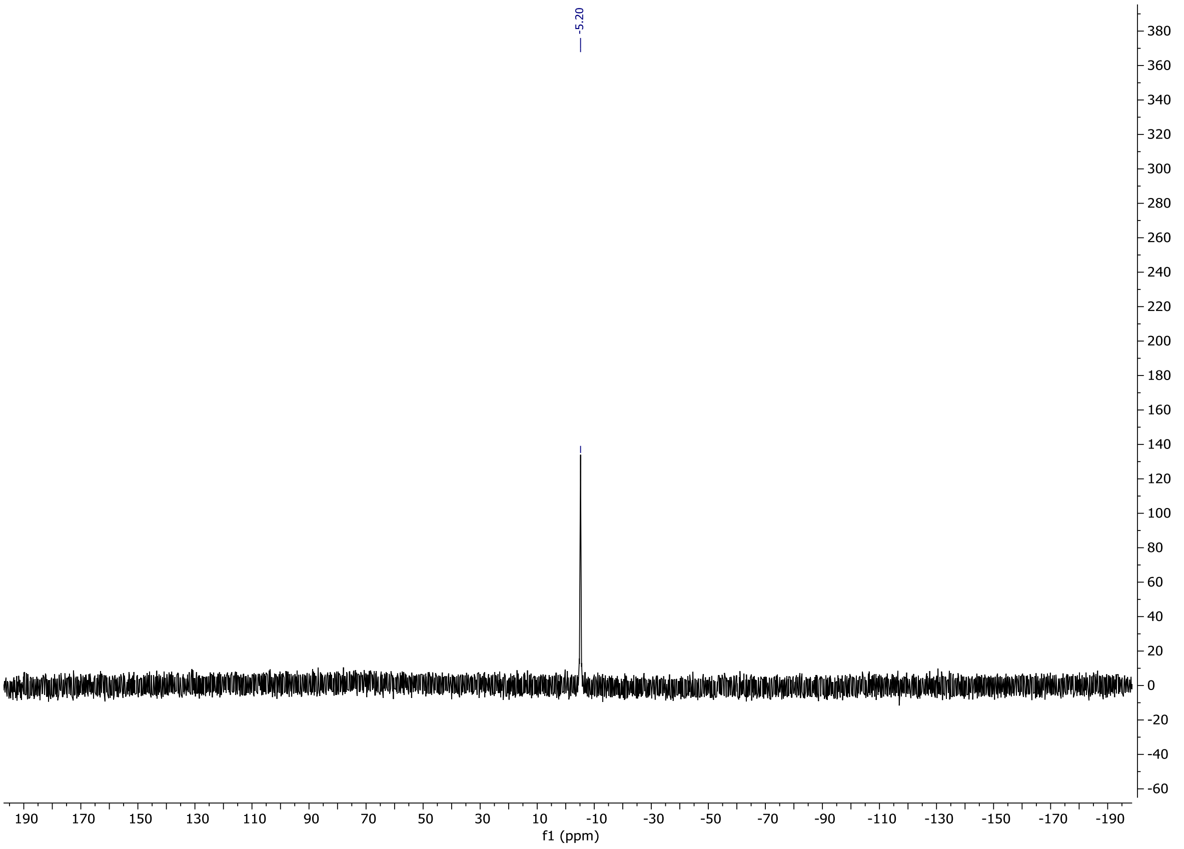

^{1}H NMR of compound **6i**

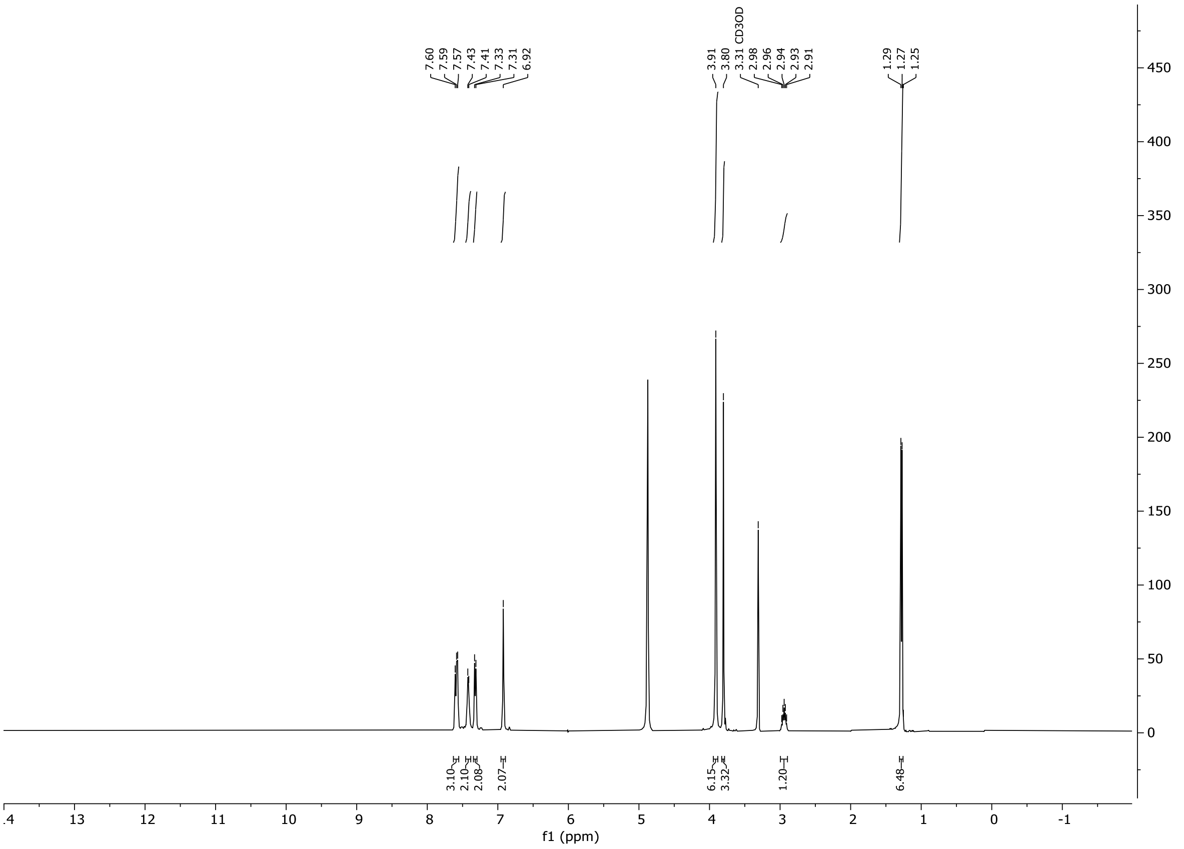

^{13}C NMR of compound **6i**

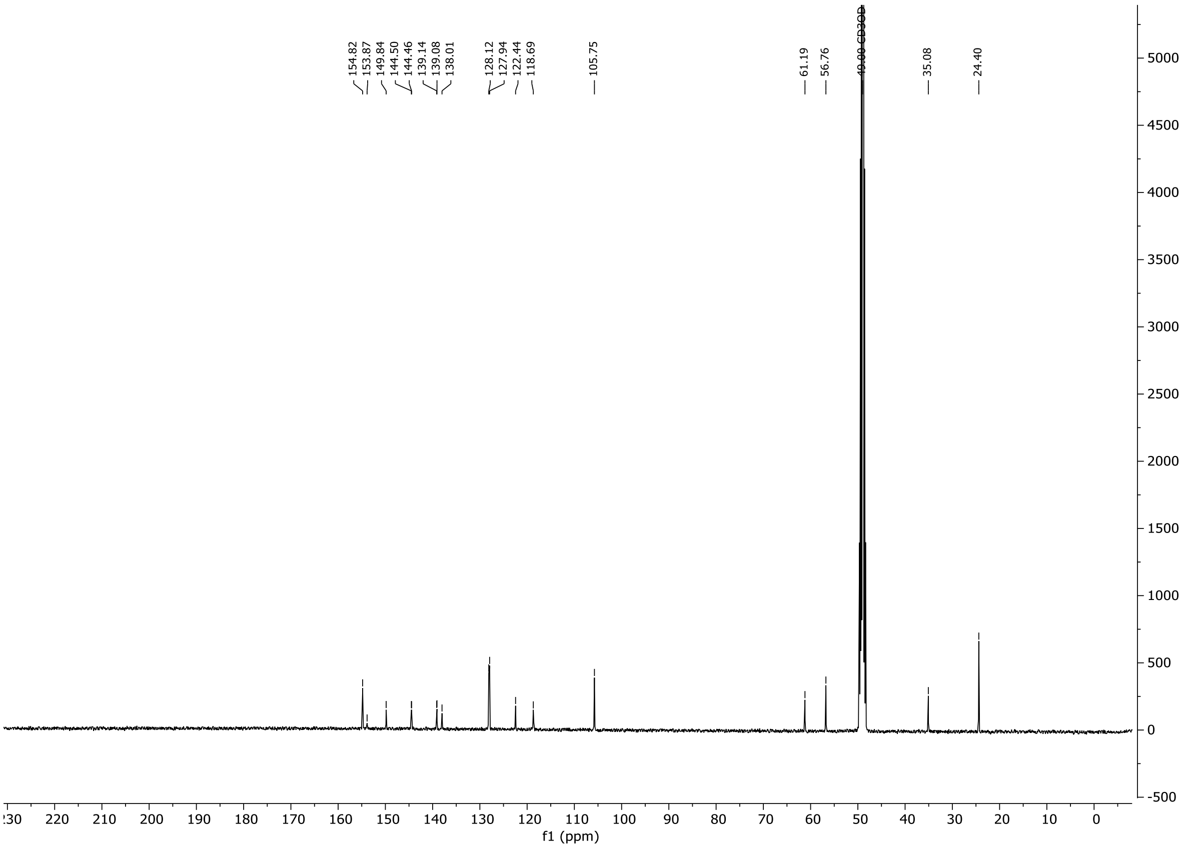

^{31}P NMR of compound **6i**

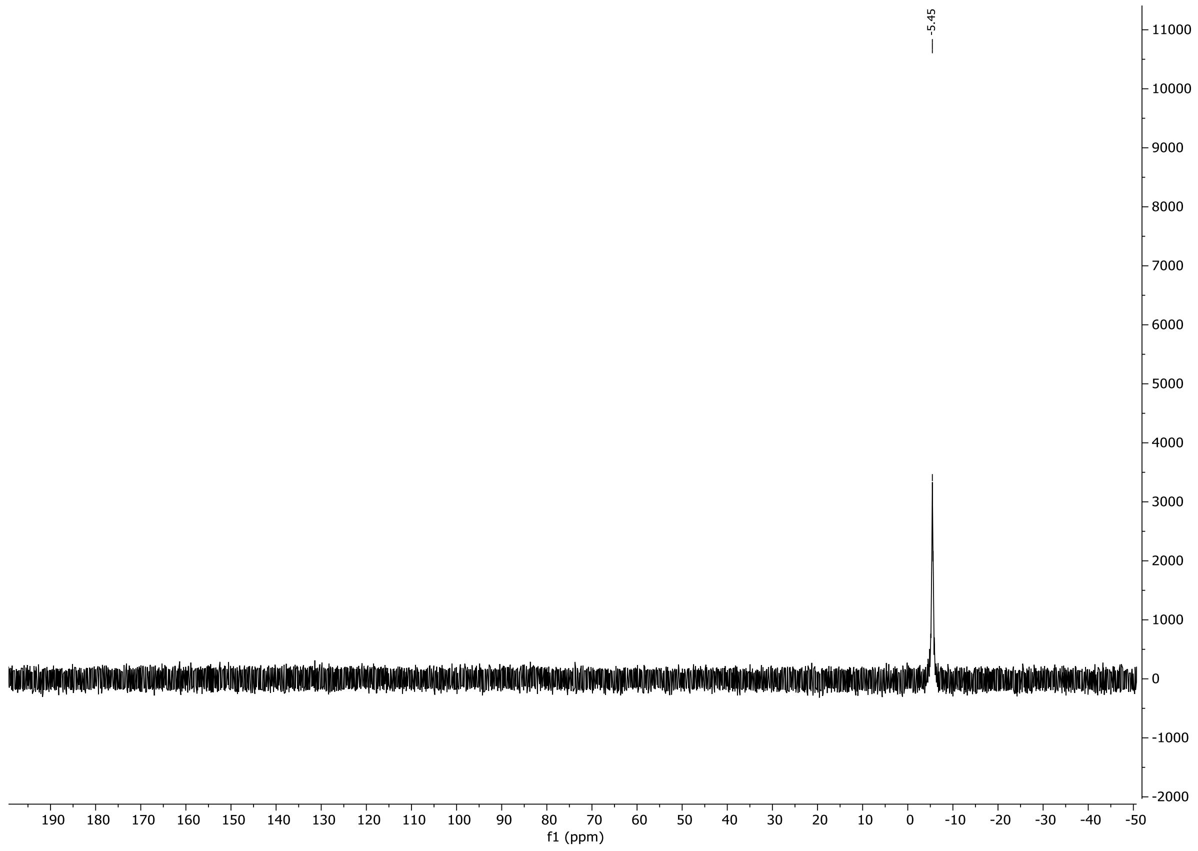

^{1}H NMR of compound **6j**

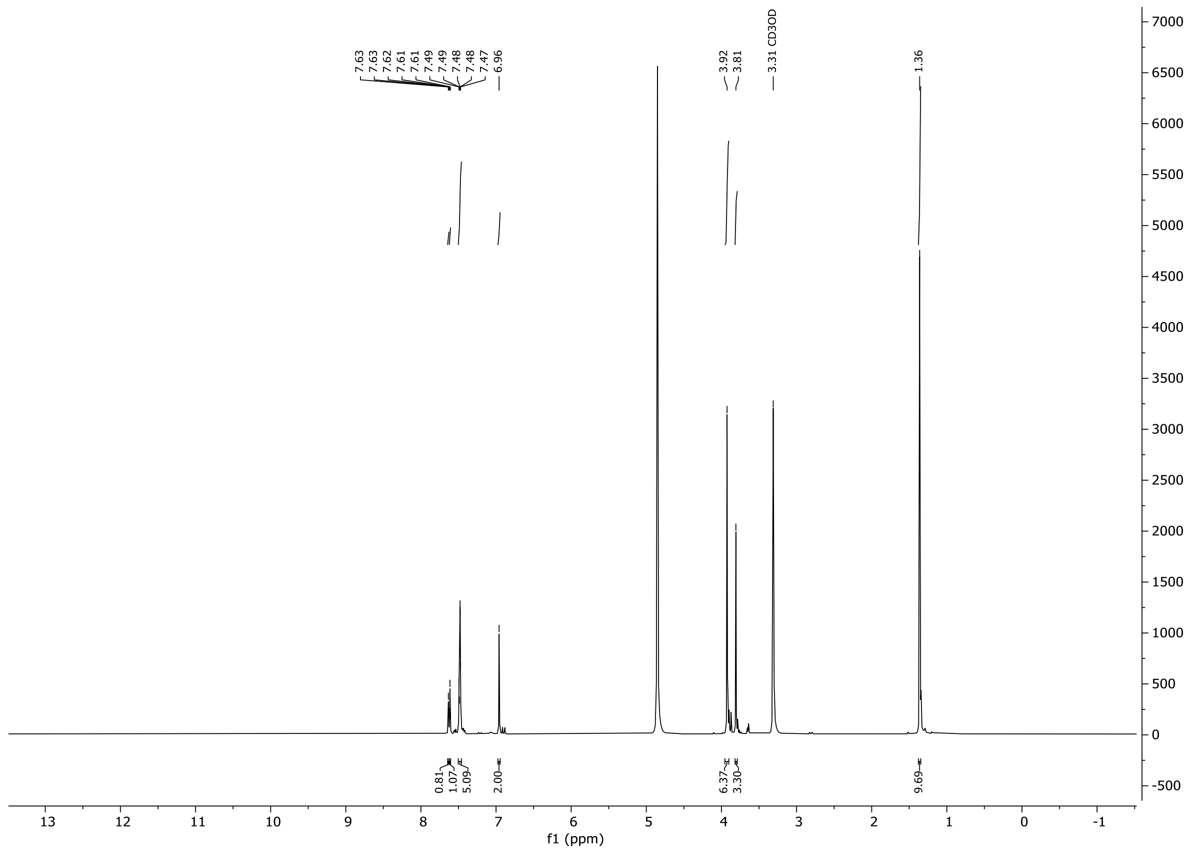

^{13}C NMR of compound **6j**

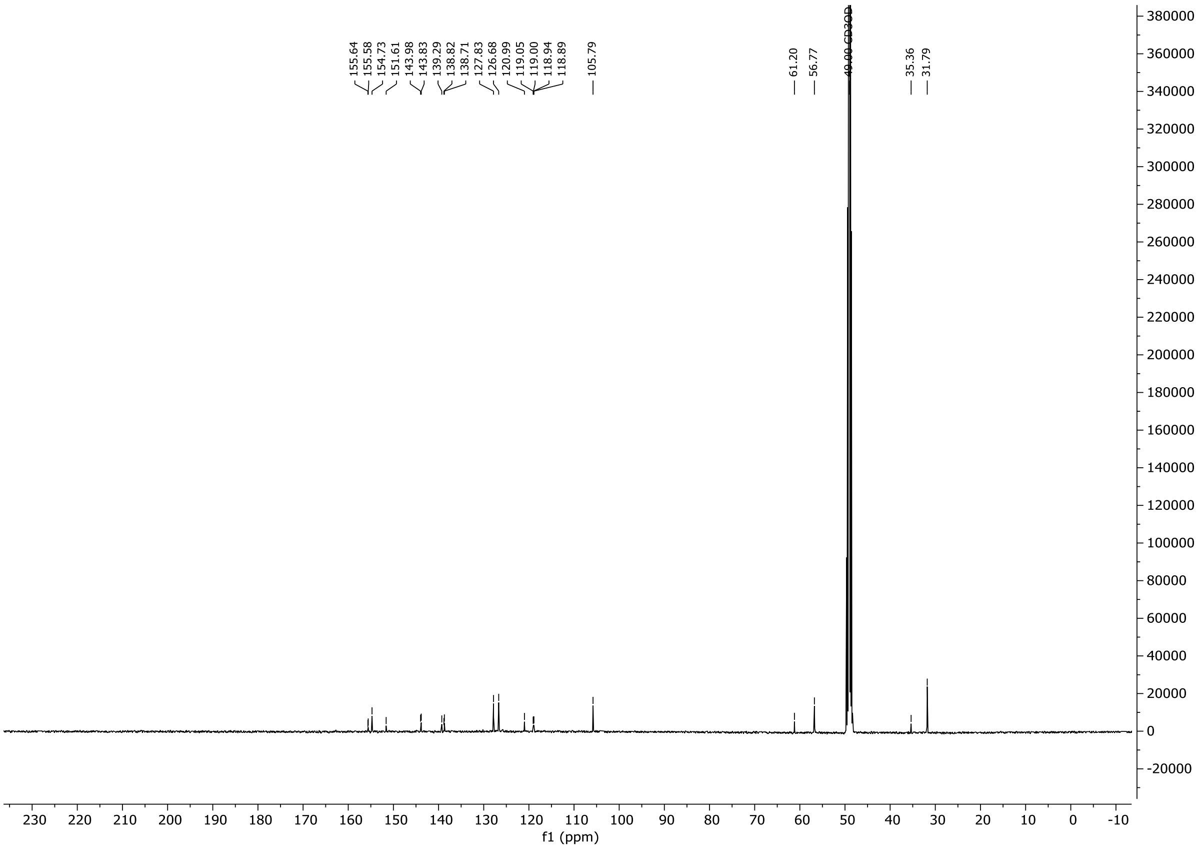

^{31}P NMR of compound **6j**

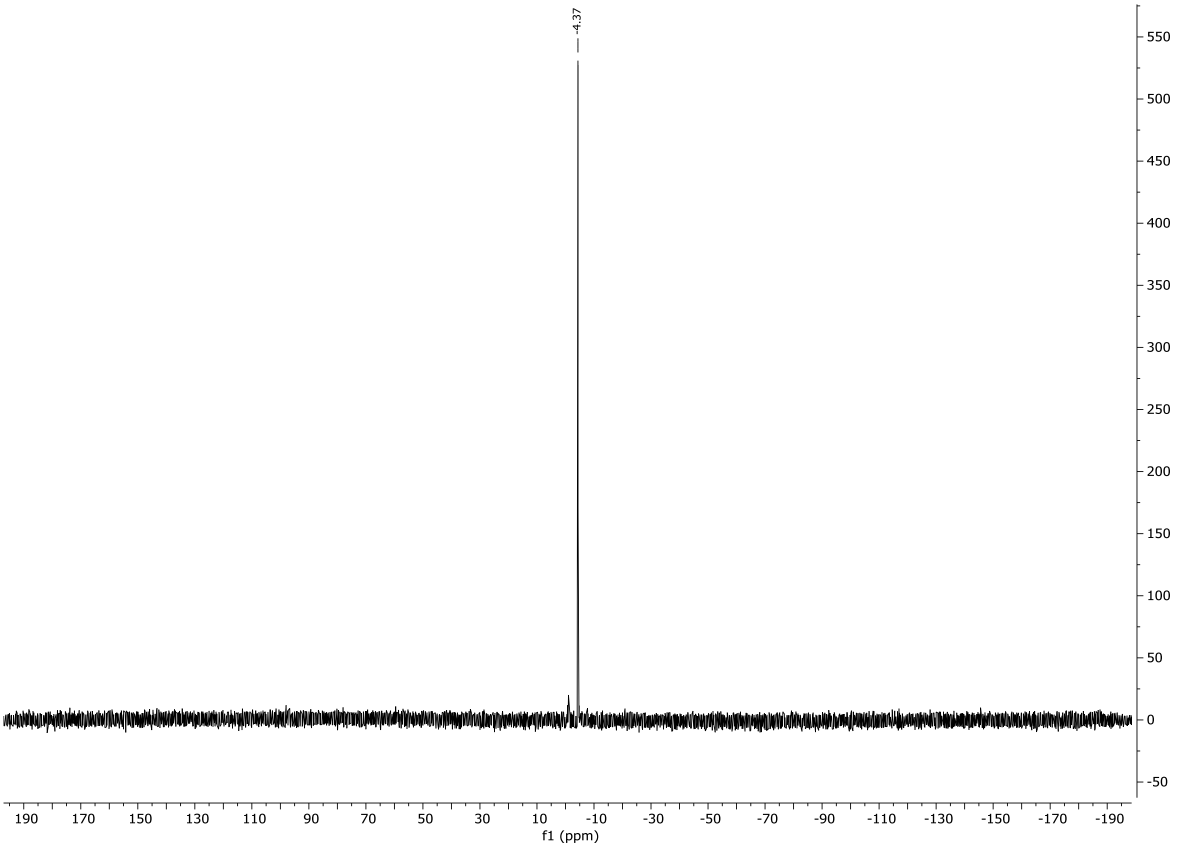

^{1}H NMR of compound **12**

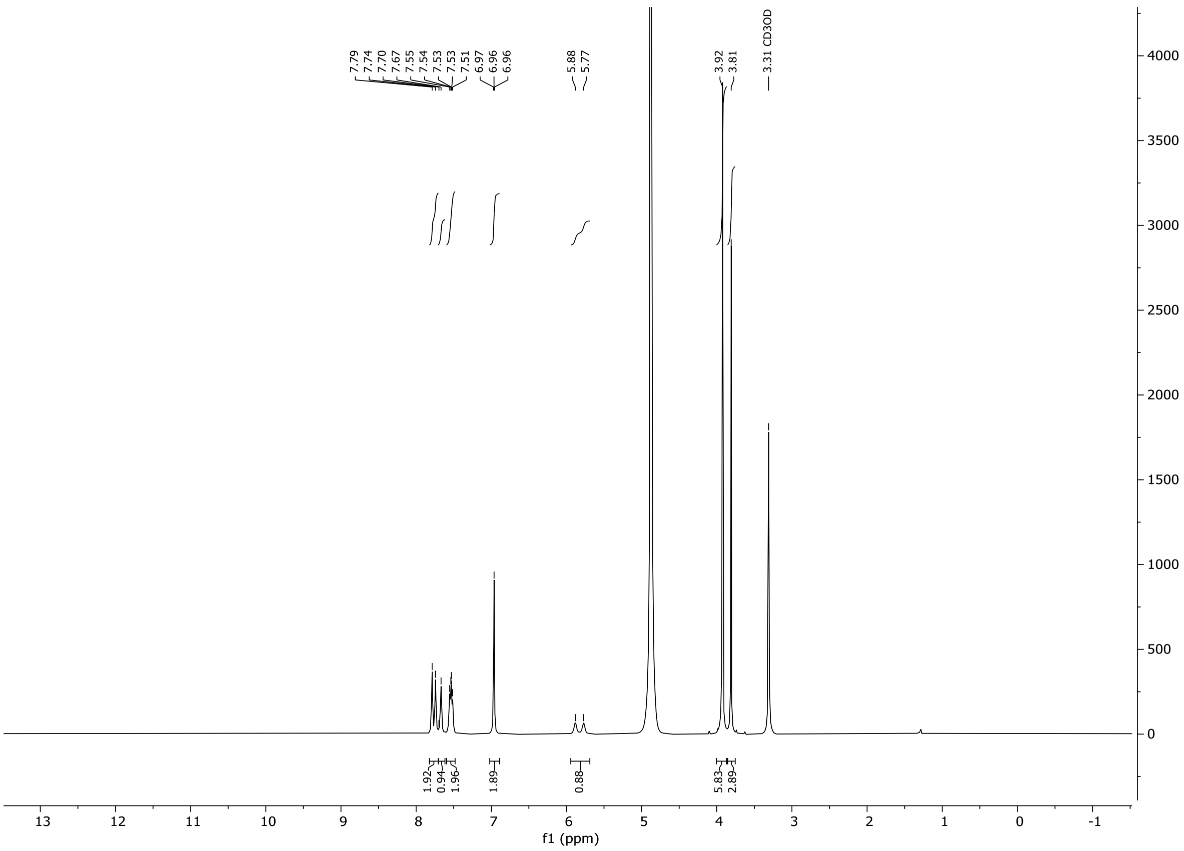

^{13}C NMR of compound **12**

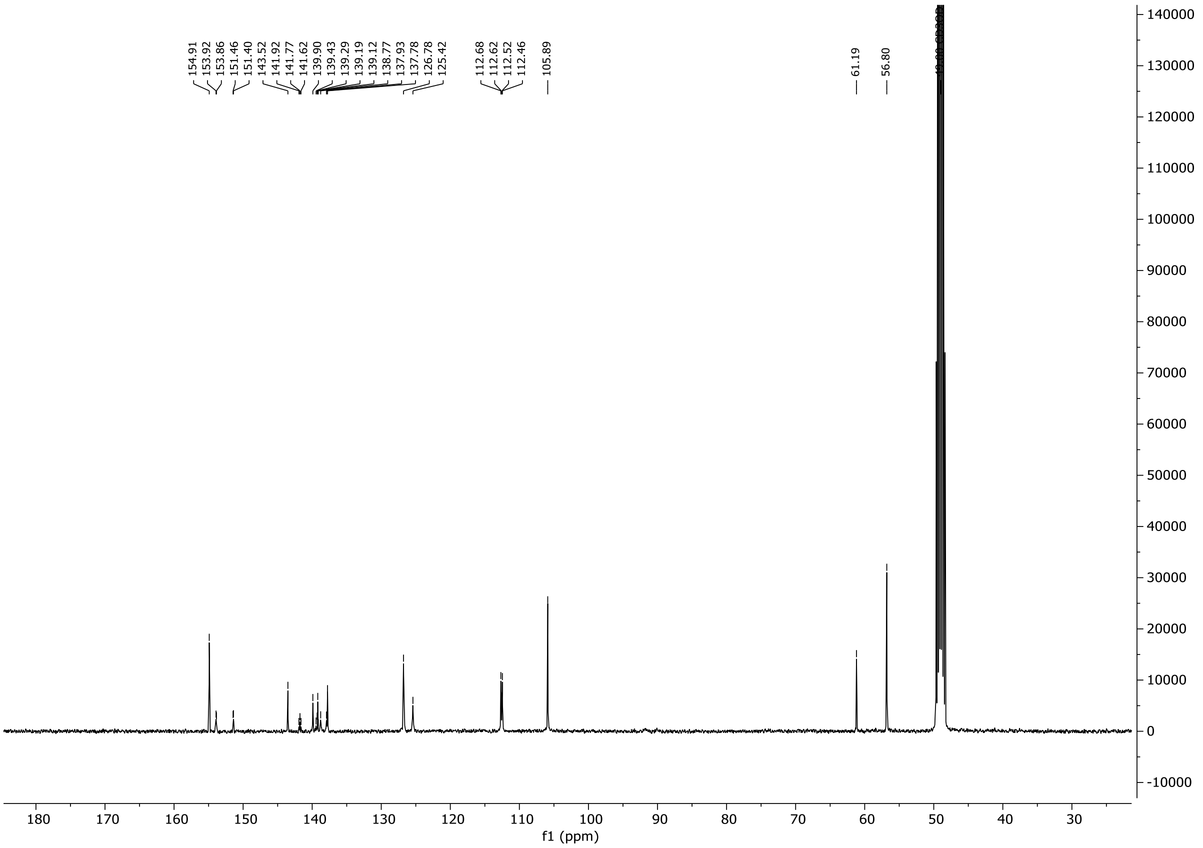

^{19}F NMR of compound **12**

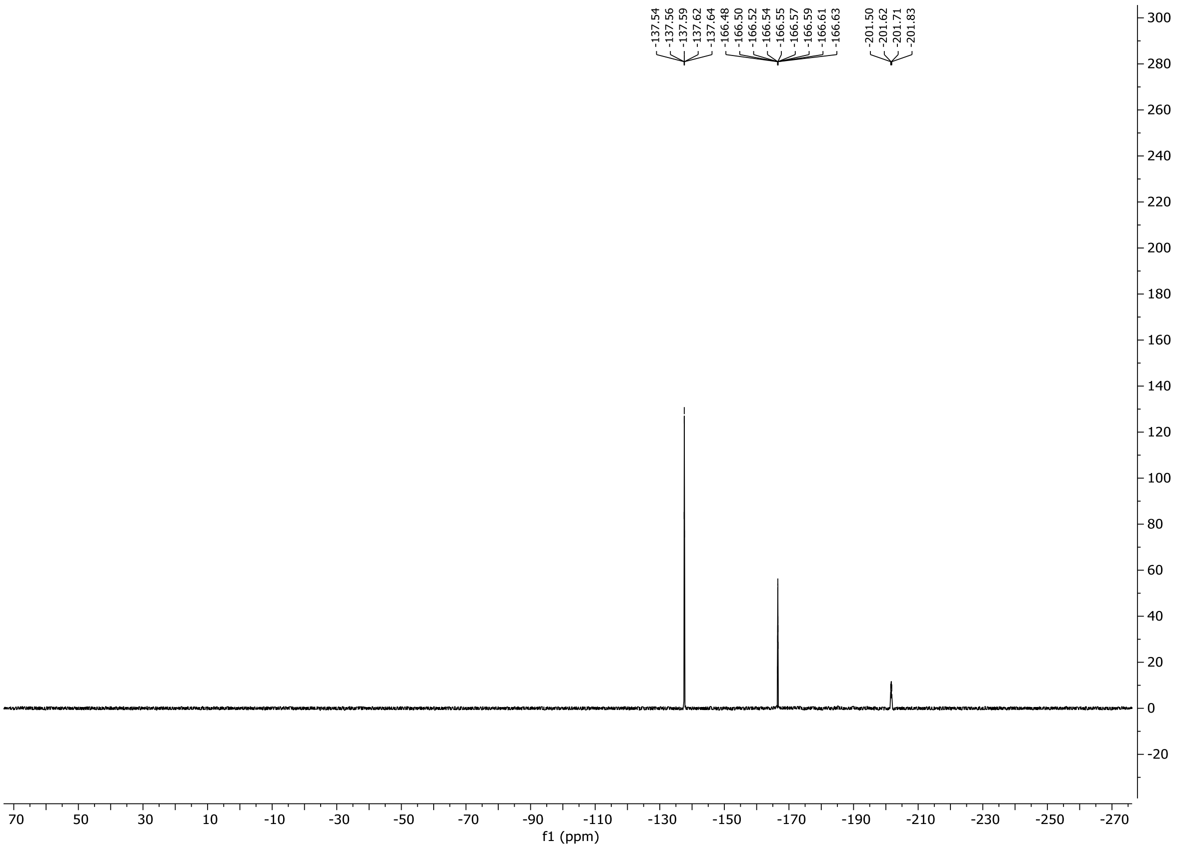

^{31}P NMR of compound **12**

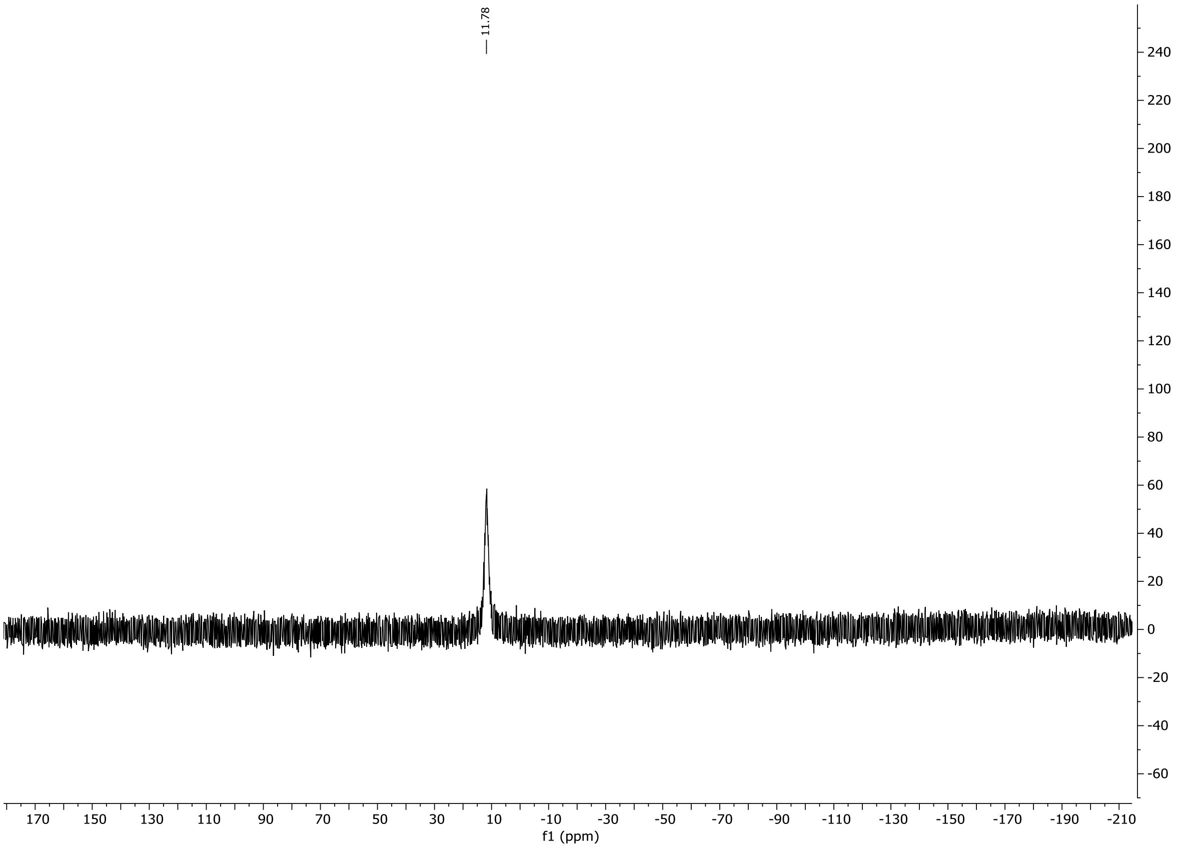

Supporting references

[1] K. Natarajan, D. Müller-Klieser, S. Rubner, T. Berg, *Chem. Eur. J.* **2020**, *26*, 148-154.

[2] Z. Nikolovska-Coleska, R. Wang, X. Fang, H. Pan, Y. Tomita, P. Li, P. P. Roller, K. Krajewski, N. G. Saito, J. A. Stuckey, S. Wang, *Anal. Biochem.* **2004**, *332*, 261-273.
